# Nickelaelectro‐Catalyzed C─H Activation to *β*‐Arylated Pyrroles *via* Multiple Dehydrogenation

**DOI:** 10.1002/anie.202510233

**Published:** 2025-09-04

**Authors:** Kazuhiro Okamoto, Simon L. Homölle, Tristan von Münchow, Sven E. Peters, Sven Trienes, João C. A. Oliveira, Lutz Ackermann

**Affiliations:** ^1^ Wöhler Research Institute for Sustainable Chemistry (WISCh) Georg‐August‐Universität Göttingen Tammannstrasse 2 37077 Göttingen Germany

**Keywords:** Electrochemistry, Heterocycles, Hydrogen, Nickel, Pyrroles, Sustainability

## Abstract

Nickel electrocatalysis has emerged as a powerful strategy for sustainable C─H activation, offering an environmentally benign alternative to traditional methods based on stoichiometric oxidants. We, herein, report a nickela‐electrocatalyzed approach for the expedient synthesis of *β*‐arylated pyrroles via a unique multiple dehydrogenative C─H activation approach. Hence, direct C─C bond formation between pyrroles and arenes was enabled, obviating the need for prefunctionalized substrates. The reaction proceeded under electrochemical conditions in a user‐friendly undivided cell, utilizing electricity as a traceless oxidant. Mechanistic studies, including cyclic voltammetry, provided support for a nickel(II/III/IV) regime, wherein anodic oxidation facilitates the dehydrogenation and C─H activation paired with the valuable hydrogen evolution reaction (HER). Our findings highlight the potential of electrocatalysis to unlock distinct reaction manifolds and provides a sustainable platform for accessing complex heteroaryl frameworks.

## Graphical abstract

Sustainable Nickelaelectro‐catalysis provided access *β*‐arylated pyrroles via an efficient anodic dehydrogenative C─H activation.

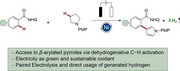

In recent decades, electrochemistry has experienced a renaissance, gaining significant attention as a powerful tool for cost‐effective and environmentally benign molecular syntheses.^[^ ^1^, ^2^, ^3^, ^4^, ^5^, ^6^, ^7^, ^8^, ^9^, ^10^, ^11^, ^12^ ^]^ One of its major advantages is the ability to eliminate the use of undesired, stoichiometric redox agents, thereby reducing waste generation. Furthermore, potentiostatic control of substrates allows for fine‐tuning of reactions and enables redox‐potential‐controlled chemoselectivities. Recent advances in metalla‐electrocatalysis have transformed the range of electrochemical reactions, enabling challenging molecular transformations with unique selectivity control, including C─H activations.^[^ ^13^, ^14^, ^15^, ^16^, ^17^, ^18^, ^19^ ^]^ In this context, Earth‐abundant 3d transition metals have emerged as highly promising catalysts due to their lower cost and reduced toxicity compared to precious metals, such as rhodium or palladium.^[^ ^20^, ^21^, ^22^, ^23^, ^24^ ^]^ Among these, nickel has shown remarkable potential as a catalyst for sustainable, yet efficient C─H activations.^[^ ^25^, ^26^, ^27^, ^28^, ^29^, ^30^ ^]^ Notably, Chatani^[^ ^31^, ^32^ ^]^ among others,^[^ ^33^, ^34^, ^35^ ^]^ made key contributions to nickel‐catalyzed C−H arylations.

On a different note, the *β*‐arylated pyrrole motif is a recurring structural feature in numerous natural products and pharmaceutical drug compounds (Scheme 1a).^[^ ^36^, ^37^, ^38^, ^39^, ^40^, ^41^ ^]^ However, the synthesis of *β*‐arylated pyrroles remains challenging.^[^ ^42^ ^]^ Previous studies demonstrated elegant strategies to achieve *β*‐selectivity through careful catalyst and ligand design, enabling the functionalization of pyrroles. Despite of these indisputable advances, these approaches typically rely on expensive precious metal catalysts, such as rhodium or palladium.^[^ ^43^, ^44^, ^45^, ^46^ ^]^ Moreover, 2,5‐disubstitution is often required to suppress undesired α‐arylation, while *β*‐arylation of nonactivated pyrroles remains rare and underexplored (Scheme 1b).^[^ ^47^ ^]^ To address these challenges, we report, herein, on the unprecedented nickel‐electrocatalyzed assembly of *β*‐arylated pyrroles via dehydrogenative C−H activation (Scheme 1c). Seminal features of our findings include a) the synthesis of complex *β*‐arylated pyrroles, b) elimination of stoichiometric chemical oxidants through protons and electrons in electricity as a green oxidant, c) detailed mechanistic insights, d) paired electrolysis, e) multiple dehydrogenation for enhanced hydrogen release by HER, and f) a gram‐scale synthesis, highlighting the synthesis utility of our approach.

**Scheme 1:**
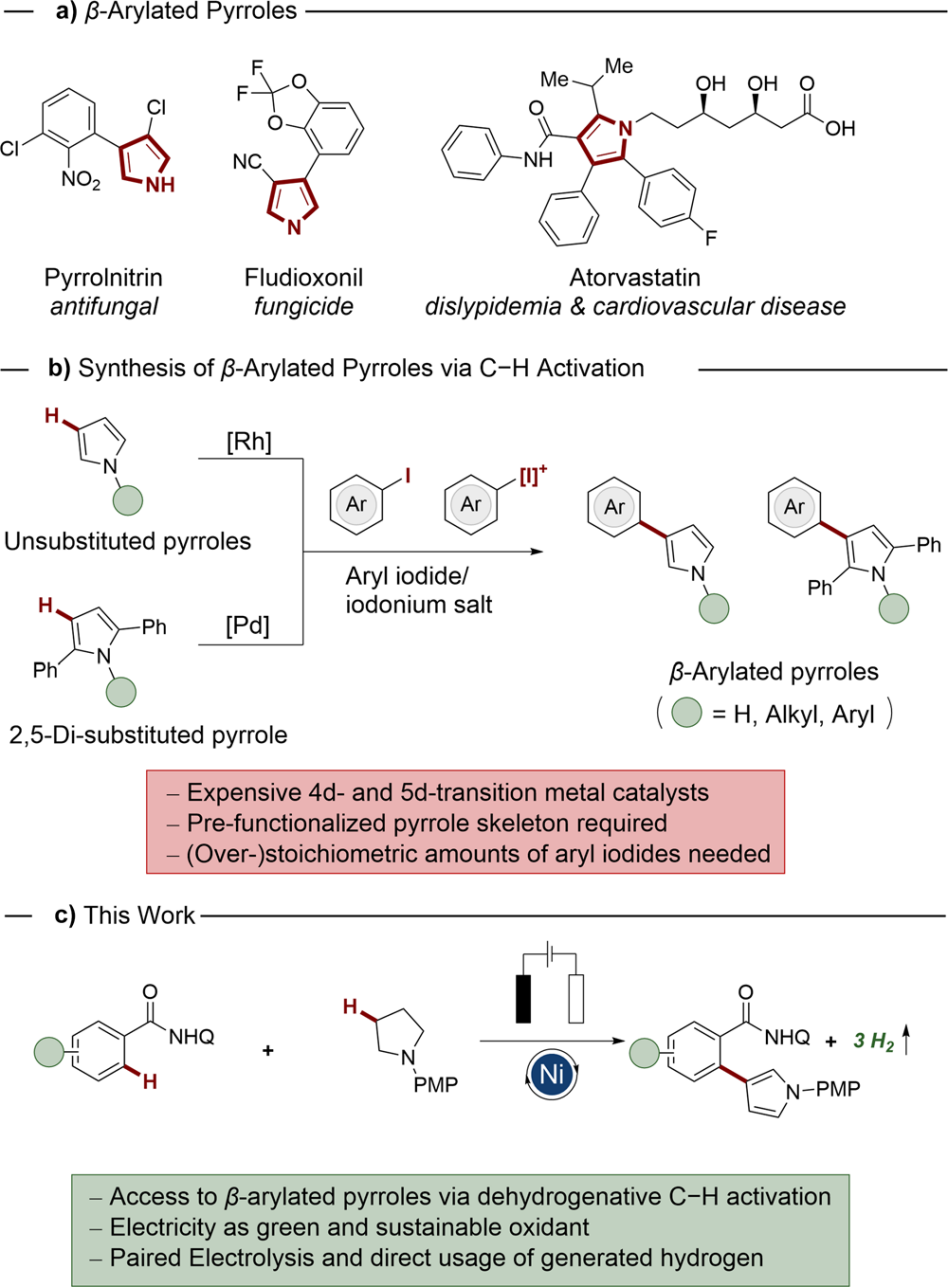
a) Naturally occurring *β*‐arylated pyrroles. b) Precedent studies for *β*‐C−H arylation. c) Nickelaelectro‐catalyzed synthesis of *β*‐arylated pyrroles.

## Reaction Optimization

We initiated our studies (Table 1) using benzamide **1a** and pyrrolidine **2** as substrates with the aid of glassy carbon (GC) and graphite felt (GF) electrodes, as cathode and anode, respectively, in *N*,*N*‐dimethylacetamide with LiClO_4_ as the electrolyte, sodium adamantyl carboxylate^[^ ^48^ ^]^ as the additive and Ni(dme)Cl_2_ as the precatalyst in a user‐friendly undivided cell. Thereby, we obtained 31% of the corresponding *β*‐arylated pyrrole **3**. The replacement of the GC cathode with GF led to a drastic decrease in the reaction's efficacy (entry 2). Probing various electrolytes showed that TBAPF_6_ was more effective, leading to the desired product **3** in 45% yield (entry 3). Applying a constant current of 8.0 mA further improved the yield up to 64% (entry 4). Under otherwise identical reaction conditions, benzyl‐, triphenylmethyl‐, and *tert*‐butyloxycarbonyl‐substituted pyrrolidines gave thus far less satisfactory results. In contrast, 2,4‐dimethoxyphenyl pyrrolidine furnished the corresponding arylated pyrrole **34** (see Supporting Information). Additionally, the pyrrolidine **2** was partially transformed into the corresponding pyrrole by dehydrogenation as a side product. Generally, the nickela‐electrocatalysis was characterized by high levels of mono‐chemoselectivity. Reducing or extending the reaction time did not prove to be beneficial (entry 5). Changing the scale gave no change in the yield (entry 6). Neither the increase or decrease of the applied current altered the catalytic performance (entry 7). Adjusting the stoichiometry of pyrrolidine **2** had only a minor impact (entry 9). Several nickel sources were probed, reflecting the beneficial features of Ni(dme)Cl_2_ (entry 10). Control experiments in the absence of nickel, electricity, or base confirmed their crucial roles in the metalla‐electrocatalysis (entry 11).

**Table 1:** Reaction development.

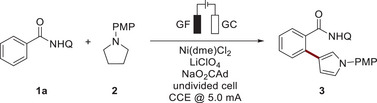		
Entry	Deviation from standard conditions	Yield **3** (%)^a)^
1	None	31
2	GF cathode	22
3	TBAPF_6_ as electrolyte	45
**4**	**CCE @ 8.0 mA**	**64** (**61**)
5	8 h/24 h	32/40
6	0.15 mmol **1a**	62
7	CCE @ 4.0 mA/10.0 mA	16/26
8	120 °C/160 °C	–/67
9	3.0 equiv./4.0 equiv. of **2**	68/42
10	Ni(dme)Br_2_/NiCl_2_ as [Ni]	19/–
11	No [Ni], no electricity, no NaO_2_CAd	–/–/–

^a)^ Reaction conditions: **1a** (0.25 mmol), **2** (0.50 mmol), Ni(dme)Cl_2_ (10 mol%), electrolyte (1.6 equiv.), NaO_2_CAd (2.0 equiv.), DMA (4.0 mL), CCE @ 8.0 mA, 140 °C, 17 h, undivided cell with glassy carbon (GC) cathode (10 mm × 25 mm × 0.25 mm), graphite felt (GF) anode (10 mm × 15 mm × 6 mm), under N_2_. Yields were determined by ^1^H‐NMR using 1,3,5‐trimethoxybenzene as internal standard. Isolated yield is given in parentheses. DMA = *N*,*N*‐dimethylacetamide, PMP = *para*‐methoxyphenyl, dme = ethylene glycol dimethyl ether, TBA = tetra‐*n*‐butylammonium, Q = 8‐quinoline.

**Scheme 2:**
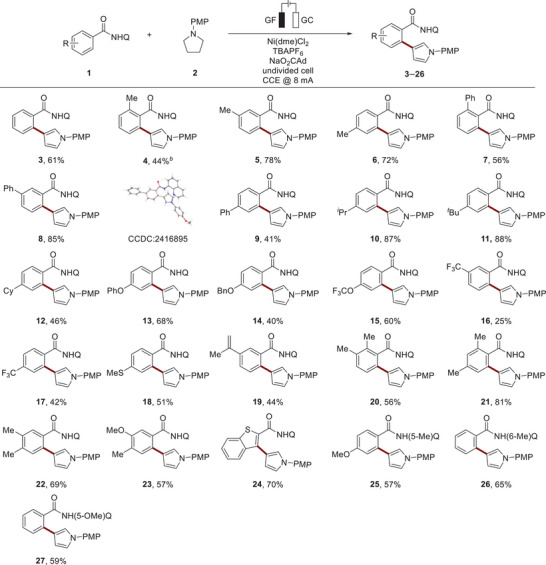
Versatility for the synthesis of *β*‐arylated pyrroles.^a)^Reaction conditions: **1** (0.25 mmol), **2** (0.50 mmol), Ni(dme)Cl_2_ (10 mol %), TBAPF_6_ (1.6 equiv.), NaO_2_CAd (2.0 equiv.), DMA (4.0 mL), CCE @ 8.0 mA, 140 °C, 17 h, undivided cell with glassy carbon (GC) cathode (10 mm × 25 mm × 0.25 mm), graphite felt (GF) anode (10 mm × 15 mm × 6 mm), under N_2_. Isolated yields are shown, ^b)^Ni(dme)Cl_2_ (20 mol%). DMA = *N*,*N*‐dimethylacetamide, PMP = *para*‐methoxyphenyl, dme = ethylene glycol dimethyl ether, TBA = tetra‐*n*‐butylammonium, Q = 8‐quinoline.

With the optimized reaction conditions in hand, we next explored the viable substrate scope (Scheme 2). Alkyl and aryl‐substituents in the *ortho‐*, *meta‐*, or *para*‐positions were well tolerated to give the desired products **4**–**6** and **7**–**9**, respectively. The connectivity of the β‐substituted pyrrole was unambiguously confirmed for product **8** through single‐crystal X‐ray diffraction analysis (CCDC: 2416895). Electron‐donating groups in the *para*‐position (**10**–**14**) gave rise to the desired products in up to 88% yield, whereas the presence of electron‐withdrawing groups, such as CF_3_, led to a slight decrease in the performance (**15–** **17**). To our delight, oxidation‐sensitive thioether (**18**) as well as an alkenyl substituent (**19**), remained intact, highlighting the chemo‐selectivity of our transformation. Subsequently, a variety of substituted substrates were evaluated, resulting in the desired products **20**–**23**. In addition, we demonstrated the compatibility of our method with heterocyclic compounds for benzothiophene, affording the desired product **24**. Finally, we explored different substitution patterns on the quinoline group, leading to the successful formation of the target products **25**–**27**.

**Scheme 3:**
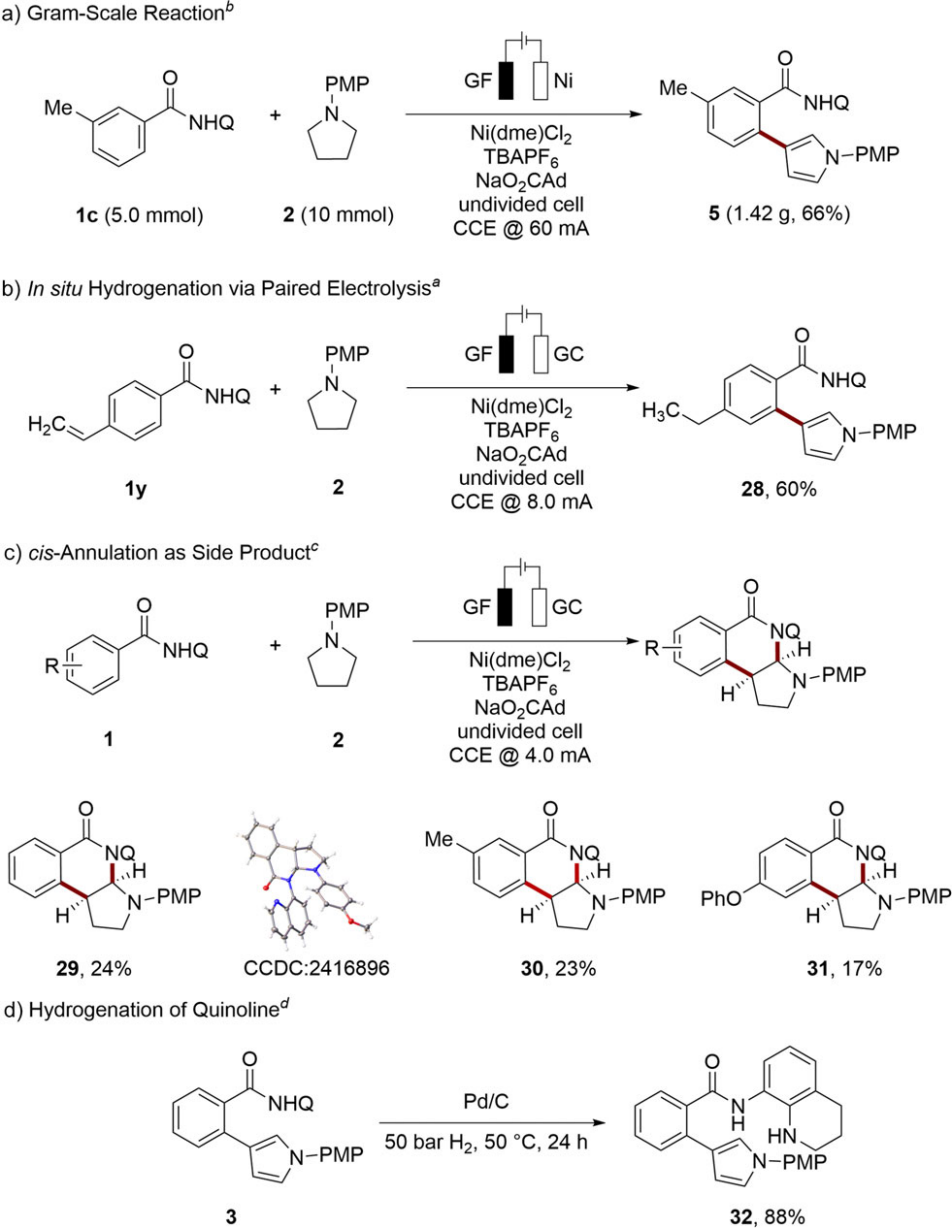
Applications of the nickela‐electrocatalysis. ^a)^Reaction conditions: **1a** (0.25 mmol), **2** (0.50 mmol), Ni(dme)Cl_2_ (10 mol %), TBAPF_6_ (1.6 equiv.), NaO_2_CAd (2.0 equiv.), DMA (4.0 mL), CCE @ 8.0 mA, 140 °C, 17 h, undivided cell with glassy carbon (GC) cathode (10 mm × 25 mm × 0.25 mm), graphite felt (GF) anode (10 mm × 15 mm × 6 mm), under N_2_. Isolated yields are shown. ^b)^CCE @ 60.0 mA, 140 °C, 48 h, undivided cell with nickel foam cathode (20 mm × 20 mm × 1.4 mm), GF anode (30 mm × 30 mm ×  6 mm). ^c)^CCE @ 4.0 mA. DMA = *N*,*N*‐dimethylacetamide. ^d)^Pd/C (10 mol%), 50 bar H_2_, 50 °C, 24 h. PMP = *para*‐methoxyphenyl, dme = ethylene glycol dimethyl ether, TBA = tetra‐*n*‐butylammonium, Q = 8‐quinoline.

To assess the practicality and robustness of our method, we conducted a gram‐scale reaction utilizing a cost‐effective nickel‐foam cathode (Scheme 3a). We were pleased to obtain the isolated product **5** in 66% yield. Additionally, we explored the potential of our dehydrogenative approach for transfer hydrogenation. Hence, we employed substrate **1y**, featuring an additional alkene in a remote position. Remarkably, we successfully obtained the hydrogenation product **28** through paired electrolysis, reflecting the distinctive capabilities of electrochemically mediated reactivity by exploiting both anodic and cathodic transformations (Scheme 3b). Furthermore, we discovered a current‐modulated switch in chemoselectivity. Thus, through galvanostatic electrolysis at 4.0 mA the formation of the annulated products **29**–**31** was favored, demonstrating how the tunability of our electrochemical strategy can reveal previously unexplored reaction pathways (Scheme 3c).^[^ ^49^, ^50^ ^]^ Further derivatization of the thus obtained product **3** gave the Fsp^3^‐enriched tetrahydroquinoline **32** through hydrogenation.

**Scheme 4:**
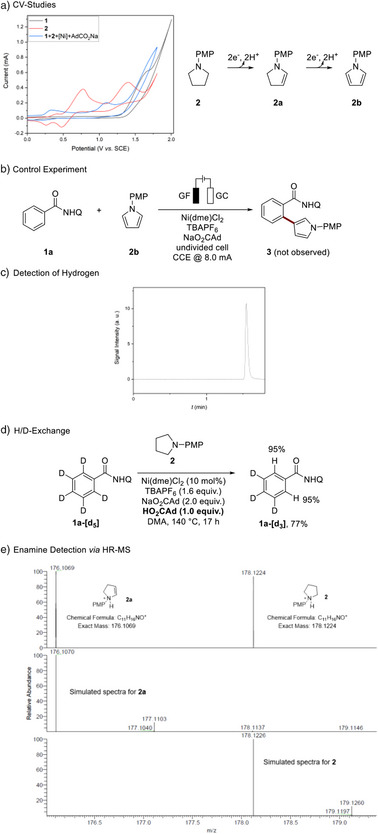
Mechanistic experiments: a) cyclovoltammetry studies, at room temperature, working electrode GC, Pt‐wire as counter electrode and SCE electrode as reference. TBAPF_6_ (0.1 m) as electrolyte and a scan‐rate of 100 m V s^−1^; b) control experiment; c) detection of molecular hydrogen as byproduct; d) hydrogen isotope exchange (HIE); e) intermediate detection by HR‐MS. ^a)^CV of **1**+[Ni]+AdCO_2_Na was measured after preheating at 140 °C for 30 min. Reaction conditions for b): **1a** (0.25 mmol), **2b** (0.50 mmol), Ni(dme)Cl_2_ (10 mol%), TBAPF_6_ (1.6 equiv.), NaO_2_CAd (2.0 equiv.), DMA (4.0 mL), CCE @ 8.0 mA, 140 °C, 17 h, undivided cell with glassy carbon (GC) cathode (30 mm × 25 mm × 0.25 mm), graphite felt anode (10 mm × 15 mm × 6 mm), under N_2_, abbreviated reaction conditions for d): **1a[d_5_]** (0.25 mmol), **2** (0.50 mmol), HO_2_CAd (1.0 equiv.). Isolated yields are shown. DMA = *N*,*N*‐dimethylacetamide, PMP = *para*‐methoxyphenyl, dme= ethylene glycol dimethyl ether, TBA = tetra‐*n*‐butylammonium, Q=8‐quinoline.

Subsequently, we conducted cyclic voltammetry (CV) studies, as illustrated in Scheme 4a. In the cyclic voltammogram of substrate **2**, two distinct oxidation waves were observed (Scheme 4a, red line). Pyrrolidine **2** appears to undergo a two‐step oxidation process,^[^ ^51^, ^52^ ^]^ first furnishing the intermediate enamine **2a**, along with its subsequent conversion to pyrrole **2b**. The in situ prepared nickel complex **A** exhibits two oxidation events at +0.3 V and +1.1 V (Scheme 4a, blue line, V versus SCE). Consistent with our previous findings,^[^ ^28^ ^]^ we assigned the electrochemical oxidation of nickel(II) **A** to yield nickel(III) **B** and nickel(IV) **C** species at approximately −0.2 and +0.5 V, respectively. Alternatively, the second wave may be ascribed to the pyrrolidine oxidation upon coordination to the nickel catalyst.

Additionally, we conducted a control experiment (Scheme 4b) using pyrrole **2b** as the coupling partner in lieu of substrate **2**. However, the desired product was not observed here. Finally, we performed a GC‐TCD analysis of the headspace during the electrolysis, which confirmed the generation of molecular hydrogen as the in situ produced gas (Scheme 4c). In addition to the qualitative analysis of the hydrogen formation, we conducted quantification studies (for further information see Supporting Information). Further, we explored a viable hydrogen isotope D/H exchange (HIE) without electricity, with 95% of hydrogen incorporation being observed. Hence, carboxylate‐assisted C─H activation is proposed to be fast and reversible. Detailed mechanistic studies by high‐resolution mass spectrometry (HR‐MS) provided strong support for enamine **2a** (as well as enamine **2c**, see Supporting Information) as the key intermediate.

**Scheme 5:**
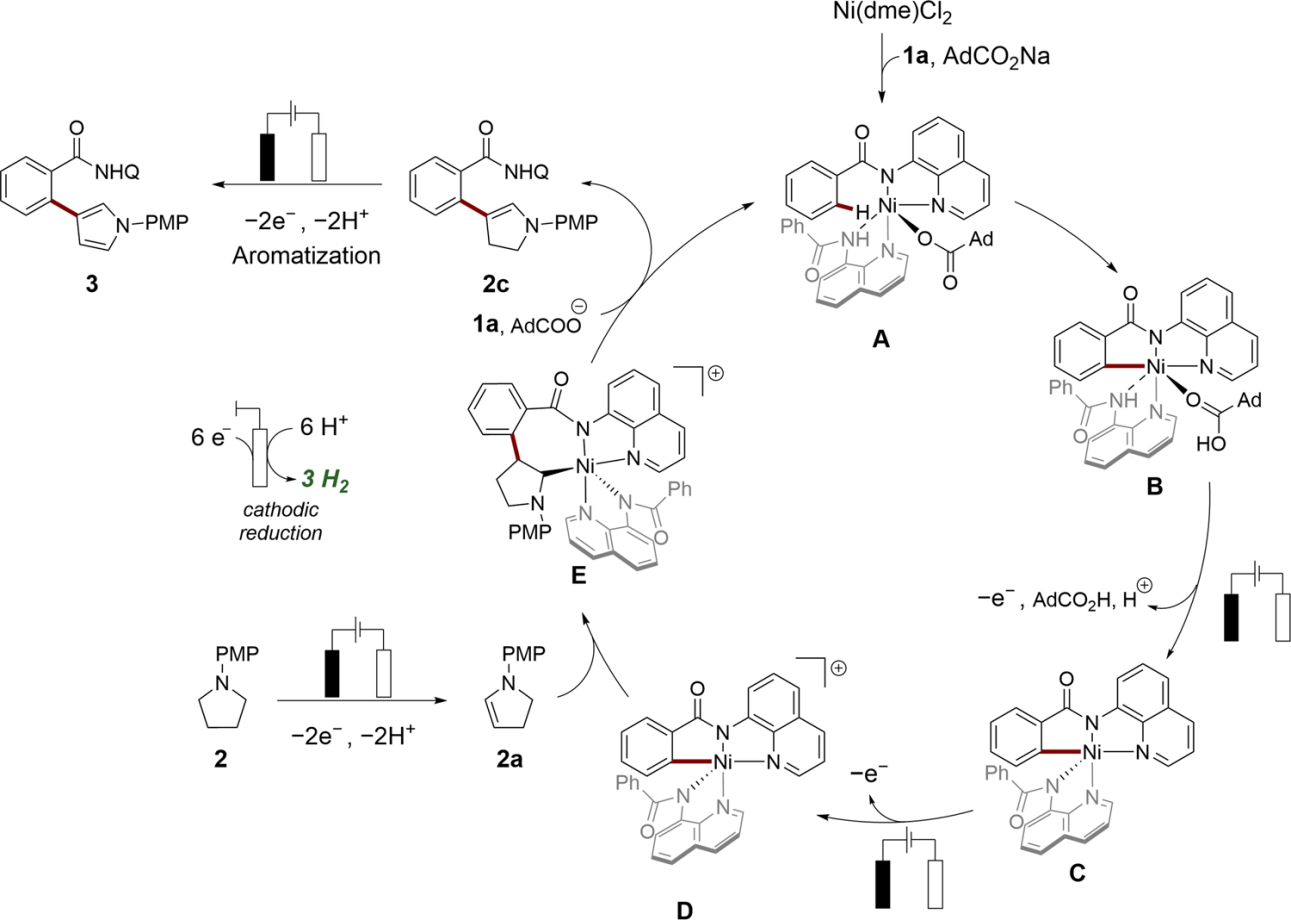
Plausible reaction mechanism.

Based on our mechanistic insights and established literature precedents,^[^ ^50^, ^53^ ^]^ we propose a plausible reaction mechanism, depicted in Scheme 5. The electrocatalysis commences with the coordination of Ni(dme)Cl_2_ by the benzamide **1a**, forming the catalytically active species **A**. Carboxylate‐assisted BIES (base‐assisted internal electrophilic substitution) C─H activation then generates intermediate **B**, which undergoes two successive electrochemical oxidations to yield intermediates **C** and **D**, respectively. Simultaneously, pyrrolidine **2** is oxidized by PCET to form the enamine **2a**, which reacts with metallacycle **D** to form intermediate **E**. *β*‐Elimination subsequently releases enamine **2c** and regenerates the active species **A**. In contrast, C−N reductive elimination from intermediate **E** likely accounts for the formation of annulated side‐product **29**.^[^ ^49^ ^]^ Finally, enamine **2c** undergoes subsequent oxidation to yield the desired *β*‐arylated pyrrole product **3**.

## Conclusion

In summary, we have reported on an electrochemical strategy for the efficient synthesis of *β*‐arylated pyrroles via a multistep dehydrogenative nickela‐electrocatalyzed C─H activation with ample scope. Mechanistic studies reveal an anodic enamine formation followed by migratory insertion into a cyclometallated nickel complex. The robustness of this approach was highlighted by its scalability and its successful application towards paired electrolysis. Our findings not only provide a straightforward method for accessing functionalized pyrroles in a site‐selective manner, but also offer valuable insights into nickela‐electrocatalysis.

## Conflict of Interests

The authors declare no conflict of interest.

## Supporting information



Supporting Information

Supporting Information

## Data Availability

The data that support the findings of this study are available in the Supporting Information of this article.
