# Supplementary material for: Nickelaelectro‐Catalyzed C─H Activation to β‐Arylated Pyrroles via Multiple Dehydrogenation
Source: Angew Chem Int Ed Engl. 2025 Sep 4;64(40):e202510233. doi: 10.1002/anie.202510233 (PMC12462739; doi:10.1002/anie.202510233)
Supplement: Supplementary file 1 — Supporting Information [file ANIE-64-e202510233-s002.pdf]

Supporting Information

Nickelaelectro-Catalyzed C−H Activation to *β*-Arylated Pyrroles via Multiple Dehydrogenation

Kazuhiro Okamoto‡, Simon Lukas Homölle‡, Tristan von Münchow, Sven Erik Peters, Sven Trienes, João C. A. Oliveira, and Lutz Ackermann*

Wöhler Research Institute for Sustainable Chemistry (WISCh) and Institut für Organische und Biomolekulare Chemie, Georg-August-Universität Göttingen, Tammannstraße 2, 37077 Göttingen, Germany

Table of Contents

I. Experimental Section 2
II. Preliminary Optimization for Electrochemical Reactions 7
III. General Procedures 9
IV. Characterization Data of Products 10
V. Unsuccessful Examples 26
VI. Gram-Scale Reaction 27
VII. CV Studies 28
VIII. H/D Exchange 31
IX. Gas Measurement Studies 32
X. Crystallographic Data of **8** and **29** 34
XI. ^{1}H and ^{13}C-NMR Spectra of Compounds 43
XII. References 83

I. Experimental Section

General information

Solvents for column chromatography and extraction (EtOAc, *n*-hexane, DCM etc.) were distilled before their use. Routine TLC analysis was carried out on aluminium sheets coated with silica gel 60 F254, 0.2 mm thickness, analyzing by a 254 nm UV lamp. Column chromatography was performed using Merck silica gel 60 (40–63 µm). All reagents and solvents were purchased from commercial suppliers (Aldrich, Alfa, TCI, BLD, etc.) and used as supplied unless otherwise stated.

NMR spectra were recorded on the Bruker Avance Neo 300 and 400 MHz spectrometers. Chemical shifts are stated as δ-values in parts per million (ppm) referenced to the residual proton peak of the deuterated solvent (^{1}H; $CDCl_3$: 7.26 ppm) or the carbon peak of the solvent (^{13}C: $CDCl_3$: 77.16 ppm). Data are reported as follows: chemical shift (δ) and coupling constants J are reported in Hertz (Hz).

IR spectra were recorded on a Bruker FT-IR Alpha-P device equipped with an iD7 ATR detector and were recorded in the range from 4000 to 400 cm^{-1}. CV studies were performed using a Metrohm Autolab PGSTAT204 workstation and Nova 2.1 software.

High-Resolution mass spectrometry (HR–MS) was recorded on Exactive Plus, 2.9 Build 290492 by Thermo Fisher Scientific.

Abbreviations

Ac, acetyl; Ad, adamantyl; Bu, butyl; CCE, constant current electrolysis; DMA, *N*, *N*-dimethylacetamide; dme, 1, 2-dimethoxyethane; DMF, *N*, *N*-dimethylformamide; DMSO, dimethylsulfoxide, GC, glassy carbon; GF, graphite felt; Me, methyl; PMP, *p*-methoxyphenyl

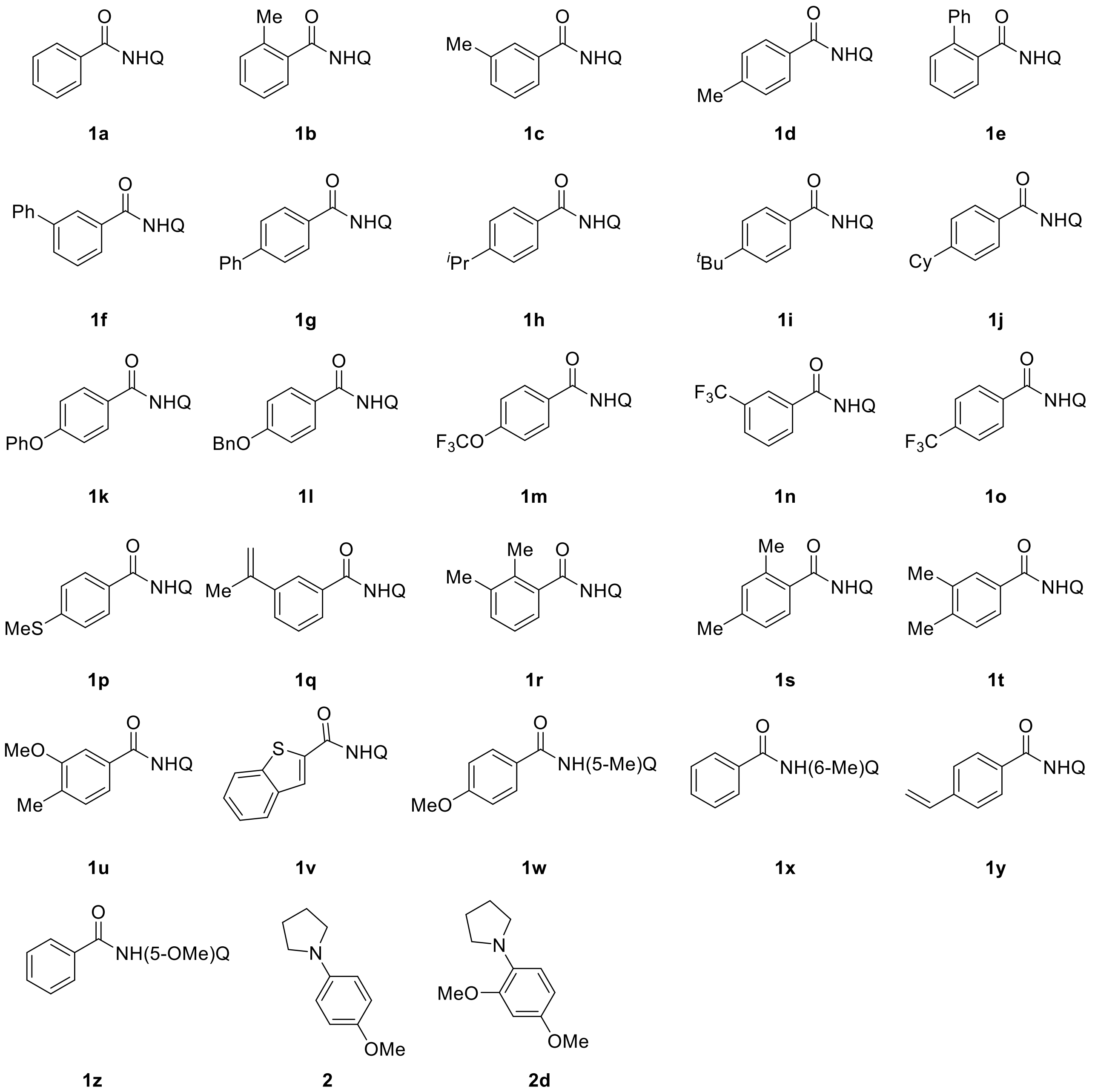

Figure S1. Structure of benzamides and pyrrolidines used for substrate scope.

Amides **1a–1i**, **1m–1p**, **1r–1t**, **1v**,**1x–1z** and amines **2** and **2d** were synthesized according to the literature.[1-9]

4-Cyclohexyl-*N*-(quinolin-8-yl)benzamide (**1j**):

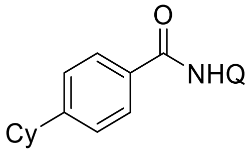

1j was prepared according to the literature.[10]

M. p.: 114–116 °C.

^{1}H-NMR (400 MHz, $CDCl_3$) δ = 10.72 (s, 1H), 8.94 (dd, J = 7.6, 1.4 Hz, 1H), 8.85 (dd, J = 4.2, 1.7 Hz, 1H), 8.18 (dd, J = 8.3, 1.7 Hz, 1H), 8.04–7.90 (m, 2H), 7.59 (t, J = 7.9 Hz, 1H), 7.53 (dd, J = 8.3, 1.4 Hz, 1H), 7.47 (dd, J = 8.3, 4.2 Hz, 1H), 7.42–7.32 (m, 2H), 2.61 (ddd, J = 11.6, 8.2, 3.4 Hz, 1H), 1.94–1.82 (m, 4H), 1.78 (ddq, J = 12.8, 3.3, 1.6 Hz, 1H), 1.55–1.39 (m, 4H), 1.30 (ddd, J = 16.3, 9.7, 3.4 Hz, 1H).

^{13}C-NMR (101 MHz, $CDCl_3$) δ = 165.7 (C_q), 152.4 (C_q), 148.4 (CH), 139.0 (C_q), 136.5 (CH), 134.9 (C_q), 132.9 (C_q), 128.1 (C_q), 127.6 (CH), 127.5 (CH), 127.4 (CH), 121.8 (CH), 121.6 (CH), 116.6 (CH), 44.7 (CH), 34.4 (CH_2), 26.9 (CH_2), 26.2 (CH_2).

IR (ATR): 3356, 2923, 2850, 1671, 1609, 1527, 1484, 1424, 1384, 1327 cm^{-1}.

HR-MS (ESI): *m/z* calcd for $C_{22}H_{23}N_2O$ $[M+H]^+$ 331.1805, found 331.1802.

4-Phenoxy-*N*-(quinolin-8-yl)benzamide (**1k**):

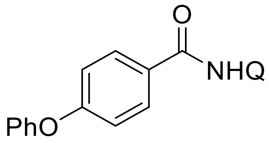

1k was prepared according to the literature.[10]

M. p.: 115–117 °C.

^{1}H-NMR (400 MHz, $CDCl_3$) δ = 10.70 (s, 1H), 8.93 (dd, J = 7.5, 1.5 Hz, 1H), 8.82 (dd, J = 4.2, 1.7 Hz, 1H), 8.16 (dd, J = 8.3, 1.7 Hz, 1H), 8.11–7.97 (m, 2H), 7.61–7.55 (m, 1H), 7.52 (dd, J = 8.3, 1.5 Hz, 1H), 7.45 (dd, J = 8.3, 4.2 Hz, 1H), 7.42–7.38 (m, 2H), 7.20 (td, J = 7.3, 1.2 Hz, 1H), 7.16–7.08 (m, 4H).

^{13}C-NMR (101 MHz, $CDCl_3$) δ = 164.8 (C_q), 161.0 (C_q), 156.0 (C_q), 148.3 (CH), 138.8 (C_q), 136.5 (CH), 134.7 (C_q), 130.1 (CH), 129.6 (C_q), 129.4 (CH), 128.1 (C_q), 127.6 (CH), 124.5 (CH), 121.8 (CH), 121.6 (CH), 120.1 (CH), 118.0 (CH), 116.5 (CH).

IR (ATR): 3355, 3015, 1670, 1586, 1529, 1504, 1499, 1424, 1385, 1241 cm^{-1}.

HR-MS (ESI): *m/z* calcd for $C_{22}H_{17}N_2O_2$ $[M+H]^+$ 341.1285, found 341.1282.

4-(Benzyloxy)-*N*-(quinolin-8-yl)benzamide (**1l**):

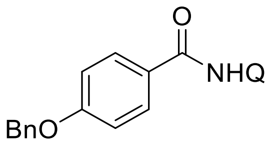

1l was prepared according to the literature.[10]

M. p.: 160–162 °C.

^{1}H-NMR (400 MHz, $CDCl_3$) δ = 10.68 (s, 1H), 8.94 (dd, J = 7.6, 1.4 Hz, 1H), 8.84 (dd, J = 4.2, 1.7 Hz, 1H), 8.15 (dd, J = 8.3, 1.7 Hz, 1H), 8.10–8.01 (m, 2H), 7.58 (t, J = 7.9 Hz, 1H), 7.51 (dd, J = 8.3, 1.4 Hz, 1H), 7.49–7.39 (m, 5H), 7.38–7.33 (m, 1H), 7.15–7.05 (m, 2H), 5.15 (s, 2H).

^{13}C-NMR (101 MHz, $CDCl_3$) δ = 165.0 (C_q), 161.7 (C_q), 148.3 (CH), 138.8 (C_q), 136.4 (CH), 134.8 (C_q), 129.3 (CH), 128.8 (CH), 128.3 (CH), 128.1 (C_q), 127.8 (C_q), 127.6 (CH), 121.7 (CH), 121.5 (CH), 116.4 (CH), 114.9 (CH), 70.2 (CH_2).
IR (ATR): 3358, 3059, 1666, 1604, 1529, 1508, 1495, 1384, 1327, 1248 cm^{-1}.
HR-MS (ESI): *m/z* calcd for $C_{23}H_{19}N_2O_2$ $[M+H]^+$ 355.1441, found 355.1438.

3-(Prop-1-en-2-yl)-*N*-(quinolin-8-yl)benzamide (**1q**):

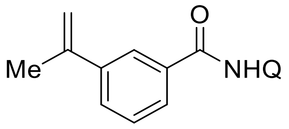

1q was prepared according to the literature.[10]
M. p.: 82–84 °C.
^{1}H-NMR (400 MHz, $CDCl_3$) δ = 10.75 (s, 1H), 8.95 (dd, J = 7.6, 1.4 Hz, 1H), 8.83 (dd, J = 4.2, 1.7 Hz, 1H), 8.19 (t, J = 1.9 Hz, 1H), 8.16 (dd, J = 8.3, 1.7 Hz, 1H), 7.95 (ddd, J = 7.6, 1.8, 1.1 Hz, 1H), 7.68 (ddd, J = 7.8, 1.9, 1.1 Hz, 1H), 7.62–7.56 (m, 1H), 7.55–7.51 (m, 1H), 7.51–7.42 (m, 2H), 5.51 (t, J = 1.1 Hz, 1H), 5.20 (t, J = 1.5 Hz, 1H), 2.24 (dd, J = 1.5, 0.8 Hz, 3H).
^{13}C-NMR (101 MHz, $CDCl_3$) δ = 165.6 (C_q), 148.4 (CH), 142.6 (C_q), 142.0 (C_q), 138.9 (C_q), 136.5 (CH), 135.3 (C_q), 134.7 (C_q), 128.9 (CH), 128.7 (CH), 128.1 (C_q), 127.5 (CH), 125.9 (CH), 124.9 (CH), 121.8 (CH), 116.6 (CH), 113.8 (CH_2), 21.9 (CH_3).
IR (ATR): 3354, 1673, 1578, 1529, 1483, 1424, 1386, 1329, 1250, 1231 cm^{-1}.
HR-MS (ESI): *m/z* calcd for $C_{19}H_{17}N_2O$ $[M+H]^+$ 289.1335, found 289.1334.

3-Methoxy-4-methyl-*N*-(quinolin-8-yl)benzamide (**1u**):

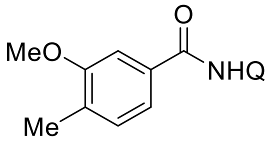

1u was prepared according to the literature.[10]
M. p.: 157–159 °C.
^{1}H-NMR (400 MHz, $CDCl_3$) δ = 10.75 (s, 1H), 8.94 (dd, J = 7.5, 1.4 Hz, 1H), 8.86 (dd, J = 4.2, 1.7 Hz, 1H), 8.19 (dd, J = 8.3, 1.6 Hz, 1H), 7.64–7.57 (m, 2H), 7.57–7.52 (m, 2H), 7.48 (dd, J = 8.3, 4.2 Hz, 1H), 7.29 (dd, J = 7.6, 0.9 Hz, 1H), 3.96 (s, 3H), 2.31 (s, 3H).
^{13}C-NMR (101 MHz, $CDCl_3$) δ = 165.7 (C_q), 158.3 (C_q), 148.4 (CH), 139.0 (C_q), 136.5 (CH), 134.8 (C_q), 134.2 (C_q), 131.4 (C_q), 130.7 (CH), 128.2 (C_q), 127.7 (CH), 121.8 (CH), 121.7 (CH), 118.6 (CH), 116.6 (CH), 109.5 (CH), 55.7 (CH_3), 16.6 (CH_3).
IR (ATR): 3356, 1663, 1579, 1540, 1509, 1492, 1465, 1390, 1329, 1272 cm^{-1}.
HR-MS (ESI): *m/z* calcd for $C_{18}H_{17}N_2O_2$ $[M+H]^+$ 293.1285, found 293.1283.

4-Methoxy-*N*-(5-methylquinolin-8-yl)benzamide (**1w**):

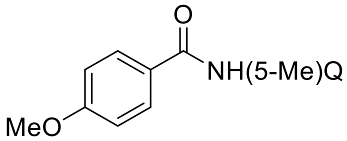

1w was prepared according to the literature.[10]

M. p.: 94–96 °C.

^{1}H-NMR (400 MHz, $CDCl_3$) δ = 10.62 (s, 1H), 8.80 (d, J = 1.7 Hz, 1H), 8.75 (dd, J = 4.2, 1.6 Hz, 1H), 8.06 (dd, J = 8.8, 2.2 Hz, 3H), 7.41 (dd, J = 8.3, 4.2 Hz, 1H), 7.28 (dd, J = 1.9, 1.0 Hz, 1H), 7.03 (d, J = 8.9 Hz, 2H), 3.88 (s, 3H), 2.56 (d, J = 1.0 Hz, 3H).

^{13}C-NMR (101 MHz, $CDCl_3$) δ = 165.1 (C_q), 162.6 (C_q), 147.4 (CH), 137.8 (C_q), 137.6 (C_q), 135.7 (CH), 134.4 (C_q), 129.3 (CH), 128.2 (C_q), 127.6 (C_q), 121.8 (CH), 120.5 (CH), 118.5 (CH), 114.1 (CH), 55.6 (CH_3), 22.5 (CH_3).

IR (ATR): 3354, 1668, 1636, 1535, 1509, 1476, 1426, 1256, 1176, 1028 cm^{-1}.

HR-MS (ESI): *m/z* calcd for $C_{18}H_{17}N_2O_2$ $[M+H]^+$ 293.1285, found 293.1283.

II. Preliminary Optimization for Electrochemical Reactions

Table S1. Screening for electrode materials.

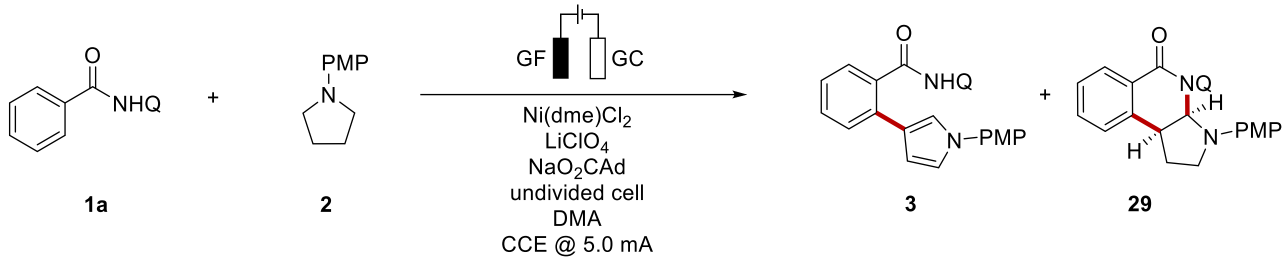

Entry	Deviation of electrode	Yield% (**3**/**29**)[a]
1	none	31/---
2	GF anode	20/ 13
3	Pt anode	---/ 8
4	Pt cathode	26/ 6

Table S2. Screening for electrolyte.

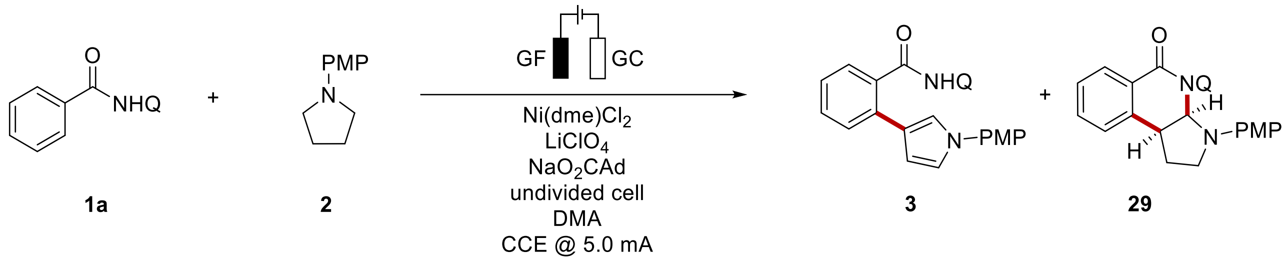

Entry	Deviation of electrolyte	Yield% (**3**/**29**)[a]
1	none	31/ ---
2	$TBAClO_4$	32/ 18
3	$TBABF_4$	---
4	TBAI	24/ 11
5	$TBAPF_6$	45/ 22

Table S3. Screening for solvent.

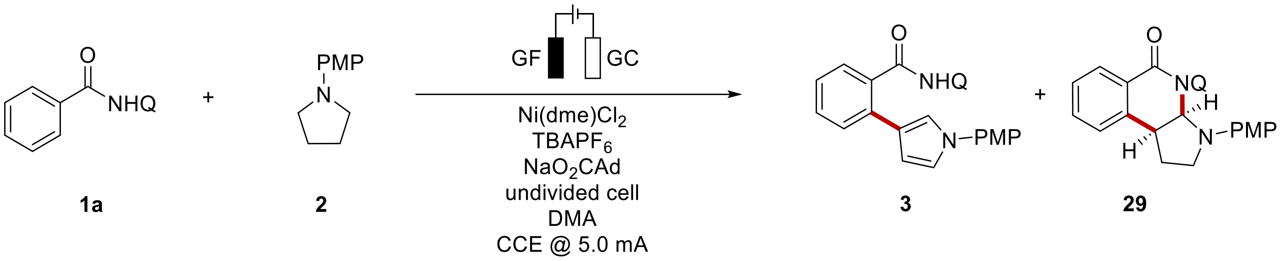

Entry	Deviation of solvent	Yield% (**3**/**29**)[a]
1	none	45/ 22
2	DMF	---/ 15
3	DMSO	---/ 6
4	HMPA	23/ 5
5	$MeNO_2$	---

Table S4. Further Optimizations.

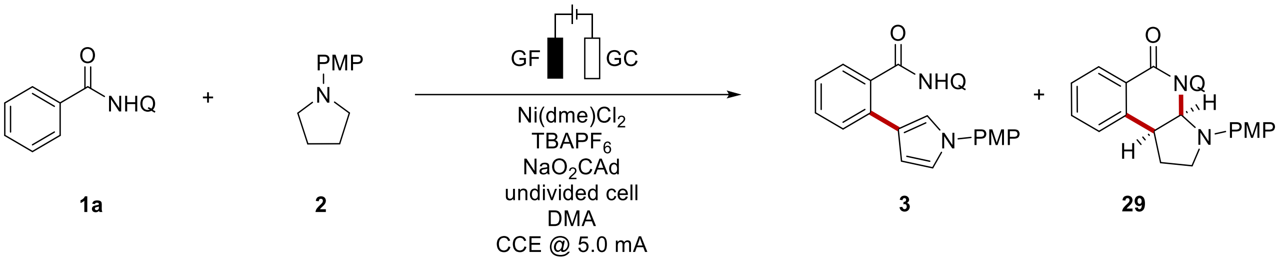

Entry	Deviation	Yield% (**3**/**29**)[a]
1	none	45/ 22
2	8.0 mA	64 /5
3	4.0 mA	16/24
4	8 h/24 h	32/40
5	0.15 mmol scale **1a**	62
6	CCE @ 4.0 mA/10.0 mA	16/26
7	120 °C	---
8	160 °C	67
9	3.0 equiv./4.0 equiv. of **2**	68/42
10	$Ni(dme)Br_2$/$NiCl_2$ as [Ni]	19/---
11	No NaO_2CAd	---
12	No [Ni], no electricity	---/---
13	NaO*t*Bu/NaO_2CPh/Na_2CO_3	---/10/---

[a] Reaction conditions: **1a** (0.25 mmol), **2** (0.50 mmol), $Ni(dme)Cl_2$ (10 mol %), $TBAPF_6$ (1.6 equiv.), NaO_2CAd (2.0 equiv.), DMA (4.0 mL), CCE @ 8.0 mA, 140 °C, 17 h, undivided cell with Glassy Carbon (GC) cathode (10 mm × 25 mm × 0.25 mm), Graphite Felt (GF) anode (10 mm × 15 mm × 6 mm), under N_2. Yields were determined by ^{1}H-NMR using benzaldehyde as internal standard. Isolated yield is given in parentheses. DMA=*N*, *N*-dimethylacetamide, PMP= *para*-methoxyphenyl, dme=ethylene glycol dimethyl ether, TBA=tetra-*n*-butylammonium, Q=8-quinoline.

III. General Procedures

General Procedure A: Nickela-electrocatalyzed Synthesis of *β*-Arylated Pyrroles.

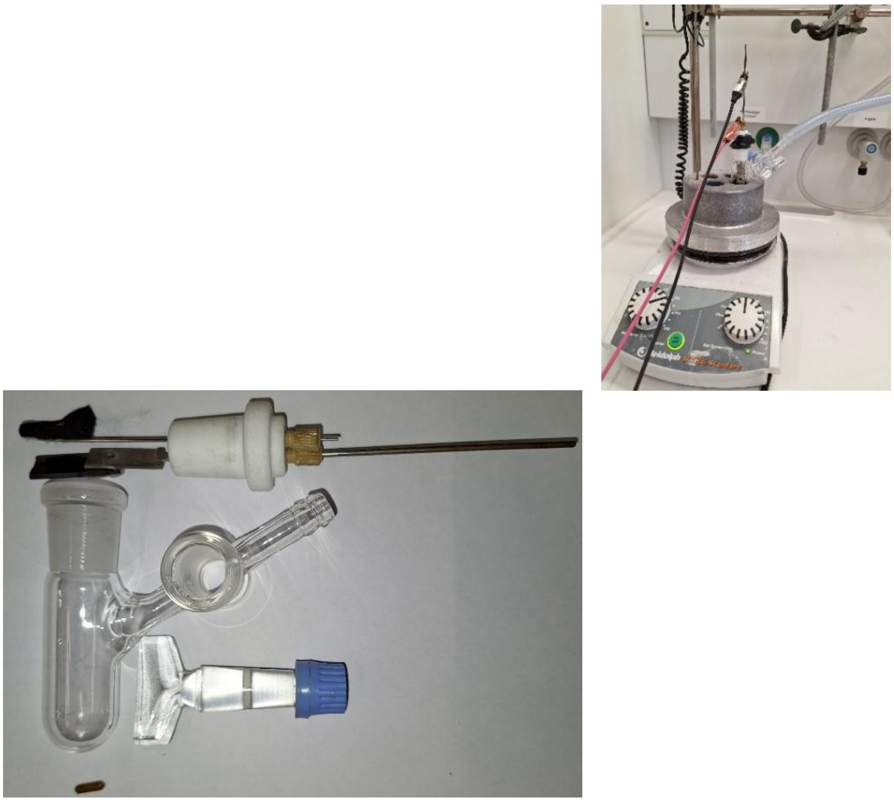

Figure S2. Photo of the reaction set-up.

The electrolysis was carried out in an undivided cell with a graphite felt anode (10 mm × 15 mm × 6 mm) and a glassy carbon cathode (10 mm × 25 mm × 0.25 mm). Ni(dme)Cl_2 (5.5 mg, 0.025 mmol, 10 mol %), AdCO_2Na (101 mg, 0.5 mmol, 2.0 equiv.), *n*-Bu_4NPF_6 (155 mg, 0.4 mmol, 0.1 M, 1.6 equiv.), benzamide **1** (0.25 mmol, 1.0 equiv.) and *N*-aryl amine **2** (0.5 mmol, 2.0 equiv.) were dissolved in DMA (4.0 mL) and the electrolysis was performed at 140 °C with a constant current (8 mA) for 17 h under N_2. At ambient temperature, the mixture was transferred to a separating funnel and the electrodes and flask were rinsed with EtOAc (2 × 10 mL). Then, the mixture was washed with sat. aq. $NaHCO_3$ (50 mL) and extracted with EtOAc (2 × 20 mL). The combined organic layers were washed with brine (50 mL) and dried over anhydrous Na_2SO_4. The filtrate was concentrated *in vacuo* and subsequent column chromatography on silica gel (*n*-hexane/ethyl acetate) yielded the desired product.

IV. Characterization Data of Products

2-(1-(4-Methoxyphenyl)-1*H*-pyrrol-3-yl)-*N*-(quinolin-8-yl)benzamide (**3**):

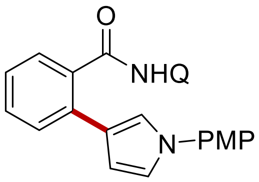

General Procedure **A** was followed using benzamide **1a** (62.0 mg). Column chromatography on silica gel (*n*-hexane/ethyl acetate = 4:1) yielded **3** (64.0 mg, 61%) as light brown solid.

M. p.: 131–133 °C.

^{1}H-NMR (400 MHz, $CDCl_3$) δ = 10.12 (s, 1H), 8.96 (dd, J = 7.6, 1.3 Hz, 1H), 8.48 (dd, J = 4.2, 1.7 Hz, 1H), 8.07 (dd, J = 8.3, 1.7 Hz, 1H), 7.83 (dd, J = 7.7, 1.4 Hz, 1H), 7.58 (ddd, J = 7.8, 4.8, 3.4 Hz, 2H), 7.54–7.46 (m, 2H), 7.38 (td, J = 7.5, 1.3 Hz, 1H), 7.29 (dd, J = 8.3, 4.2 Hz, 1H), 7.24 (t, J = 2.0 Hz, 1H), 7.09–7.00 (m, 2H), 6.92–6.79 (m, 3H), 6.49 (dd, J = 2.9, 1.8 Hz, 1H), 3.79 (s, 3H).

^{13}C-NMR (101 MHz, $CDCl_3$) δ = 168.9 (C_q), 157.7 (C_q), 148.0 (CH), 138.7 (C_q), 136.1 (CH), 135.5 (C_q), 135.1 (C_q), 134.1 (C_q), 134.0 (C_q), 130.4 (CH), 129.8 (CH), 129.0 (CH), 128.0 (C_q), 127.5 (CH), 126.5 (CH), 124.3 (C_q), 121.9 (CH), 121.6 (CH), 121.5 (CH), 120.3 (CH), 118.6 (CH), 116.5 (CH), 114.6 (CH), 110.8 (CH), 55.6 (CH_3).

IR (ATR): 3345, 1668, 1597, 1518, 1485, 1442, 1424, 1385, 1326, 1248 cm^{-1}.

HR-MS (ESI): *m/z* calcd. for $C_{27}H_{22}N_3O_2$ $[M+H]^+$ 420.1707, found 420.1717.

^{1}H-NMR (400 MHz, $CDCl_3$) δ = 10.12 (s, 1H, N$\underline{H}$), 8.96 (dd, J = 7.6, 1.3 Hz, 1H, Ar of quinoline), 8.48 (dd, J = 4.2, 1.7 Hz, 1H, Ar of quinoline), 8.07 (dd, J = 8.3, 1.7 Hz, 1H, Ar of quinoline), 7.83 (dd, J = 7.7, 1.4 Hz, 1H, Ar of quinoline), 7.58 (ddd, J = 7.8, 4.8, 3.4 Hz, 2H, Ar of benzoyl), 7.54–7.46 (m, 2H, Ar of benzoyl), 7.38 (td, J = 7.5, 1.3 Hz, 1H, Ar of quinoline), 7.29 (dd, J = 8.3, 4.2 Hz, 1H, Ar of quinoline), 7.24 (t, J = 2.0 Hz, 1H, Ar of pyrrole), 7.09–7.00 (m, 2H, Ar of PMP), 6.92–6.79 (m, 3H, Ar of PMP and pyrrole), 6.49 (dd, J = 2.9, 1.8 Hz, 1H, Ar of pyrrole), 3.79 (s, 3H, OC$\underline{H_3}$).

2-(1-(4-Methoxyphenyl)-1*H*-pyrrol-3-yl)-6-methyl-*N*-(quinolin-8-yl)benzamide (**4**):

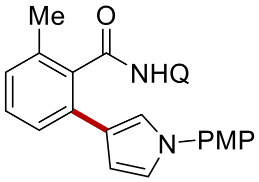

General Procedure **A** was followed using Ni(dme)Cl_2 (20 mol%) and benzamide **1b** (65.6 mg). Column chromatography on silica gel (*n*-hexane/ethyl acetate = 4:1) yielded **4** (47.7 mg, 44%) as colourless oil.

^{1}H-NMR (400 MHz, $CDCl_3$) δ = 9.88 (bs, 1H), 8.97 (dd, J = 7.6, 1.4 Hz, 1H), 8.63 (dd, J = 4.2, 1.7 Hz, 1H), 8.12 (dd, J = 8.3, 1.7 Hz, 1H), 7.60 (t, J = 7.9 Hz, 1H), 7.52 (dd, J = 8.3, 1.4 Hz, 1H), 7.45–7.40 (m, 1H), 7.39–7.33 (m, 2H), 7.23 (t, J = 2.0 Hz, 1H), 7.20–7.15 (m, 1H), 7.03–6.97 (m, 2H), 6.82–6.76 (m, 3H), 6.52 (dd, J = 2.9, 1.8 Hz, 1H), 3.78 (s, 3H), 2.50 (s, 3H).

^{13}C-NMR (101 MHz, $CDCl_3$) δ = 169.8 (C_q), 157.7 (C_q), 148.3 (CH), 138.7 (C_q), 136.3 (CH), 136.0 (C_q), 135.6 (C_q), 135.0 (C_q), 134.2 (C_q), 133.5 (C_q), 129.3 (CH), 128.1 (CH), 128.1 (C_q), 127.5 (CH),

126.5 (CH), 124.5 (C_q), 122.0 (CH), 121.9 (CH), 121.7 (CH), 120.1 (CH), 118.3 (CH), 116.8 (CH), 114.6 (CH), 110.4 (CH), 55.7 (CH_3), 19.8 (CH_3).
IR (ATR): 3348, 2904, 1724, 1672, 1517, 1482, 1247, 1031, 750, 608 cm^{-1}.
HR-MS (ESI): *m/z* calcd for $C_{28}H_{24}N_3O_2$ $[M+H]^+$ 434.1863; found: 434.1863.

2-(1-(4-Methoxyphenyl)-1*H*-pyrrol-3-yl)-5-methyl-*N*-(quinolin-8-yl)benzamide (**5**):

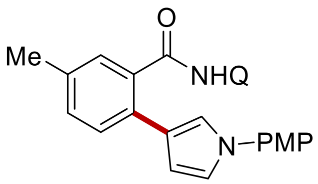

General Procedure **A** was followed using benzamide **1c** (65.5 mg). Column chromatography on silica gel (*n*-hexane/ethyl acetate = 4:1) yielded **5** (84.5 mg, 78%) as colourless solid.
M. p.: 160–161 °C.
^{1}H-NMR (300 MHz, $CDCl_3$) δ = 10.10 (s, 1H), 8.95 (dd, J = 7.6, 1.4 Hz, 1H), 8.47 (dd, J = 4.2, 1.7 Hz, 1H), 8.07 (dd, J = 8.3, 1.7 Hz, 1H), 7.64 (d, J = 1.8 Hz, 1H), 7.58 (t, J = 7.9 Hz, 1H), 7.53–7.42 (m, 2H), 7.29 (dd, J = 8.3, 4.2 Hz, 2H), 7.20 (t, J = 2.0 Hz, 1H), 7.04–6.98 (m, 2H), 6.86–6.76 (m, 3H), 6.45 (dd, J = 2.9, 1.8 Hz, 1H), 3.79 (s, 3H), 2.44 (s, 3H).
^{13}C-NMR (75 MHz, $CDCl_3$) δ = 169.1 (C_q), 157.7 (C_q), 148.0 (CH), 138.8 (C_q), 136.3 (CH), 136.1 (C_q), 135.3 (C_q), 135.1 (C_q), 134.2 (C_q), 131.2 (C_q), 131.2 (CH), 129.8 (CH), 129.6 (CH), 128.0 (C_q), 127.5 (CH), 124.2 (C_q), 121.9 (CH), 121.5 (CH), 120.2 (CH), 118.4 (CH), 116.6 (CH), 114.6 (CH), 110.9 (CH), 55.7 (CH_3), 21.1 (CH_3).
IR (ATR): 3329, 1668, 1597, 1519, 1484, 1424, 1384, 1356, 1326, 1248 cm^{-1}.
HR-MS (ESI): *m/z* calcd. for $C_{28}H_{24}N_3O_2$ $[M+H]^+$ 434.1863, found 434.1868.

2-(1-(4-Methoxyphenyl)-1*H*-pyrrol-3-yl)-4-methyl-*N*-(quinolin-8-yl)benzamide (**6**):

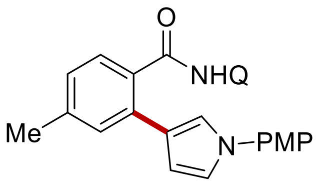

General Procedure **A** was followed using benzamide **1d** (65.6 mg). Column chromatography on silica gel (*n*-hexane/ethyl acetate = 4:1) yielded **6** (78.0 mg, 72%) as brown solid.
M. p.: 166–168 °C.
^{1}H-NMR (400 MHz, $CDCl_3$) δ = 10.12 (s, 1H), 8.95 (dd, J = 7.7, 1.3 Hz, 1H), 8.45 (dd, J = 4.2, 1.7 Hz, 1H), 8.06 (dd, J = 8.3, 1.7 Hz, 1H), 7.76 (d, J = 7.8 Hz, 1H), 7.57 (t, J = 7.9 Hz, 1H), 7.47 (dd, J = 8.2, 1.4 Hz, 1H), 7.38 (d, J = 1.8 Hz, 1H), 7.30–7.27 (m, 1H), 7.25–7.18 (m, 2H), 7.04 (d, J = 8.9 Hz, 2H), 6.86–6.78 (m, 3H), 6.46 (dd, J = 2.9, 1.8 Hz, 1H), 3.80 (s, 3H), 2.45 (s, 3H).
^{13}C-NMR (101 MHz, $CDCl_3$) δ = 168.9 (C_q), 157.7 (C_q), 148.0 (CH), 140.6 (C_q), 138.7 (C_q), 136.1 (CH), 135.2 (C_q), 134.2 (C_q), 134.1 (C_q), 132.8 (C_q), 130.6 (CH), 129.3 (CH), 128.0 (C_q), 127.5 (CH), 127.3 (CH), 124.4 (C_q), 121.9 (CH), 121.4 (CH), 120.3 (CH), 118.6 (CH), 116.4 (CH), 114.6 (CH), 111.0 (CH), 55.6 (CH_3), 21.5 (CH_3).
IR (ATR): 3307, 3046, 2922, 1711, 1665, 1517, 1482, 1424, 1385, 1247 cm^{-1}.
HR-MS (ESI): *m/z* calcd for $C_{28}H_{24}N_3O_2$ $[M+H]^+$ 434.1863, found 434.1868.

3-(1-(4-Methoxyphenyl)-1*H*-pyrrol-3-yl)-*N*-(quinolin-8-yl)-[1, 1'-biphenyl]-3-carboxamide (**7**):

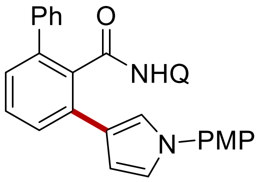

General Procedure **A** was followed using benzamide **1e** (81.1 mg). Column chromatography on silica gel (*n*-hexane/ethyl acetate = 4:1) yielded **7** (69.4 mg, 56%) as light brown oil.

^{1}H-NMR (400 MHz, $CDCl_3$) δ = 9.78 (s, 1H), 8.71 (dd, J = 7.4, 1.6 Hz, 1H), 8.58 (dd, J = 4.2, 1.7 Hz, 1H), 8.06 (dd, J = 8.3, 1.7 Hz, 1H), 7.61–7.55 (m, 3H), 7.51 (t, J = 7.7 Hz, 1H), 7.49–7.42 (m, 2H), 7.35–7.30 (m, 2H), 7.29–7.26 (m, 2H), 7.24 (d, J = 1.6 Hz, 1H), 7.20–7.14 (m, 1H), 7.05–6.99 (m, 2H), 6.83–6.77 (m, 3H), 6.54 (dd, J = 2.9, 1.8 Hz, 1H), 3.78 (s, 3H).

^{13}C-NMR (101 MHz, $CDCl_3$) δ = 168.8 (C_q), 157.7 (C_q), 148.1 (CH), 140.7 (C_q), 140.6 (C_q), 138.6 (C_q), 136.1 (CH), 135.2 (C_q), 134.8 (C_q), 134.2 (C_q), 129.4 (CH), 129.0 (CH), 128.4 (CH), 128.2 (CH), 127.9 (C_q), 127.4 (CH), 124.4 (C_q), 122.0 (CH), 121.6 (CH), 121.5 (CH), 120.1 (CH), 118.6 (CH), 116.7 (CH), 114.6 (CH), 110.7 (CH), 55.7 (CH_3).

IR (ATR): 3341, 3056, 2933, 1711, 1673, 1515, 1482, 1460, 1326, 1247 cm^{-1}.

HR-MS (ESI): *m/z* calcd for $C_{33}H_{26}N_3O_2$ $[M+H]^+$ 496.2020, found 496.2019.

4-(1-(4-Methoxyphenyl)-1*H*-pyrrol-3-yl)-*N*-(quinolin-8-yl)-[1, 1'-biphenyl]-3-carboxamide (**8**):

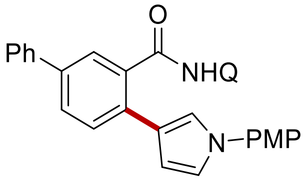

General Procedure **A** was followed using benzamide **1f** (81.1 mg). Column chromatography on silica gel (*n*-hexane/ethyl acetate = 4:1) yielded **8** (105.3 mg, 85%) as colourless solid.

M. p.: 195–197 °C.

^{1}H-NMR (300 MHz, $CDCl_3$) δ = 10.17 (s, 1H), 8.97 (dd, J = 7.6, 1.4 Hz, 1H), 8.50 (dd, J = 4.2, 1.7 Hz, 1H), 8.09 (dd, J = 8.3, 1.7 Hz, 1H), 8.06 (d, J = 2.0 Hz, 1H), 7.79–7.68 (m, 2H), 7.70–7.62 (m, 2H), 7.58 (d, J = 7.8 Hz, 1H), 7.54–7.43 (m, 3H), 7.40–7.35 (m, 1H), 7.31 (dd, J = 8.3, 4.2 Hz, 1H), 7.28 (t, J = 2.0 Hz, 1H), 7.11–6.99 (m, 2H), 6.86–6.78 (m, 3H), 6.52 (dd, J = 2.9, 1.8 Hz, 1H), 3.80 (s, 3H).

^{13}C-NMR (75 MHz, $CDCl_3$) δ = 169.0 (C_q), 157.8 (C_q), 148.1 (CH), 140.3 (C_q), 139.3 (C_q), 138.7 (C_q), 136.2 (CH), 135.9 (C_q), 135.1 (C_q), 134.2 (C_q), 133.0 (C_q), 130.3 (CH), 129.0 (CH), 128.0 (C_q), 127.6 (CH), 127.5 (CH), 127.1 (CH), 123.9 (C_q), 122.0 (CH), 121.7 (CH), 121.6 (CH), 120.5 (CH), 118.7 (CH), 116.6 (CH), 114.6 (CH), 110.9 (CH), 55.7 (CH_3).

IR (ATR): 2905, 2851, 1695, 1675, 1517, 1485, 1453, 1327, 1281, 1247 cm^{-1}.

HR-MS (ESI): *m/z* calcd. for $C_{33}H_{26}N_3O_2$ $[M+H]^+$ 496.2020, found 496.2025.

3-(1-(4-Methoxyphenyl)-1*H*-pyrrol-3-yl)-*N*-(quinolin-8-yl)-[1, 1'-biphenyl]-4-carboxamide (**9**):

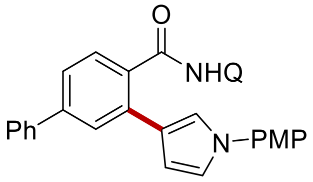

General Procedure **A** was followed using benzamide **1g** (81.1 mg). Column chromatography on silica gel (*n*-hexane/ethyl acetate = 4:1) yielded **9** (50.8 mg, 41%) as light brown oil.

^{1}H-NMR (400 MHz, $CDCl_3$) δ = 10.19 (s, 1H), 8.97 (dd, J = 7.6, 1.3 Hz, 1H), 8.47 (dd, J = 4.1, 1.7 Hz, 1H), 8.09 (dd, J = 8.3, 1.7 Hz, 1H), 7.93 (d, J = 8.0 Hz, 1H), 7.79 (d, J = 1.9 Hz, 2H), 7.73–7.66 (m, 2H), 7.64–7.56 (m, 3H), 7.46–7.37 (m, 1H), 7.35–7.28 (m, 2H), 7.26 (s, 1H), 7.14–7.03 (m, 2H), 6.92–6.77 (m, 3H), 6.52 (dd, J = 2.9, 1.8 Hz, 1H), 3.80 (s, 3H).

^{13}C-NMR (101 MHz, $CDCl_3$) δ = 168.7 (C_q), 157.8 (C_q), 148.1 (CH), 143.4 (C_q), 140.6 (C_q), 138.8 (C_q), 136.1 (CH), 135.1 (C_q), 134.6 (C_q), 134.3 (C_q), 134.2 (C_q), 129.9 (CH), 129.0 (CH), 128.8 (CH), 128.0 (C_q), 127.9 (CH), 127.6 (CH), 127.4 (CH), 125.3 (CH), 124.3 (C_q), 122.0 (CH), 121.6 (CH), 121.5 (CH), 120.5 (CH), 118.8 (CH), 116.6 (CH), 114.7 (CH), 111.0 (CH), 55.7 (CH_3).

IR (ATR): 2657, 2931, 1666, 1599, 1518, 1485, 1424, 1385, 1326, 1248 cm^{-1}.

HR-MS (ESI): *m/z* calcd for $C_{33}H_{26}N_3O_2$ $[M+H]^+$ 496.2020, found 496.2015.

4-Isopropyl-2-(1-(4-methoxyphenyl)-1*H*-pyrrol-3-yl)-*N*-(quinolin-8-yl)benzamide (**10**):

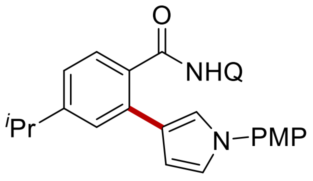

General Procedure **A** was followed using benzamide **1h** (72.6 mg). Column chromatography on silica gel (*n*-hexane/ethyl acetate = 4:1) yielded **10** (100.4 mg, 87%) brown solid.

M. p.: 144–145 °C.

^{1}H-NMR (400 MHz, $CDCl_3$) δ = 10.12 (s, 1H), 8.95 (dd, J = 7.7, 1.3 Hz, 1H), 8.45 (dd, J = 4.2, 1.7 Hz, 1H), 8.07 (dd, J = 8.3, 1.7 Hz, 1H), 7.79 (d, J = 7.9 Hz, 1H), 7.57 (t, J = 8.0 Hz, 1H), 7.48 (d, J = 1.3 Hz, 1H), 7.41 (d, J = 1.8 Hz, 1H), 7.32–7.25 (m, 2H), 7.24 (q, J = 2.3 Hz, 1H), 7.13–6.97 (m, 2H), 6.86–6.69 (m, 3H), 6.48 (dd, J = 2.9, 1.8 Hz, 1H), 3.80 (s, 3H), 3.01 (sept, J = 6.9 Hz, 1H), 1.33 (d, J = 6.9 Hz, 6H).

^{13}C-NMR (101 MHz, $CDCl_3$) δ = 168.9 (C_q), 157.7 (C_q), 151.5 (C_q), 148.0 (CH), 138.8 (C_q), 136.1 (CH), 135.2 (C_q), 134.2 (C_q), 134.1 (C_q), 133.2 (C_q), 129.4 (CH), 128.2 (CH), 128.0 (C_q), 127.5 (CH), 124.8 (CH), 124.7 (C_q), 121.9 (CH), 121.5 (CH), 120.3 (CH), 118.6 (CH), 116.5 (CH), 114.6 (CH), 111.0 (CH), 55.7 (CH_3), 34.3 (CH), 24.0 (CH_3).

IR (ATR): 2959, 1711, 1664, 1514, 1481, 1423, 1383, 1325, 1546, 1220 cm^{-1}.

HR-MS (ESI): *m/z* calcd for $C_{30}H_{28}N_3O_2$ $[M+H]^+$462.2176, found 462.2176.

4-(*tert*-Butyl)-2-(1-(4-methoxyphenyl)-1*H*-pyrrol-3-yl)-*N*-(quinolin-8-yl)benzamide (11):

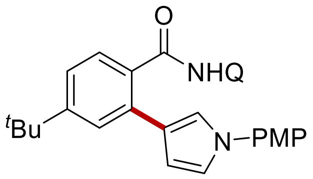

General Procedure **A** was followed using benzamide **1i** (76.1 mg). Column chromatography on silica gel (*n*-hexane/ethyl acetate = 4:1) yielded **11** (104.6 mg, 88%) as brown oil.

^{1}H-NMR (400 MHz, $CDCl_3$) δ = 10.13 (s, 1H), 8.96 (dd, J = 7.7, 1.3 Hz, 1H), 8.45 (dd, J = 4.2, 1.7 Hz, 1H), 8.06 (dd, J = 8.3, 1.7 Hz, 1H), 7.82 (d, J = 8.2 Hz, 1H), 7.61–7.52 (m, 2H), 7.47 (dd, J = 8.3, 1.3 Hz, 1H), 7.44 (dd, J = 8.2, 2.0 Hz, 1H), 7.31–7.26 (m, 1H), 7.25 (t, J = 2.0 Hz, 1H), 7.09–7.02 (m, 2H), 6.87–6.78 (m, 3H), 6.49 (dd, J = 2.9, 1.8 Hz, 1H), 3.80 (s, 3H), 1.41 (s, 9H).

^{13}C-NMR (101 MHz, $CDCl_3$) δ =168.9 (C_q), 157.7 (C_q), 153.7 (C_q), 147.9 (CH), 138.7 (C_q), 136.1 (CH), 135.2 (C_q), 134.2 (C_q), 133.8 (C_q), 132.9 (C_q), 129.1 (CH), 128.0 (C_q), 127.5 (CH), 127.0 (CH), 124.9 (C_q), 123.8 (CH), 121.9 (CH), 121.4 (CH), 120.3 (CH), 118.6 (CH), 116.5 (CH), 114.6 (CH), 111.1 (CH), 55.6 (CH_3), 35.0 (C_q), 31.4 (CH_3).

IR (ATR): 3341, 3310, 2961, 2906, 1664, 1515, 1462, 1684, 1326, 1247 cm^{-1}.

HR-MS (ESI): *m/z* calcd for $C_{31}H_{29}N_3O_2Na$ $[M+Na]^+$ 498.2152, found 498.2150.

4-Cyclohexyl-2-(1-(4-methoxyphenyl)-1*H*-pyrrol-3-yl)-*N*-(quinolin-8-yl)benzamide (12):

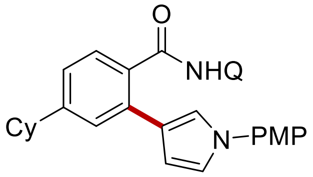

General Procedure **A** was followed using benzamide **1j** (82.6 mg). Column chromatography on silica gel (*n*-hexane/ethyl acetate = 4:1) yielded **12** (57.7 mg, 46%) as brown oil.

^{1}H-NMR (400 MHz, $CDCl_3$) δ = 10.12 (s, 1H), 8.95 (dd, J = 7.6, 1.4 Hz, 1H), 8.45 (dd, J = 4.2, 1.7 Hz, 1H), 8.07 (dd, J = 8.3, 1.7 Hz, 1H), 7.78 (d, J = 7.9 Hz, 1H), 7.57 (t, J = 8.0 Hz, 1H), 7.47 (dd, J = 8.2, 1.3 Hz, 1H), 7.40 (s, 1H), 7.28 (dd, J = 8.3, 4.2 Hz, 1H), 7.26–7.22 (m, 2H), 7.08–7.01 (m, 2H), 6.86–6.77 (m, 3H), 6.47 (dd, J = 2.9, 1.7 Hz, 1H), 3.80 (s, 3H), 2.61 (tt, J = 11.7, 3.3 Hz, 1H), 1.99–1.85 (m, 4H), 1.83–1.71 (m, 1H), 1.57–1.40 (m, 4H), 1.30 (ddt, J = 16.2, 12.8, 6.1 Hz, 1H).

^{13}C-NMR (101 MHz, $CDCl_3$) δ = 168.9 (C_q), 157.7 (C_q), 150.7 (C_q), 148.0 (CH), 138.8 (C_q), 136.1 (CH), 135.2 (C_q), 134.2 (C_q), 134.1 (C_q), 133.2 (C_q), 129.4 (CH), 128.6 (CH), 128.0 (C_q), 127.5 (CH), 125.2 (CH), 124.7 (C_q), 121.9 (CH), 121.4 (CH), 120.3 (CH), 118.6 (CH), 116.5 (CH), 114.6 (CH), 111.0 (CH), 55.7 (CH_3), 44.7 (CH), 34.4 (CH_2), 27.0 (CH_2), 26.3 (CH_2).

IR (ATR): 3331, 2925, 2850, 1712, 1667, 1604, 1517, 1483, 1326, 1248 cm^{-1}.

HR-MS (ESI): *m/z* calcd for $C_{33}H_{32}N_3O_2$ $[M+H]^+$ 502.2489, found 502.2484.

2-(1-(4-Methoxyphenyl)-1*H*-pyrrol-3-yl)-4-phenoxy-*N*-(quinolin-8-yl)benzamide (**13**):

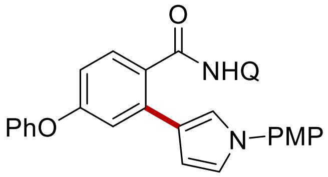

General Procedure **A** was followed using benzamide **1h** (85.1 mg). Column chromatography on silica gel (*n*-hexane/ethyl acetate = 4:1) yielded **13** (87.0 mg, 68%) as light brown oil.

^{1}H-NMR (400 MHz, $CDCl_3$) δ = 10.12 (s, 1H), 8.94 (dd, J = 7.7, 1.4 Hz, 1H), 8.46 (dd, J = 4.2, 1.7 Hz, 1H), 8.08 (dd, J = 8.2, 1.7 Hz, 1H), 7.83 (d, J = 8.4 Hz, 1H), 7.58 (t, J = 7.9 Hz, 1H), 7.49 (dd, J = 8.3, 1.4 Hz, 1H), 7.44–7.36 (m, 2H), 7.29 (dd, J = 8.3, 4.2 Hz, 1H), 7.20 (t, J = 2.0 Hz, 1H), 7.19–7.16 (m, 2H), 7.15–7.11 (m, 2H), 7.01–6.96 (m, 3H), 6.86–6.75 (m, 3H), 6.43 (dd, J = 2.9, 1.8 Hz, 1H), 3.79 (s, 3H).

^{13}C-NMR (101 MHz, $CDCl_3$) δ = 168.3 (C_q), 159.2 (C_q), 157.8 (C_q), 156.6 (C_q), 148.0 (CH), 138.7 (C_q), 136.4 (C_q), 136.1 (CH), 135.1 (C_q), 134.1 (C_q), 131.3 (CH), 130.3 (C_q), 130.1 (CH), 128.0 (C_q), 127.5 (CH), 124.0 (CH), 123.9 (C_q), 122.0 (CH), 121.5 (CH), 120.5 (CH), 119.7 (CH), 119.3 (CH), 118.8 (CH), 116.5 (CH), 116.3 (CH), 114.6 (CH), 110.9 (CH), 55.7 (CH_3).

IR (ATR): 3310, 2932, 2835, 1664, 1588, 1517, 1480, 1326, 1248, 1219 cm^{-1}.

HR-MS (ESI) *m/z* calcd for $C_{33}H_{26}N_3O_3$ $[M+H]^+$ 512.1969, found 512.1966.

4-(Benzyloxy)-2-(1-(4-methoxyphenyl)-1*H*-pyrrol-3-yl)-*N*-(quinolin-8-yl)benzamide (**14**):

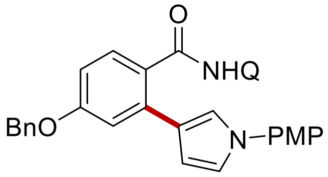

General Procedure **A** was followed using benzamide **1l** (88.6 mg). Column chromatography on silica gel (*n*-hexane/ethyl acetate = 4:1) yielded **14** (52.5 mg, 40%) as light brown oil.

^{1}H-NMR (400 MHz, $CDCl_3$) δ = 10.12 (s, 1H), 8.95 (dd, J = 7.7, 1.4 Hz, 1H), 8.42 (dd, J = 4.2, 1.7 Hz, 1H), 8.06 (dd, J = 8.3, 1.7 Hz, 1H), 7.85 (d, J = 8.6 Hz, 1H), 7.56 (t, J = 7.9 Hz, 1H), 7.51–7.45 (m, 3H), 7.44–7.39 (m, 2H), 7.39–7.32 (m, 1H), 7.29–7.26 (m, 1H), 7.21 (t, J = 2.0 Hz, 1H), 7.15 (d, J = 2.6 Hz, 1H), 7.04–6.97 (m, 3H), 6.87–6.67 (m, 3H), 6.45 (dd, J = 2.9, 1.8 Hz, 1H), 5.18 (s, 2H), 3.80 (s, 3H).

^{13}C-NMR (101 MHz, $CDCl_3$) δ = 168.4 (C_q), 160.4 (C_q), 157.8 (C_q), 147.9 (CH), 138.7 (C_q), 136.8 (C_q), 136.2 (C_q), 136.1 (CH), 135.2 (C_q), 134.1 (C_q), 131.4 (CH), 128.8 (CH), 128.4 (C_q), 128.2 (CH), 128.0 (C_q), 127.7 (CH), 127.5 (CH), 124.3 (C_q), 121.9 (CH), 121.4 (CH), 120.4 (CH), 118.8 (CH), 116.4 (CH), 116.1 (CH), 114.6 (CH), 112.9 (CH), 111.0 (CH), 70.2 (CH_2), 55.7 (CH_3).

IR (ATR): 3309, 2932, 1710, 1660, 1597, 1515, 1480, 1246, 1219, 1201 cm^{-1}.

HR-MS (ESI): *m/z* calcd for $C_{34}H_{28}N_3O_3$ $[M+H]^+$ 526.2125, found 526.2122.

2-(1-(4-Methoxyphenyl)-1*H*-pyrrol-3-yl)-*N*-(quinolin-8-yl)-4-(trifluoromethoxy)benzamide (15):

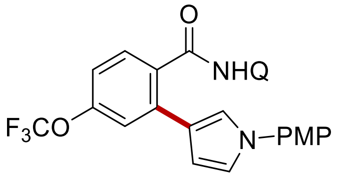

General Procedure **A** was followed using benzamide **1m** (83.0 mg). Column chromatography on silica gel (*n*-hexane/ethyl acetate = 4:1) yielded **15** (82.0 mg, 60%) as brown oil.

^{1}H-NMR (400 MHz, $CDCl_3$) δ = 10.11 (s, 1H), 8.92 (dd, J = 7.6, 1.4 Hz, 1H), 8.49 (dd, J = 4.2, 1.7 Hz, 1H), 8.10 (dd, J = 8.3, 1.7 Hz, 1H), 7.85 (d, J = 8.4 Hz, 1H), 7.59 (t, J = 8.0 Hz, 1H), 7.51 (dd, J = 8.3, 1.4 Hz, 1H), 7.40 (dt, J = 2.1, 1.1 Hz, 1H), 7.32 (dd, J = 8.3, 4.2 Hz, 1H), 7.24 (t, J = 2.0 Hz, 1H), 7.22 (ddt, J = 7.4, 2.5, 1.1 Hz, 1H), 7.06–6.97 (m, 2H), 6.86–6.79 (m, 3H), 6.47 (dd, J = 2.9, 1.8 Hz, 1H), 3.80 (s, 3H).

^{13}C-NMR (101 MHz, $CDCl_3$) δ = 167.8 (C_q), 158.0 (C_q), 150.6 (q, $^3J_{C–F}$ = 1.7 Hz, C_q), 148.2 (CH), 138.7 (q, $^1J_{C–F}$ = 390.1 Hz, C_q), 136.4 (C_q), 136.2 (CH), 134.8 (C_q), 133.9 (q, $^4J_{C–F}$ = 8.7 Hz, CH), 130.9 (CH), 128.0 (C_q), 127.5 (CH), 123.1 (C_q), 122.1 (CH), 121.9 (CH), 121.7 (CH), 121.6 (CH), 120.8 (q, $^4J_{C–F}$ = 81.7 Hz, CH), 119.0 (CH), 118.5 (CH), 116.7 (CH), 114.7 (CH), 110.7 (CH), 55.7 (CH_3).

^{19}F-NMR (377 MHz, $CDCl_3$) δ = –57.5.

IR (ATR): 2959, 2911, 2583, 1675, 1519, 1485, 1327, 1252, 1220, 1179 cm^{-1}.

HR-MS (ESI): *m/z* calcd for $C_{28}H_{21}F_3N_3O_3$ $[M+H]^+$ 504.1530, found 504.1528.

2-(1-(4-Methoxyphenyl)-1*H*-pyrrol-3-yl)-*N*-(quinolin-8-yl)-6-(trifluoromethyl)benzamide (**16**):

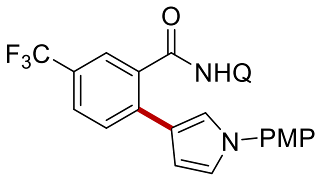

General Procedure **A** was followed using benzamide **1n** (79.1 mg). Column chromatography on silica gel (*n*-hexane/ethyl acetate = 4:1) yielded **16** (30.5 mg, 25%) as brown oil.

^{1}H-NMR (400 MHz, $CDCl_3$) δ = 10.13 (s, 1H), 8.93 (dd, J = 7.6, 1.4 Hz, 1H), 8.50 (dd, J = 4.2, 1.7 Hz, 1H), 8.15–8.04 (m, 2H), 7.76–7.66 (m, 2H), 7.60 (t, J = 8.0 Hz, 1H), 7.53 (dd, J = 8.3, 1.4 Hz, 1H), 7.33 (dd, J = 8.3, 4.2 Hz, 1H), 7.27 (t, J = 2.1 Hz, 1H), 7.02 (d, J = 6.7 Hz, 2H), 6.86–6.79 (m, 3H), 6.50 (dd, J = 2.9, 1.8 Hz, 1H), 3.80 (s, 3H).

^{13}C-NMR (101 MHz, $CDCl_3$) δ = 167.6 (C_q), 158.1 (C_q), 148.2 (CH), 138.7 (C_q), 137.6 (C_q), 136.3 (CH), 135.6 (q, $^2J_{C–F}$ = 88.2 Hz, C_q), 134.7 (C_q), 133.9 (q, $^1J_{C–F}$ = 480.9 Hz, C_q), 130.1 (CH), 128.0 (q, $^3J_{C–F}$ = 34.5 Hz, CH), 127.5 (CH), 127.0 (q, $^4J_{C–F}$ = 3.6 Hz, CH), 127.0 (CH), 126.3 (q, $^4J_{C–F}$ = 4.1 Hz, C_q), 123.0 (C_q), 122.1 (q, $^3J_{C–F}$ = 38.7 Hz, CH), 121.7 (CH), 121.0 (CH), 119.3 (CH), 116.8 (CH), 114.7 (CH), 110.7 (CH), 55.7 (CH_3).

^{19}F-NMR (377 MHz, $CDCl_3$) δ = –62.4.

IR (ATR): 2957, 2925, 2839, 1711, 1668, 1514, 1323, 1246, 1167, 1092 cm^{-1}.

HR-MS (ESI): *m/z* calcd for $C_{28}H_{21}N_3O_2F_3$ $[M+H]^+$ 488.1580, found 488.1588.

2-(1-(4-Methoxyphenyl)-1*H*-pyrrol-3-yl)-*N*-(quinolin-8-yl)-4-(trifluoromethyl)benzamide (**17**):

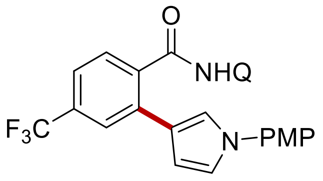

General Procedure **A** was followed using benzamide **1o** (79.1 mg). Column chromatography on silica gel (*n*-hexane/ethyl acetate = 4:1) yielded **17** (51.2 mg, 42%) as light brown oil.

^{1}H-NMR (300 MHz, $CDCl_3$) δ = 10.12 (s, 1H), 8.92 (dd, J = 7.5, 1.5 Hz, 1H), 8.51 (dd, J = 4.2, 1.7 Hz, 1H), 8.11 (dd, J = 8.3, 1.7 Hz, 1H), 7.90 (d, J = 8.0 Hz, 1H), 7.83 (d, J = 1.7 Hz, 1H), 7.65–7.58 (m, 2H), 7.57–7.50 (m, 1H), 7.33 (dd, J = 8.3, 4.2 Hz, 1H), 7.28 (t, J = 2.0 Hz, 1H), 7.11–6.99 (m, 2H), 6.91–6.76 (m, 3H), 6.50 (dd, J = 3.0, 1.8 Hz, 1H), 3.80 (s, 3H).

^{13}C-NMR (101 MHz, $CDCl_3$) δ = 167.7 (C_q), 158.0 (C_q), 148.2 (CH), 138.3 (q, $^{3}J_{C-F}$ = 34.3 Hz, CH), 136.3 (CH), 134.7 (C_q), 134.0 (C_q), 132.5 (CH), 129.5 (CH), 128.0 (C_q), 127.5 (CH), 126.6 (q, $^{4}J_{C-F}$ = 3.9 Hz, CH), 123.0 (CH), 123.0 (C_q), 122.2 (CH), 122.1 (CH), 121.7 (CH), 120.9 (CH), 119.1 (CH), 116.8 (CH), 114.7 (CH), 110.6 (CH), 55.7 (CH_3).

^{19}F-NMR (282 MHz, $CDCl_3$) δ = –62.9.

IR (ATR): 3338, 2931, 1672, 1518, 1484, 1357, 1248, 1168, 1124, 1074 cm^{-1}.

HR-MS (ESI): *m/z* calcd for $C_{28}H_{21}N_3O_2F_3$ $[M+H]^+$ 488.1580, found 488.1588.

2-(1-(4-Methoxyphenyl)-1*H*-pyrrol-3-yl)-4-(methylthio)-*N*-(quinolin-8-yl)benzamide (**18**):

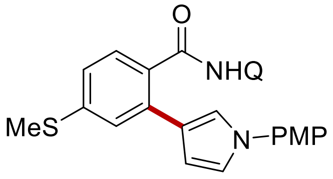

General Procedure **A** was followed using benzamide **1p** (73.6 mg). Column chromatography on silica gel (*n*-hexane/ethyl acetate = 4:1) yielded **18** (59.4 mg, 51%) as light brown oil.

^{1}H-NMR (400 MHz, $CDCl_3$) δ = 10.12 (s, 1H), 8.93 (dd, J = 7.7, 1.4 Hz, 1H), 8.43 (dd, J = 4.2, 1.7 Hz, 1H), 8.07 (dd, J = 8.3, 1.7 Hz, 1H), 7.80 (d, J = 8.2 Hz, 1H), 7.56 (t, J = 7.9 Hz, 1H), 7.48 (dd, J = 8.3, 1.3 Hz, 1H), 7.40 (d, J = 2.0 Hz, 1H), 7.32–7.27 (m, 1H), 7.23 (t, J = 2.0 Hz, 2H), 7.04 (d, J = 8.9 Hz, 2H), 6.89–6.80 (m, 3H), 6.45 (dd, J = 2.9, 1.8 Hz, 1H), 3.80 (s, 3H), 2.56 (s, 3H).

^{13}C-NMR (101 MHz, $CDCl_3$) δ = 168.3 (C_q), 157.8 (C_q), 148.0 (CH), 141.8 (C_q), 138.7 (C_q), 136.1 (CH), 135.1 (C_q), 134.8 (C_q), 134.1 (C_q), 132.0 (C_q), 129.9 (CH), 128.0 (C_q), 127.5 (CH), 127.1 (CH), 124.0 (C_q), 123.9 (CH), 122.0 (CH), 121.5 (CH), 121.5 (CH), 120.5 (CH), 118.8 (CH), 116.5 (CH), 114.7 (CH), 111.0 (CH), 55.7 (CH_3), 15.5 (CH_3).

IR (ATR): 2921, 2853, 1670, 1557, 1519, 1483, 1438, 1386, 1327, 1250 cm^{-1}.

HR-MS (ESI): *m/z* calcd for $C_{28}H_{24}N_3O_2S$ $[M+H]^+$ 466.1584, found 466.1593.

2-(1-(4-Methoxyphenyl)-1*H*-pyrrol-3-yl)-5-(prop-1-en-2-yl)-*N*-(quinolin-8-yl)benzamide (**19**):

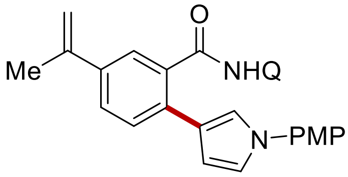

General Procedure **A** was followed using benzamide **1q** (72.1 mg). Column chromatography on silica gel (*n*-hexane/ethyl acetate = 4:1) yielded **19** (50.6 mg, 44%) as light brown solid.

M. p.: 171–173 °C.

^{1}H-NMR (400 MHz, $CDCl_3$) δ = 10.12 (s, 1H), 8.96 (dd, J = 7.6, 1.3 Hz, 1H), 8.48 (dd, J = 4.2, 1.7 Hz, 1H), 8.09 (dd, J = 8.3, 1.8 Hz, 1H), 7.91 (d, J = 2.0 Hz, 1H), 7.64–7.57 (m, 2H), 7.57–7.48 (m, 2H), 7.30 (dd, J = 8.2, 4.2 Hz, 1H), 7.23 (t, J = 2.0 Hz, 1H), 7.06–6.99 (m, 2H), 6.92–6.75 (m, 3H), 6.48 (dd, J = 2.9, 1.8 Hz, 1H), 5.49 (t, J = 1.4, 0.8 Hz, 1H), 5.14 (t, J = 1.5 Hz, 1H), 3.80 (s, 3H), 2.27–2.14 (m, 3H).

^{13}C-NMR (101 MHz, $CDCl_3$) δ = 169.1 (C_q), 157.8 (C_q), 148.1 (CH), 142.4 (C_q), 139.3 (C_q), 138.7 (C_q), 136.1 (CH), 135.3 (C_q), 135.1 (C_q), 134.2 (C_q), 133.0 (C_q), 129.7 (CH), 128.0 (C_q), 127.5 (CH), 127.4 (CH), 126.2 (CH), 124.0 (C_q), 122.0 (CH), 121.7 (CH), 121.5 (CH), 120.4 (CH), 118.6 (CH), 116.6 (CH), 114.6 (CH), 112.9 (CH_2), 110.8 (CH), 55.7 (CH_3), 21.9 (CH_3).

IR (ATR): 3347, 2924, 2836, 1668, 1518, 1484, 1424, 1384, 1327, 1248 cm^{-1}.

HR-MS (ESI): *m/z* calcd for $C_{30}H_{26}N_3O_2$ $[M+H]^+$ 460.2020, found 460.2019.

6-(1-(4-Methoxyphenyl)-1*H*-pyrrol-3-yl)-2, 3-dimethyl-*N*-(quinolin-8-yl)benzamide (**20**):

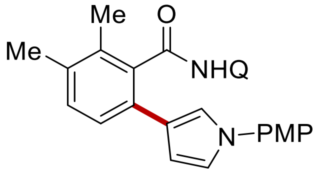

General Procedure **A** was followed using benzamide **1r** (69.1 mg). Column chromatography on silica gel (*n*-hexane/ethyl acetate = 4:1) yielded **20** (62.7 mg, 56%) as light brown oil.

^{1}H-NMR (400 MHz, $CDCl_3$) δ = 9.91 (s, 1H), 9.01 (dd, J = 7.6, 1.4 Hz, 1H), 8.65 (dd, J = 4.2, 1.7 Hz, 1H), 8.13 (dd, J = 8.3, 1.7 Hz, 1H), 7.61 (t, J = 7.9 Hz, 1H), 7.54 (dd, J = 8.3, 1.4 Hz, 1H), 7.46–7.34 (m, 2H), 7.28 (s, 1H), 7.23 (t, J = 2.0 Hz, 1H), 7.07–6.97 (m, 2H), 6.89–6.62 (m, 3H), 6.53 (dd, J = 2.9, 1.8 Hz, 1H), 3.79 (s, 3H), 2.41 (s, 3H), 2.37 (s, 3H).

^{13}C-NMR (101 MHz, $CDCl_3$) δ = 170.2 (C_q), 157.6 (C_q), 148.3 (CH), 138.7 (C_q), 136.3 (C_q), 136.3 (CH), 135.1 (C_q), 135.0 (C_q), 134.3 (C_q), 133.6 (C_q), 131.1 (C_q), 130.8 (CH), 128.1 (C_q), 127.5 (CH), 126.3 (CH), 124.6 (C_q), 121.9 (CH), 121.8 (CH), 121.7 (CH), 119.9 (CH), 118.0 (CH), 116.8 (CH), 114.5 (CH), 110.4 (CH), 55.6 (CH_3), 20.2 (CH_3), 16.8 (CH_3).

IR (ATR): 3345, 2957, 2933, 1671, 1516, 1483, 1424, 1385, 1326, 1247 cm^{-1}.

HR-MS (ESI): *m/z* calcd for $C_{29}H_{26}N_3O_2$ $[M+H]^+$ 448.2020, found 448.2016.

2-(1-(4-Methoxyphenyl)-1*H*-pyrrol-3-yl)-4, 6-dimethyl-*N*-(quinolin-8-yl)benzamide (**21**):

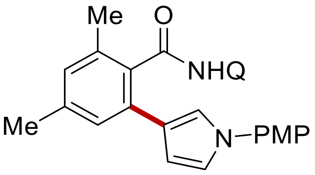

General Procedure **A** was followed using benzamide **1s** (69.1 mg). Column chromatography on silica gel (*n*-hexane/ethyl acetate = 4:1) yielded **21** (90.6 mg, 81%) as light brown oil.

^{1}H-NMR (400 MHz, $CDCl_3$) δ = 9.88 (s, 1H), 8.98 (dd, J = 7.7, 1.3 Hz, 1H), 8.60 (dd, J = 4.1, 1.7 Hz, 1H), 8.09 (dd, J = 8.3, 1.7 Hz, 1H), 7.59 (t, J = 8.0 Hz, 1H), 7.50 (dd, J = 8.3, 1.4 Hz, 1H), 7.34 (dd, J = 8.3, 4.2 Hz, 1H), 7.25 (s, 1H), 7.23 (t, J = 2.0 Hz, 1H), 7.04–6.95 (m, 3H), 6.86–6.70 (m, 3H), 6.53 (dd, J = 2.9, 1.8 Hz, 1H), 3.77 (s, 3H), 2.48 (s, 3H), 2.41 (s, 3H).

^{13}C-NMR (101 MHz, $CDCl_3$) δ = 169.9 (C_q), 157.6 (C_q), 148.2 (CH), 139.0 (C_q), 138.6 (C_q), 136.2 (CH), 135.5 (C_q), 135.1 (C_q), 134.2 (C_q), 133.4 (C_q), 128.9 (CH), 128.0 (C_q), 127.4 (CH), 127.1 (CH), 124.6 (C_q), 121.9 (CH), 121.7 (CH), 121.6 (CH), 120.0 (CH), 118.2 (CH), 116.7 (CH), 114.5 (CH), 110.4 (CH), 55.6 (CH_3), 21.4 (CH_3), 19.7 (CH_3).

IR (ATR): 3348, 2922, 2835, 1735, 1670, 1515, 1481, 1442, 1325, 1245 cm^{-1}.

HR-MS (ESI): *m/z* calcd for $C_{29}H_{26}N_3O_2$ $[M+H]^+$ 448.2020, found 448.2015.

2-(1-(4-Methoxyphenyl)-1*H*-pyrrol-3-yl)-4, 5-dimethyl-*N*-(quinolin-8-yl)benzamide (**22**):

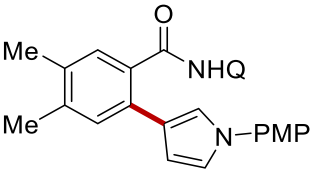

General Procedure **A** was followed using benzamide **1t** (69.1 mg). Column chromatography on silica gel (*n*-hexane/ethyl acetate = 4:1) yielded **22** (77.0 mg, 69%) as brown solid.

M. p.: 152–153 °C.

^{1}H-NMR (400 MHz, $CDCl_3$) δ = 10.13 (s, 1H), 8.95 (dd, J = 7.7, 1.3 Hz, 1H), 8.65–8.42 (m, 1H), 8.06 (dd, J = 8.3, 1.7 Hz, 1H), 7.65 (s, 1H), 7.56 (t, J = 8.0 Hz, 1H), 7.46 (dd, J = 8.2, 1.3 Hz, 1H), 7.34 (s, 1H), 7.28 (d, J = 4.2 Hz, 1H), 7.20 (t, J = 2.0 Hz, 1H), 7.07–6.95 (m, 2H), 6.92–6.78 (m, 3H), 6.44 (dd, J = 2.8, 1.7 Hz, 1H), 3.79 (s, 3H), 2.36 (d, J = 2.1 Hz, 6H).

^{13}C-NMR (101 MHz, $CDCl_3$) δ = 168.9 (C_q), 157.7 (C_q), 147.9 (CH), 139.3 (C_q), 138.8 (C_q), 136.0 (CH), 135.2 (C_q), 135.0 (C_q), 134.2 (C_q), 132.9 (C_q), 131.6 (C_q), 131.3 (CH), 130.4 (CH), 128.0 (C_q), 127.5 (CH), 124.3 (C_q), 121.9 (CH), 121.4 (CH), 121.4 (CH), 120.2 (CH), 118.4 (CH), 116.4 (CH), 114.6 (CH), 111.0 (CH), 55.6 (CH_3), 19.9 (CH_3), 19.4 (CH_3).

IR (ATR): 3347, 2915, 1734, 1662, 1514, 1482, 1423, 1383, 1325, 1243 cm^{-1}.

HR-MS (ESI): *m/z* calcd for $C_{29}H_{26}N_3O_2$ $[M+H]^+$ 448.2020, found 448.2017.

5-Methoxy-2-(1-(4-methoxyphenyl)-1*H*-pyrrol-3-yl)-4-methyl-*N*-(quinolin-8-yl)benzamide (23):

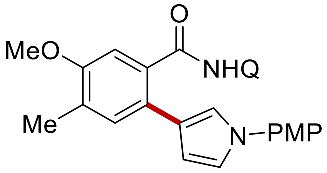

General Procedure **A** was followed using benzamide **1u** (73.1 mg). Column chromatography on silica gel (*n*-hexane/ethyl acetate = 4:1) yielded **23** (60.3 mg, 52%) as colourless solid.

M. p.: 126–127 °C.

^{1}H-NMR (400 MHz, $CDCl_3$) δ = 10.20 (s, 1H), 8.95 (dd, J = 7.7, 1.4 Hz, 1H), 8.39 (dd, J = 4.2, 1.7 Hz, 1H), 8.05 (dd, J = 8.2, 1.7 Hz, 1H), 7.56 (t, J = 8.0 Hz, 1H), 7.46 (dd, J = 8.3, 1.3 Hz, 1H), 7.39 (s, 1H), 7.32 (d, J = 1.0 Hz, 1H), 7.26–7.22 (m, 1H), 7.17 (t, J = 2.0 Hz, 1H), 7.05–6.96 (m, 2H), 6.84–6.71 (m, 3H), 6.41 (dd, J = 2.9, 1.7 Hz, 1H), 3.93 (s, 3H), 3.79 (s, 3H), 2.32 (s, 3H).

^{13}C-NMR (101 MHz, $CDCl_3$) δ = 168.5 (C_q), 157.6 (C_q), 156.6 (C_q), 147.9 (CH), 138.8 (C_q), 136.0 (CH), 135.2 (C_q), 134.3 (C_q), 133.5 (C_q), 132.7 (CH), 129.6 (C_q), 127.9 (C_q), 127.5 (CH), 126.4 (C_q), 124.2 (C_q), 121.8 (CH), 121.4 (CH), 120.1 (CH), 118.3 (CH), 116.4 (CH), 114.6 (CH), 111.2 (CH), 110.8 (CH), 55.7 (CH_3), 55.6 (CH_3), 16.3 (CH_3).

IR (ATR): 3310, 2932, 2836, 1712, 1664, 1518, 1484, 1326, 1247, 1226 cm^{-1}.

HR-MS (ESI): *m/z* calcd for $C_{29}H_{26}N_3O_3$ $[M+H]^+$ 464.1969, found 464.1969.

3-(1-(4-Methoxyphenyl)-1*H*-pyrrol-3-yl)-*N*-(quinolin-8-yl)-3a, 7a-dihydrobenzo[*b*]thiophene-2-carboxamide (24):

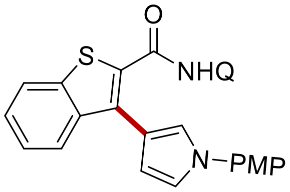

General Procedure **A** was followed using benzamide **1x** (76.0 mg). Column chromatography on silica gel (*n*-hexane/ethyl acetate = 4:1) yielded **24** (83.0 mg, 70%) as brown solid.

M. p.: 194–196 °C.

^{1}H-NMR (400 MHz, $CDCl_3$) δ = 10.91 (s, 1H), 8.95 (dd, J = 7.7, 1.3 Hz, 1H), 8.18 (dd, J = 4.2, 1.7 Hz, 1H), 8.04 (dd, J = 8.2, 1.7 Hz, 1H), 7.94–7.90 (m, 1H), 7.80 (dt, J = 8.0, 1.0 Hz, 1H), 7.54 (t, J = 8.0 Hz, 1H), 7.45 (ddd, J = 8.1, 5.8, 1.4 Hz, 2H), 7.42–7.38 (m, 1H), 7.36 (q, J = 1.8 Hz, 1H), 7.31–7.27 (m, 3H), 7.20 (dd, J = 8.3, 4.2 Hz, 1H), 6.93 (d, J = 8.9 Hz, 2H), 6.57 (dd, J = 2.9, 1.7 Hz, 1H), 3.84 (s, 3H).

^{13}C-NMR (101 MHz, $CDCl_3$) δ = 161.7 (C_q), 158.1 (C_q), 148.0 (CH), 141.7 (C_q), 140.2 (C_q), 138.9 (C_q), 136.4 (C_q), 136.0 (CH), 135.0 (C_q), 134.1 (C_q), 132.8 (C_q), 128.0 (C_q), 127.6 (CH), 126.6 (CH), 125.3 (CH), 124.6 (CH), 122.6 (CH), 122.0 (CH), 121.7 (CH), 121.4 (CH), 121.2 (CH), 120.2 (CH), 117.0 (CH), 116.8 (C_q), 114.9 (CH), 112.4 (CH), 55.7 (CH_3).

IR (ATR): 2909, 2851, 1711, 1649, 1516, 1486, 1463, 1326, 1272, 1247 cm^{-1}.

HR-MS (ESI): *m/z* calcd for $C_{29}H_{22}N_3O_2S$ $[M+H]^+$ 476.1427, found 476.1426.

4-Methoxy-2-(1-(4-methoxyphenyl)-1*H*-pyrrol-3-yl)-*N*-(5-methylquinolin-8-yl)benzamide (**25**):

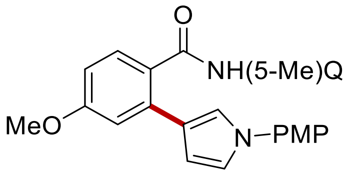

General Procedure **A** was followed using benzamide **1w** (73.1 mg). Column chromatography on silica gel (*n*-hexane/ethyl acetate = 4:1) yielded **25** (66.1 mg, 57%) as light brown solid.

M. p.: 172–173 °C.

^{1}H-NMR (400 MHz, $CDCl_3$) δ = 10.08 (s, 1H), 8.81 (d, J = 1.8 Hz, 1H), 8.33 (dd, J = 4.2, 1.7 Hz, 1H), 7.96 (dd, J = 8.3, 1.7 Hz, 1H), 7.86 (d, J = 8.6 Hz, 1H), 7.24–7.19 (m, 3H), 7.07–7.02 (m, 3H), 6.93 (dd, J = 8.6, 2.6 Hz, 1H), 6.86–6.79 (m, 3H), 6.45 (dd, J = 2.9, 1.8 Hz, 1H), 3.90 (s, 3H), 3.80 (s, 3H), 2.63–2.34 (m, 3H).

^{13}C-NMR (101 MHz, $CDCl_3$) δ = 168.3 (C_q), 161.2 (C_q), 157.8 (C_q), 147.0 (CH), 137.7 (C_q), 137.5 (C_q), 136.1 (C_q), 135.3 (CH), 134.8 (C_q), 134.2 (C_q), 131.4 (CH), 128.3 (C_q), 128.0 (C_q), 124.4 (C_q), 122.0 (CH), 121.5 (CH), 120.4 (CH), 118.8 (CH), 118.4 (CH), 115.1 (CH), 114.6 (CH), 112.2 (CH), 111.1 (CH), 55.7 (CH_3), 55.6 (CH_3), 22.5 (CH_3).

IR (ATR): 3325, 3309, 2935, 1712, 1660, 1600, 1517, 1422, 1248, 1221 cm^{-1}.

HR-MS (ESI): *m/z* calcd for $C_{29}H_{26}N_3O_3$ $[M+H]^+$ 464.1969, found 464.1976.

2-(1-(4-Methoxyphenyl)-1*H*-pyrrol-3-yl)-*N*-(6-methylquinolin-8-yl)benzamide (**26**):

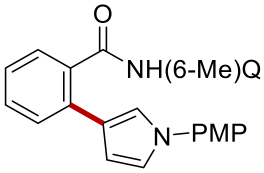

General Procedure **A** was followed using benzamide **1x** (65.6 mg). Column chromatography on silica gel (*n*-hexane/ethyl acetate = 4:1) yielded **26** (70.4 mg, 65%) as light brown solid.

M. p.: 179–180 °C.

^{1}H-NMR (400 MHz, $CDCl_3$) δ = 10.08 (s, 1H), 8.83 (d, J = 1.7 Hz, 1H), 8.40 (dd, J = 4.2, 1.7 Hz, 1H), 7.97 (dd, J = 8.3, 1.7 Hz, 1H), 7.82 (dd, J = 7.6, 1.4 Hz, 1H), 7.57 (dd, J = 7.8, 1.3 Hz, 1H), 7.50 (td, J = 7.5, 1.5 Hz, 1H), 7.38 (td, J = 7.5, 1.4 Hz, 1H), 7.25–7.22 (m, 2H), 7.05 (d, J = 8.9 Hz, 2H), 6.89–6.79 (m, 3H), 6.48 (dd, J = 2.9, 1.8 Hz, 1H), 3.80 (s, 3H), 2.57 (d, J = 1.0 Hz, 3H).

^{13}C-NMR (101 MHz, $CDCl_3$) δ = 168.9 (C_q), 157.8 (C_q), 147.1 (CH), 137.6 (C_q), 137.5 (C_q), 135.5 (C_q), 135.4 (CH), 134.6 (C_q), 134.2 (C_q), 134.0 (C_q), 130.4 (CH), 129.9 (CH), 129.0 (CH), 128.0 (C_q), 126.5 (CH), 124.3 (C_q), 122.0 (CH), 121.6 (CH), 120.6 (CH), 120.4 (CH), 118.6 (CH), 114.6 (CH), 110.9 (CH), 55.6 (CH_3), 22.5 (CH_3).

IR (ATR): 3330, 2907, 2851, 1729, 1669, 1518, 1473, 1425, 1276, 1247 cm^{-1}.

HR-MS (ESI) *m/z* calcd for $C_{28}H_{24}N_3O_2$ $[M+H]^+$ 434.1863, found 434.1859.

2-(1-(4-Methoxyphenyl)-1*H*-pyrrol-3-yl)-*N*-(5-methoxyquinolin-8-yl)benzamide (27):

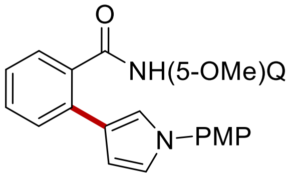

General Procedure **A** was followed using benzamide **1z** (69.1 mg). Column chromatography on silica gel (*n*-hexane/ethyl acetate = 4:1) yielded **27** (66.2 mg, 59%) as yellow oil.

^{1}H-NMR (400 MHz, $CDCl_3$) δ = 9.87 (s, 1H), 8.86 (d, *J* = 8.5 Hz, 1H), 8.49–8.42 (m, 2H), 7.80 (dd, *J* = 7.7, 1.4 Hz, 1H), 7.55 (d, *J* = 1.3 Hz, 1H), 7.46 (td, *J* = 7.5, 1.5 Hz, 1H), 7.36 (dd, *J* = 7.5, 1.3 Hz, 1H), 7.23 (dd, *J* = 3.7, 1.8 Hz, 2H), 7.06–6.99 (m, 2H), 6.84 (d, *J* = 8.6 Hz, 1H), 6.82–6.75 (m, 3H), 6.46 (dd, *J* = 2.9, 1.8 Hz, 1H), 3.95 (d, *J* = 1.2 Hz, 3H), 3.75 (d, *J* = 1.3 Hz, 3H).

^{13}C-NMR (101 MHz, $CDCl_3$) δ = 168.5 (C_q), 157.7 (C_q), 150.4 (C_q), 148.5 (CH), 139.4 (C_q), 135.6 (C_q), 134.1 (C_q), 133.9 (C_q), 131.0 (CH), 130.2 (CH), 129.7 (CH), 128.9 (CH), 128.5 (C_q), 126.4, 124.3 (C_q), 121.8 (CH), 120.6 (CH), 120.4 (C_q), 120.2 (CH), 118.5 (CH), 116.7 (CH), 114.6 (CH), 110.8 (CH), 104.4 (CH), 55.8 (CH_3), 55.6 (CH_3).

IR (ATR): 2939, 2891, 1672, 1519, 1493, 1460, 1396, 1286, 1248, 1091 cm^{-1}.

HR-MS (ESI) *m/z* calcd for $C_{28}H_{24}N_3O_3$ $[M+H]^+$ 450.1807, found 450.1812.

4-Ethyl-2-(1-(4-methoxyphenyl)-1*H*-pyrrol-3-yl)-*N*-(quinolin-8-yl)benzamide (**28**):

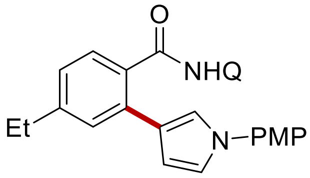

General Procedure **A** was followed using benzamide **1y** (68.6 mg). Column chromatography on silica gel (*n*-hexane/ethyl acetate = 4:1) yielded **28** (67.1 mg, 60%) as light brown solid.

M. p.: 177–178 °C.

^{1}H-NMR (400 MHz, $CDCl_3$) δ = 10.11 (s, 1H), 8.94 (dd, *J* = 7.6, 1.4 Hz, 1H), 8.45 (dd, *J* = 4.2, 1.7 Hz, 1H), 8.07 (dd, *J* = 8.3, 1.7 Hz, 1H), 7.78 (d, *J* = 7.8 Hz, 1H), 7.57 (t, *J* = 7.9 Hz, 1H), 7.47 (dd, *J* = 8.3, 1.4 Hz, 1H), 7.39 (d, *J* = 1.8 Hz, 1H), 7.28 (dd, *J* = 8.3, 4.2 Hz, 1H), 7.23 (dt, *J* = 5.7, 1.9 Hz, 2H), 7.09–7.01 (m, 2H), 6.90–6.80 (m, 3H), 6.47 (dd, *J* = 2.9, 1.8 Hz, 1H), 3.80 (s, 3H), 2.74 (q, *J* = 7.6 Hz, 2H), 1.32 (t, *J* = 7.6 Hz, 3H).

^{13}C-NMR (101 MHz, $CDCl_3$) δ = 168.9 (C_q), 157.8 (C_q), 148.0 (CH), 146.9 (C_q), 138.8 (C_q), 136.1 (CH), 135.2 (C_q), 134.2 (C_q), 134.1 (C_q), 133.1 (C_q), 129.5 (CH), 129.4 (CH), 128.0 (CH), 127.5 (C_q), 126.2 (CH), 124.6 (C_q), 122.0 (CH), 121.5 (CH), 120.3 (CH), 118.6 (CH), 116.5 (CH), 114.6 (CH), 111.0 (CH), 55.7 (CH_3), 29.0 (CH_2), 15.6 (CH_3).

IR (ATR): 3340, 2930, 2910, 2851, 1670, 1518, 1483, 1385, 1326, 1248 cm^{-1}.

HR-MS (ESI) *m/z* calcd for $C_{29}H_{26}N_3O_3$ $[M+H]^+$ 448.2020, found 448.2015.

***rac*-3-(4-Methoxyphenyl)-4-(quinolin-8-yl)-1, 2, 3, 3a, 4, 9b-hexahydro-5*H*-pyrrolo[2, 3-c]isoquinolin-5-one (29):**

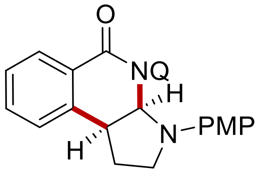

General Procedure **A** was followed applying 4.0 mA and using benzamide **1a** (62.0 mg). Column chromatography on silica gel (Et_2O) yielded **29** (25.2 mg, 24%) as colourless solid.

M. p.: 209–211 °C.

^{1}H-NMR (300 MHz, $CDCl_3$) δ = 8.95 (dd, J = 4.2, 1.8 Hz, 1H), 8.24 (dd, J = 7.7, 1.5 Hz, 1H), 8.09 (dd, J = 8.3, 1.7 Hz, 1H), 7.67–7.48 (m, 2H), 7.48–7.35 (m, 4H), 7.35–7.27 (m, 2H), 6.44 (d, J = 5.3 Hz, 1H), 6.21–6.12 (m, 2H), 5.99–5.82 (m, 2H), 3.73 (dt, J = 8.8, 5.4 Hz, 1H), 3.52 (s, 3H), 3.41 (q, J = 8.8 Hz, 1H), 2.82–2.43 (m, 2H).

^{13}C-NMR (101 MHz, $CDCl_3$) δ = 164.6 (C_q), 151.1 (C_q), 150.7 (CH), 145.5 (C_q), 140.3 (C_q), 138.7 (C_q), 137.0 (C_q), 136.5 (CH), 132.3 (CH), 131.8 (CH), 129.6 (CH), 129.4 (C_q), 128.3 (CH), 127.7 (CH), 127.4 (C_q), 127.4 (CH), 126.1 (CH), 121.4 (CH), 113.8 (CH), 112.4 (CH), 75.5 (CH), 55.7 (CH_3), 47.6 (CH_2), 43.6 (CH), 33.1 (CH_2).

IR (ATR): 2971, 2922, 1655, 1602, 1514, 1487, 1463, 1398, 1273, 1244 cm^{-1}.

HR-MS (ESI) *m/z* calcd for $C_{27}H_{24}N_3O_2$ $[M+H]^+$ 422.1863, found 422.1869.

***rac*-3-(4-Methoxyphenyl)-7-methyl-4-(quinolin-8-yl)-1,2,3,3a,4,9b-hexahydro-5*H*-pyrrolo[2,3-c]isoquinolin-5-one (30):**

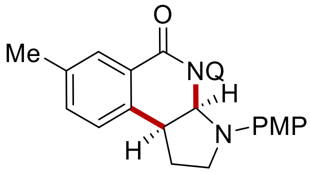

General Procedure **A** was followed applying 4.0 mA and using benzamide **1c** (65.5 mg). Column chromatography on silica gel (Et_2O) yielded **30** (25.1 mg, 23%) as yellow oil.

^{1}H-NMR (400 MHz, $CDCl_3$) δ = 8.95 (dd, J = 4.2, 1.8 Hz, 1H), 8.18–7.99 (m, 2H), 7.59 (dd, J = 8.3, 1.5 Hz, 1H), 7.42–7.37 (m, 2H), 7.36–7.33 (m, 3H), 7.24 (d, J = 7.7 Hz, 1H), 6.40 (t, J = 6.5 Hz, 1H), 6.23–6.08 (m, 2H), 5.95–5.83 (m, 2H), 3.85–3.66 (m, 1H), 3.52 (s, 3H), 3.44–3.32 (m, 1H), 2.68–2.56 (m, 2H), 2.42 (s, 3H).

^{13}C-NMR (101 MHz, $CDCl_3$) δ = 164.8 (C_q), 151.0 (C_q), 150.7 (CH), 145.5 (C_q), 140.3 (C_q), 137.4 (C_q), 137.1 (C_q), 136.4 (CH), 135.7 (C_q), 133.1 (CH), 131.9 (CH), 129.9 (CH), 129.4 (C_q), 128.3 (CH), 127.3 (CH), 127.2 (C_q), 126.1 (CH), 121.3 (CH), 113.8 (CH), 112.4 (CH), 112.4 (CH), 75.6 (CH), 55.7 (CH_3), 47.6 (CH_2), 43.3 (CH), 33.2 (CH_2), 21.3 (CH_3).

IR (ATR=: 2926, 2912, 1653, 1640, 1513, 1427, 1390, 1379, 1274, 1242 cm^{-1}.

HR-MS (ESI) *m/z* calcd for $C_{28}H_{26}N_3O_2$ $[M+H]^+$ 436.2020, found 436.2022.

***rac*-3-(4-Methoxyphenyl)-8-phenoxy-4-(quinolin-8-yl)-1,2,3,3a,4,9b-hexahydro-5*H*-pyrrolo[2,3-c]isoquinolin-5-one (31):**

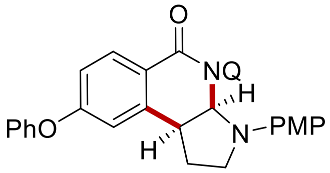

General Procedure **A** was followed applying 4.0 mA and using benzamide **1h** (85.1 mg). Column chromatography on silica gel (Et_2O) yielded **31** (21.8 mg, 17%) as yellow oil.
^{1}H-NMR (400 MHz, $CDCl_3$) δ = 8.97 (dd, J = 4.2, 1.7 Hz, 1H), 8.19 (d, J = 8.5 Hz, 1H), 8.10 (d, J = 1.8 Hz, 1H), 7.60 (dd, J = 8.2, 1.5 Hz, 1H), 7.46–7.36 (m, 4H), 7.31–7.27 (m, 1H), 7.26 (s, 1H), 7.24–7.18 (m, 1H), 7.18–7.11 (m, 2H), 6.99 (dd, J = 8.6, 2.5 Hz, 1H), 6.92 (d, J = 2.4 Hz, 1H), 6.43 (d, J = 5.3 Hz, 1H), 6.21–6.11 (m, 2H), 5.96–5.77 (m, 2H), 3.72 (td, J = 8.5, 2.1 Hz, 1H), 3.52 (s, 3H), 3.49–3.40 (m, 1H), 2.68–2.52 (m, 2H).
^{13}C-NMR (101 MHz, $CDCl_3$) δ = 164.2 (C_q), 161.2 (C_q), 155.9 (C_q), 151.0 (C_q), 150.7 (CH), 145.5 (C_q), 141.0 (C_q), 140.2 (C_q), 137.0 (C_q), 136.5 (CH), 131.9 (CH), 131.9 (CH), 130.2 (CH), 129.4 (C_q), 128.3 (CH), 126.1 (CH), 124.5 (CH), 122.0 (C_q), 121.3 (CH), 120.2 (CH), 116.9 (CH), 115.9 (CH), 113.8 (CH), 112.4 (CH), 75.5 (CH), 55.7 (CH_3), 47.5 (CH_2), 43.7 (CH), 33.0 (CH_2).
IR (ATR): 3043, 2931, 1650, 1588, 1512, 1487, 1391, 1379, 1244, 1209 cm^{-1}.
HR-MS (ESI) *m/z* calcd for $C_{33}H_{28}N_3O_3$ $[M+H]^+$ 514.2125, found 514.2126.

2-(1-(4-Methoxyphenyl)-1*H*-pyrrol-3-yl)-*N*-(1,2,3,4-tetrahydroquinolin-8-yl)benzamide(32):

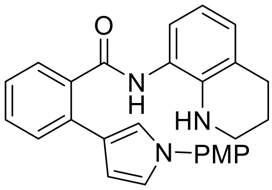

3 (70 mg, 0.16 mmol) and palladium on coal (10 mol%, 17.0 mg) were mixed with ethanol (3.0 mL) in a reaction tube and placed in a stainless steel high-pressure autoclave. This was flushed three times with hydrogen before a pressure of 50 bar of hydrogen was applied and heated to 50 °C for 24 hours. After cooling down to room temperature, the mixture was filtered through celite. Purification via column chromatography on silica gel (EtOAc/*n*-hexane, 3:1) yielded **32** (60.0 mg, 88%) as colourless solid.
M. p.: 152–154 °C.
^{1}H-NMR (400 MHz, $CDCl_3$) δ = 7.86–7.72 (m, 1H), 7.49–7.45 (m, 2H), 7.37 (ddd, J = 7.6, 6.1, 2.6 Hz, 1H), 7.32–7.27 (m, 2H), 7.23 (t, J = 2.0 Hz, 1H), 7.14 (dd, J = 7.8, 1.5 Hz, 1H), 7.09 (t, J = 2.6 Hz, 1H), 7.02 (s, 1H), 6.96–6.91 (m, 2H), 6.81 (dd, J = 7.5, 1.4 Hz, 1H), 6.62 (t, J = 7.7 Hz, 1H), 6.56 (dd, J = 2.8, 1.8 Hz, 1H), 3.84 (s, 3H), 3.09–2.98 (m, 2H), 2.69 (t, J = 6.3 Hz, 2H), 1.80–1.67 (m, 2H).
^{13}C-NMR (101 MHz, $CDCl_3$) δ = 169.2 (C_q), 158.2 (C_q), 138.6 (C_q), 135.5 (C_q), 133.8 (C_q), 133.1 (C_q), 130.3 (CH), 130.0 (CH), 129.1 (CH), 127.2 (CH), 126.9 (CH), 124.6 (C_q), 123.8 (C_q), 123.3 (C_q), 122.7 (CH), 122.1 (CH), 120.4 (CH), 118.7 (CH), 117.2 (CH), 114.9 (CH), 111.1 (CH), 55.7 (CH_3), 42.3 (CH_2), 27.4 (CH_2), 21.9 (CH_2).
IR (ATR): 2929, 2846, 1633, 1521, 1502, 1459, 1446, 1357, 1312, 1264 cm^{-1}.
HR-MS (ESI): *m/z* calcd for $C_{27}H_{26}N_3O_2$ $[M+H]^+$ 424.2020, found 424.2020.

***tert*-Butyl-2-(1-(4-methoxyphenyl)-1*H*-pyrrol-3-yl)-5-methylbenzoyl)(quinolin-8-yl)carbamate (33)**

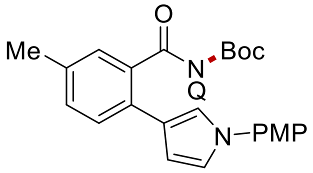

5 (107.5 mg, 0.25 mmol) was dissolved in dry THF (10 mL) and was treated with sodium hydride (40.0 mg, 4.0 equiv.) and Boc_2O (218 mg, 4.0 equiv.) and the mixture was stirred for 18 h at room temperature. Purification via column chromatography on silica gel (*n*-hexane/EtOAc, 3:1) yielded **33** (106.8 mg, 80%) as yellow oil.

^{1}H-NMR (400 MHz, $CDCl_3$) δ = 8.90–8.72 (m, 1H), 8.14 (dd, *J* = 8.3, 1.7 Hz, 1H), 7.80 (dd, *J* = 7.8, 1.9 Hz, 1H), 7.55 (s, 1H), 7.49 (t, *J* = 7.6 Hz, 2H), 7.40–7.36 (m, 2H), 7.34 (dd, *J* = 8.9, 2.3 Hz, 3H), 7.22 (ddd, *J* = 7.8, 1.9, 0.8 Hz, 1H), 7.00 (t, *J* = 2.6 Hz, 1H), 6.96–6.89 (m, 2H), 6.59 (d, *J* = 2.7 Hz, 1H), 3.85 (s, 3H), 2.40 (s, 3H), 1.14 (s, 9H).

^{13}C-NMR (101 MHz, $CDCl_3$) δ = 173.4 (C_q), 157.7 (C_q), 152.9 (C_q), 150.5 (CH), 144.6 (C_q), 136.9 (C_q), 136.6 (C_q), 136.1 (CH), 135.2 (C_q), 134.5 (C_q), 130.9 (C_q), 130.2 (CH), 129.3 (CH), 129.2 (C_q), 129.0 (CH), 128.3 (CH), 127.5 (CH), 126.2 (CH), 124.5 (C_q), 122.2 (CH), 121.6 (CH), 119.8 (CH), 118.7 (CH), 114.7 (CH), 110.7 (CH), 83.1 (C_q), 55.7 (CH_3), 27.6 (CH_3), 21.1 (CH_3).

IR (ATR): 3049, 2977, 2926, 1737, 1682, 1518, 1367, 1350, 1247, 1155 cm^{-1}.

HR-MS (ESI): *m/z* calcd for $C_{33}H_{32}N_3O_4$ $[M+H]^+$ 534.2387, found 534.2378.

2-(1-(2,4-Dimethoxyphenyl)-1*H*-pyrrol-3-yl)-*N*-(5-methoxyquinolin-8-yl)benzamide (34):

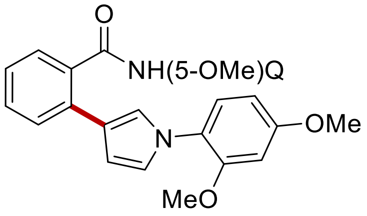

General Procedure **A** was followed using benzamide **1z** (69.1 mg) and pyrrolidine **2d** (103.8). Column chromatography on silica gel (*n*-hexane/ethyl acetate = 4:1) yielded **34** (39.0 mg, 32%) as yellow oil.

^{1}H-NMR (300 MHz, $CDCl_3$) δ = 9.87 (s, 1H), 8.87 (d, *J* = 8.5 Hz, 1H), 8.56 (dd, *J* = 4.2, 1.7 Hz, 1H), 8.51 (dd, *J* = 8.4, 1.8 Hz, 1H), 7.78 (dd, *J* = 7.7, 1.5 Hz, 1H), 7.58 (dd, *J* = 7.7, 1.3 Hz, 1H), 7.47 (td, *J* = 7.5, 1.5 Hz, 1H), 7.38–7.28 (m, 2H), 7.15 (t, *J* = 2.0 Hz, 1H), 6.87 (d, *J* = 8.6 Hz, 1H), 6.80–6.69 (m, 2H), 6.47 (dd, *J* = 4.2, 2.7 Hz, 2H), 6.40–6.26 (m, 1H), 4.00 (s, 3H), 3.79 (s, 3H), 3.55 (s, 3H).

^{13}C-NMR (75 MHz, $CDCl_3$) δ = 168.7 (C_q), 159.3 (C_q), 153.7 (C_q), 150.3 (C_q), 148.5 (CH), 139.5 (C_q), 135.7 (C_q), 134.2 (C_q), 131.0 (CH), 130.2 (CH), 129.8 (CH), 129.0 (CH), 128.8 (C_q), 126.2 (CH), 126.1 (CH), 123.8 (C_q), 122.9 (CH), 121.3 (CH), 120.6 (CH), 120.5 (C_q), 116.7 (CH), 109.4 (CH), 104.5 (CH), 104.3 (CH), 99.9 (CH), 55.9 (CH_3), 55.7 (CH_3), 55.7 (CH_3).

IR (ATR): 2937, 2836, 1659, 1594 1517, 1491, 1461, 1267, 1207, 1150 cm^{-1}.

HR-MS (ESI) *m/z* calcd for $C_{29}H_{26}N_3O_4$ $[M+H]^+$ 480.1918, found 480.1919.

V. Unsuccessful Examples

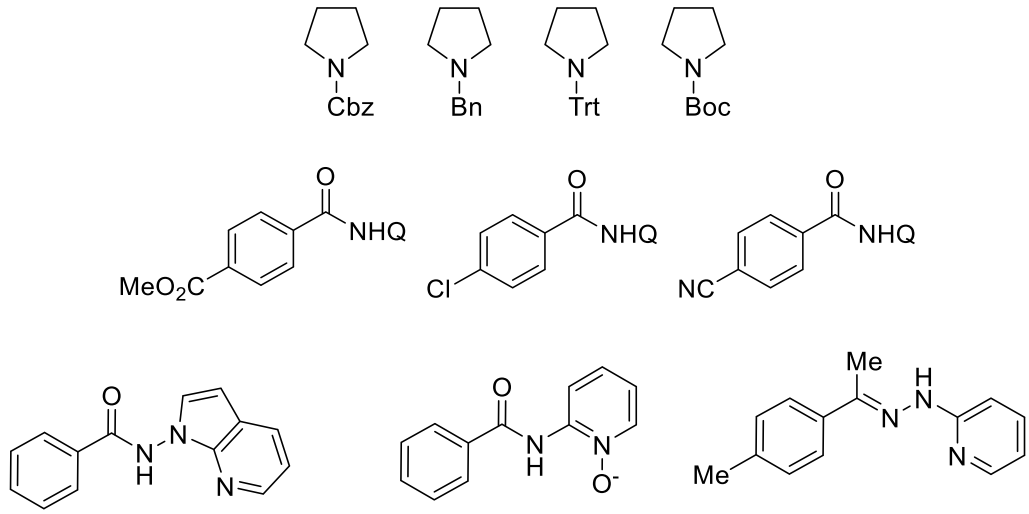

VI. Gram-Scale Reaction

Scale-up reaction was performed by using a bottle-type cell (Figure S3). Graphite anode (30 x 30 mm × 6 mm) and Ni foam cathode (20 x 20 mm × 1.4 mm) were used as electrodes respectively. Following general procedure **A**, **1c** (5.0 mmol), **2** (10 mmol) were dissolved in DMA (80 mL) and a CCE at 60.0 mA was applied for 48 h. Column chromatography on silica gel (*n*-hexane/ethyl acetate = 4:1) yielded **5** (1.42 g, 66%) as colourless solid.

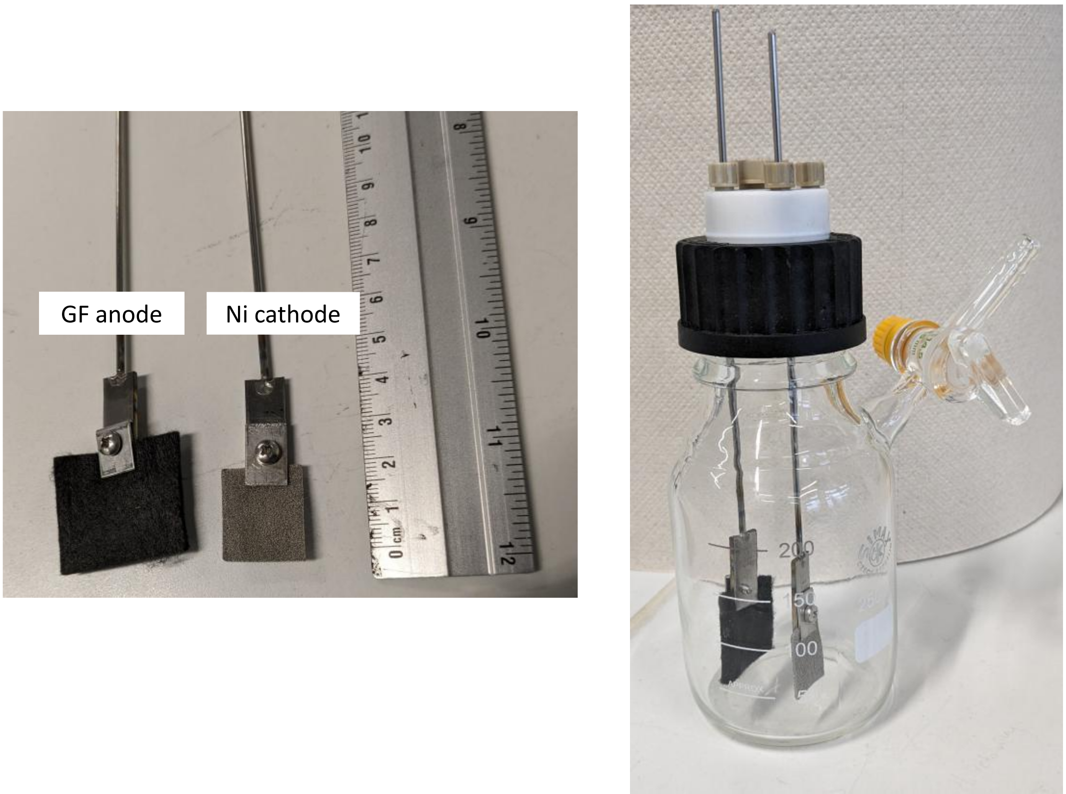

Figure S3. Photograph of bottle-type cell for large-scale reaction.

VII. CV Studies

CV Studies were performed using a Metrohm Autolab PGSTAT204 potentiostat and Nova 2.1 software. A glassy carbon work electrode (diameter: 3 mm), coiled platinum wire counter electrode, and a saturated calomel (SCE) reference were used. The voltammograms were recorded at room temperature in DMA (4 mL) with 0.1 M *n*Bu_4NPF_6 as supporting electrolyte under N_2 atmosphere. The scan rate is 100 mV/s.

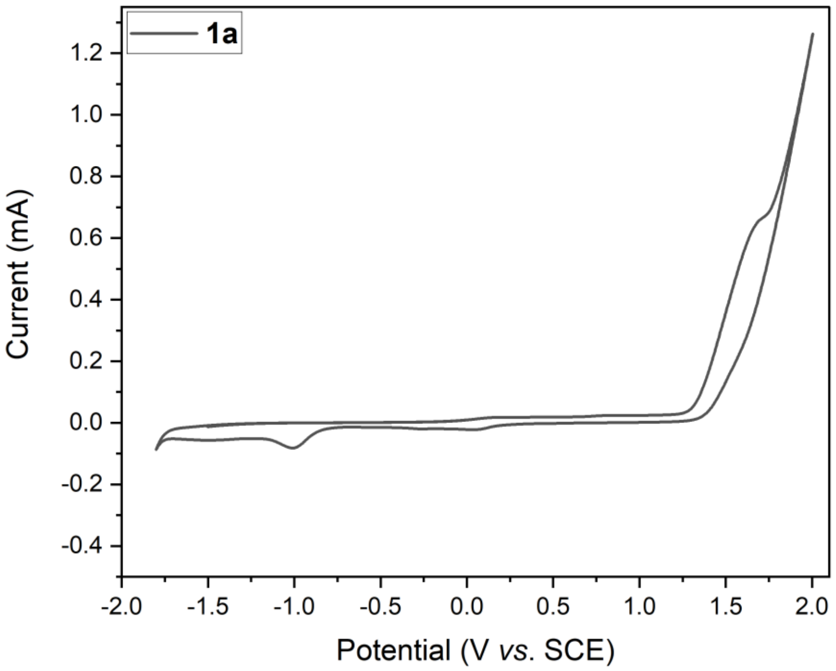

Figure S4. CV of **1a**.

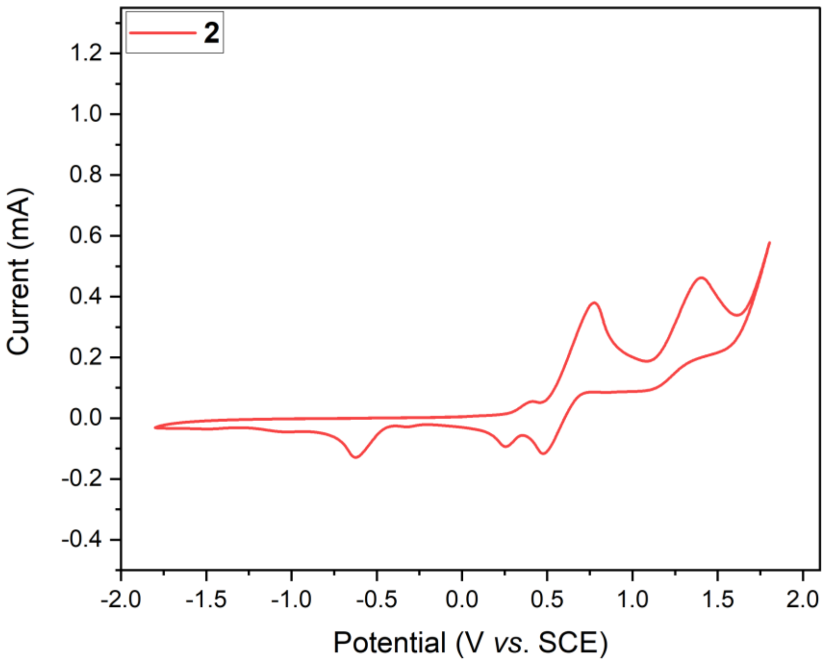

Figure S5. CV of **2**.

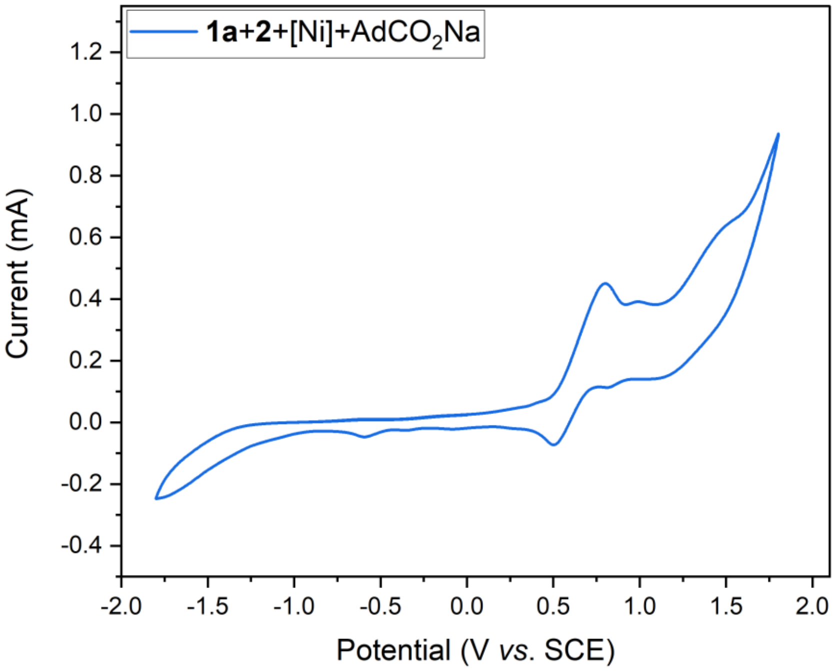

Figure S6. CV of *in situ* prepared Ni(II) complex **A**. The sample mixture was preheated at 140 °C for 30 min before measurement.

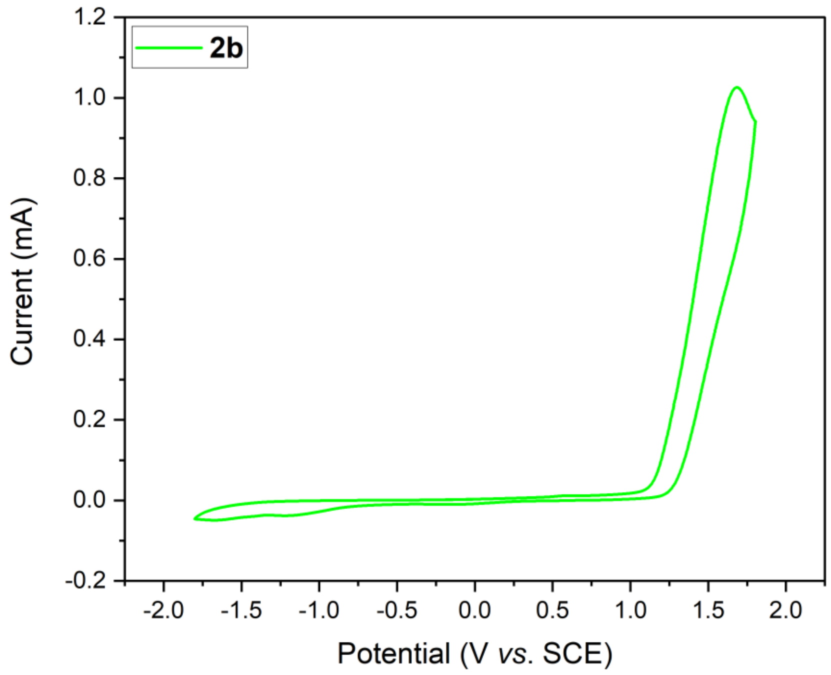

Figure S7. CV of pyrrole **2b**.

VIII. H/D Exchange

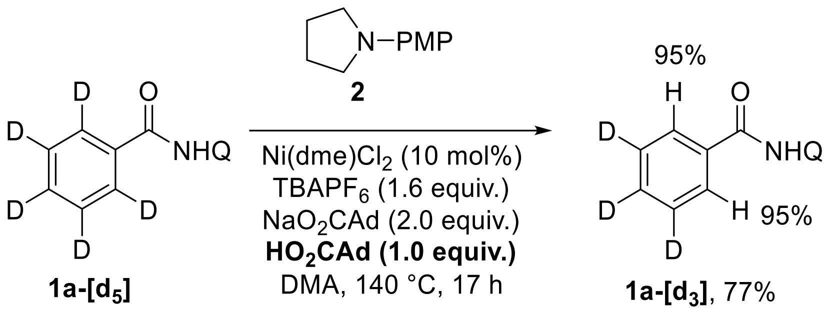

General Procedure **A**, without the use of electricity, was followed using benzamide **1a-[d$_5$]** (63.3 mg) and HO_2CAd (45.0 mg). Column chromatography on silica gel (*n*-hexane/ethyl acetate = 5:1) yielded **1a-[d$_3$]**(48.3 mg, 77%) as colourless solid.

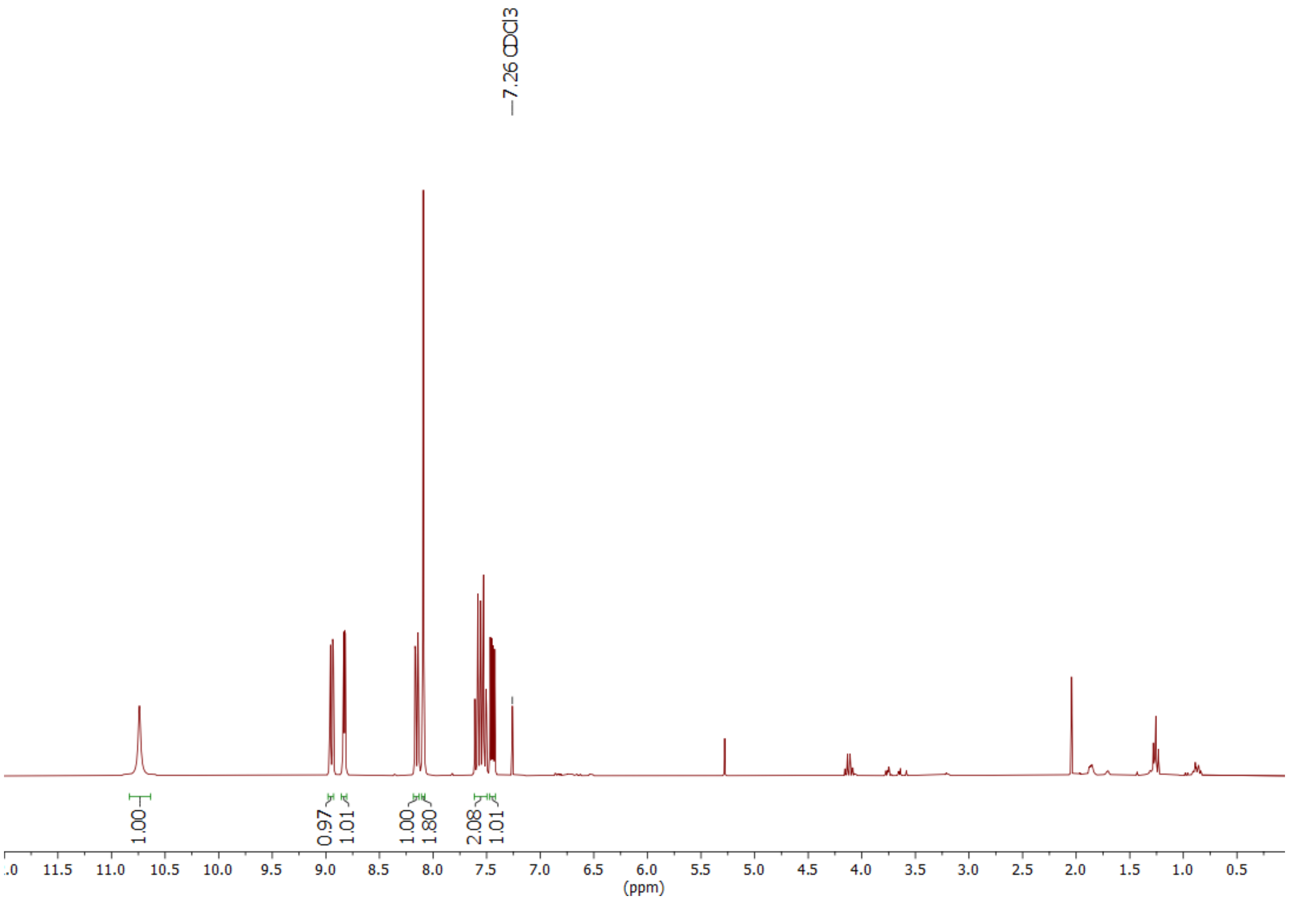

IX. Gas Measurement Studies

Qualitative analysis of hydrogen.

Initially, the standard reaction has been performed following the general procedure. The headspace of the reaction has been subsequently analyzed via gas chromatography. To that extend, a sample of the headspace was carefully collected using a gas syringe. The observed peak at a retention time of 1.56 min was assigned to molecular hydrogen as a reference sample of pure hydrogen gas shows the same retention time.

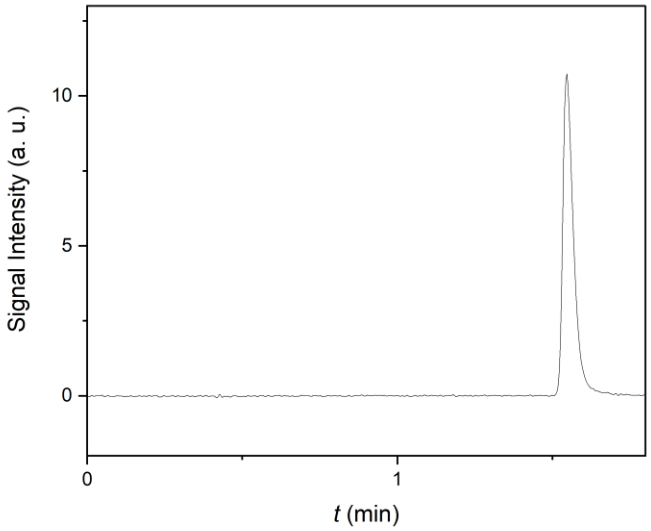

Figure S8: Qualitative analysis of hydrogen.

Analysis of change in headspace volume

The overall change of the headspace volume during the course of the reaction has been monitored using a GasMess-System (LIKAT Rostock and MesSen Nord GmbH) operating under isobaric conditions. Here, the reaction was performed following the general procedure.

Figure S9: Monitoring of change in headspace volume.

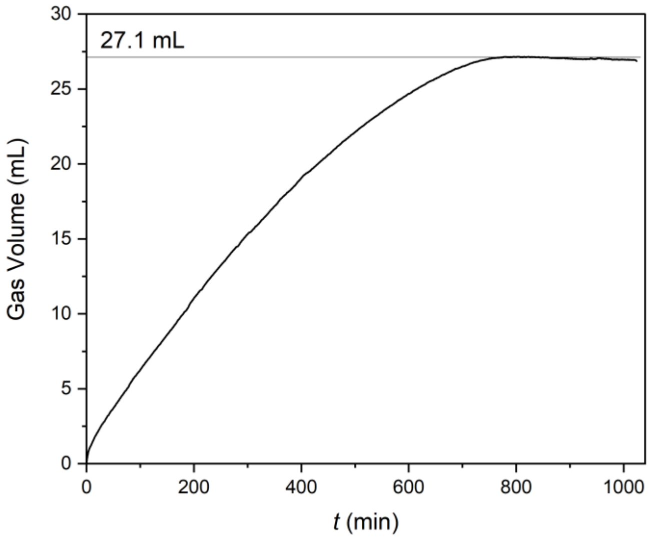

Quantitative analysis of hydrogen.

Additionally, a control experiment has been performed without current under otherwise same conditions.

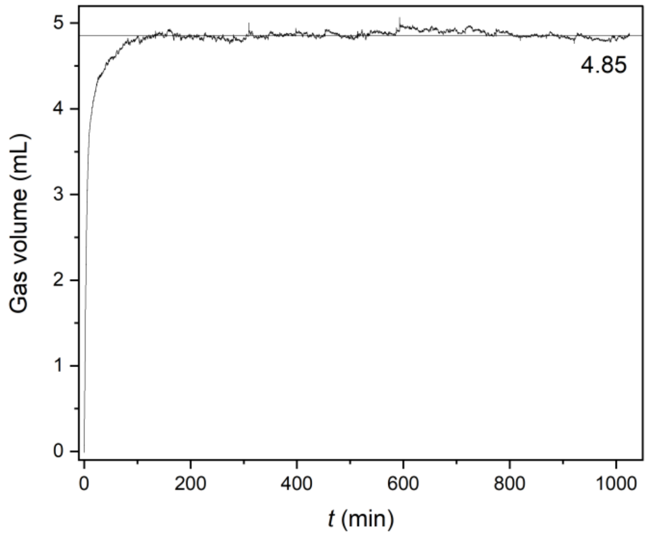

Figure S10: Monitoring of change in headspace volume without current.

X. Crystallographic Data of **8** and **29**

Data were collected from single crystals at 100.00 K on a Bruker D8 VENTURE dual wavelength Mo/Cu four-circle diffractometer equipped with a microfocus sealed X-ray tube using a mirror optics as monochromator and a Bruker PHOTON III detector. The diffractometer was equipped with an Oxford Cryostream 800 low temperature device and used MoK_α radiation (λ = 0.71073 Å). The data were integrated with SAINT and a multi-scan absorption correction using SADABS was applied.[11-12] The structure was solved by dual methods using XT and refined by full-matrix least-squares methods against F^2 by XL using Olex2.[13-15]

Crystallographic Data of **8** (CCDC: 2416895)

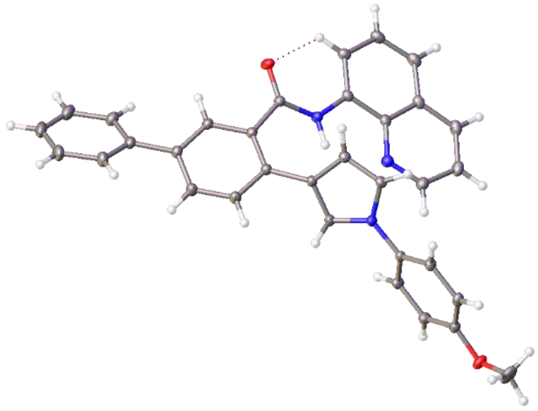

Figure S11: Structure of **8**.

Table S5. Crystal data and structure refinement for **8**.

CCDC number	2416895
Empirical formula	$C_{33}H_{25}N_3O_2$
Formula weight	495.56
Temperature [K]	100.00
Crystal system	monoclinic
Space group (number)	$P2_1/n$ (14)
a [Å]	9.2769(6)
b [Å]	28.1618(18)
c [Å]	10.1007(5)
α [°]	90
β [°]	110.935(2)
γ [°]	90
Volume [Å^3]	2464.6(3)
Z	4
ρ_{calc} [gcm^{-3}]	1.336
μ [mm^{-1}]	0.084
F(000)	1040
Crystal size [mm^3]	0.295×0.188×0.173
Crystal colour	colourless

Crystal shape	block
Radiation	MoK_α (λ=0.71073 Å)
2θ range [°]	4.55 to 63.22 (0.68 Å)
Index ranges	−13 ≤ h ≤ 13<br>−41 ≤ k ≤ 41<br>−11 ≤ l ≤ 14
Reflections collected	81517
Independent reflections	8220<br>R_{int} = 0.0391<br>R_{sigma} = 0.0180
Completeness to θ = 25.242°	100.0 %
Data / Restraints / Parameters	8220/1/348
Absorption correction T_{min}/T_{max} (method)	0.7514/1.0000 (multi-scan)
Goodness-of-fit on F^2	1.035
Final *R* indexes [I≥2σ(I)]	R_1 = 0.0416<br>wR_2 = 0.1111
Final *R* indexes [all data]	R_1 = 0.0469<br>wR_2 = 0.1164
Largest peak/hole [eÅ^{-3}]	0.47/−0.26

Table S6. Bond lengths [Å] and angles [°] for **8**.

**Atom–Atom**	**Length [Å]**
O1–C1	1.2275(10)
O2–C30	1.3623(10)
O2–C33	1.4289(13)
N1–H1	0.923(12)
N1–C1	1.3646(10)
N1–C18	1.4004(10)
N2–C19	1.3652(11)
N2–C26	1.3214(11)
N3–C15	1.3758(10)
N3–C17	1.3811(11)
N3–C27	1.4212(10)
C1–C2	1.5073(11)
C2–C3	1.4005(11)
C2–C13	1.4105(11)
C3–H3	0.9500
C3–C4	1.3982(12)
C4–C5	1.4838(12)
C4–C11	1.4028(12)
C5–C6	1.3992(13)
C5–C10	1.4008(13)
C6–H6	0.9500
C6–C7	1.3915(13)
C7–H7	0.9500
C7–C8	1.3918(16)
C8–H8	0.9500
C8–C9	1.3851(16)
C9–H9	0.9500
C9–C10	1.3906(13)
C10–H10	0.9500
C11–H11	0.9500
C11–C12	1.3849(12)
C12–H12	0.9500
C12–C13	1.4015(12)
C13–C14	1.4793(11)
C14–C15	1.3795(11)
C14–C16	1.4306(12)
C15–H15	0.9500
C16–H16	0.9500
C16–C17	1.3733(12)
C17–H17	0.9500
C18–C19	1.4365(11)
C18–C23	1.3822(11)
C19–C20	1.4184(11)
C20–C21	1.4160(13)
C20–C24	1.4185(13)
C21–H21	0.9500
C21–C22	1.3709(13)
C22–H22	0.9500
C22–C23	1.4158(12)
C23–H23	0.9500
C24–H24	0.9500

C24–C25	1.3684(14)
C25–H25	0.9500
C25–C26	1.4125(13)
C26–H26	0.9500
C27–C28	1.3881(11)
C27–C32	1.3967(12)
C28–H28	0.9500
C28–C29	1.3955(12)
C29–H29	0.9500
C29–C30	1.3944(13)
C30–C31	1.4009(12)
C31–H31	0.9500
C31–C32	1.3847(12)
C32–H32	0.9500
C33–H33A	0.9800
C33–H33B	0.9800
C33–H33C	0.9800

**Atom–Atom–Atom**	**Angle [°]**
C30–O2–C33	117.43(8)
C1–N1–H1	119.1(9)
C1–N1–C18	127.88(7)
C18–N1–H1	112.8(9)
C26–N2–C19	117.54(8)
C15–N3–C17	108.81(7)
C15–N3–C27	125.06(7)
C17–N3–C27	126.08(7)
O1–C1–N1	122.95(8)
O1–C1–C2	121.09(8)
N1–C1–C2	115.81(7)
C3–C2–C1	114.39(7)
C3–C2–C13	119.94(7)
C13–C2–C1	125.61(7)
C2–C3–H3	118.9
C4–C3–C2	122.14(8)
C4–C3–H3	118.9
C3–C4–C5	122.34(8)
C3–C4–C11	117.33(8)
C11–C4–C5	120.34(8)
C6–C5–C4	121.90(8)
C6–C5–C10	118.26(8)
C10–C5–C4	119.82(8)
C5–C6–H6	119.6
C7–C6–C5	120.74(9)
C7–C6–H6	119.6
C6–C7–H7	119.9
C6–C7–C8	120.13(10)
C8–C7–H7	119.9
C7–C8–H8	120.1
C9–C8–C7	119.83(9)
C9–C8–H8	120.1
C8–C9–H9	120.0
C8–C9–C10	120.05(10)
C10–C9–H9	120.0
C5–C10–H10	119.5
C9–C10–C5	120.97(9)
C9–C10–H10	119.5
C4–C11–H11	119.5
C12–C11–C4	120.97(8)
C12–C11–H11	119.5
C11–C12–H12	119.0
C11–C12–C13	121.96(8)
C13–C12–H12	119.0
C2–C13–C14	125.33(7)
C12–C13–C2	117.54(7)
C12–C13–C14	117.13(7)
C15–C14–C13	124.55(8)
C15–C14–C16	106.79(7)
C16–C14–C13	128.52(8)
N3–C15–C14	108.61(7)
N3–C15–H15	125.7
C14–C15–H15	125.7
C14–C16–H16	126.3
C17–C16–C14	107.49(7)
C17–C16–H16	126.3
N3–C17–H17	125.8
C16–C17–N3	108.30(7)
C16–C17–H17	125.8
N1–C18–C19	114.58(7)
C23–C18–N1	125.86(8)
C23–C18–C19	119.55(8)
N2–C19–C18	117.34(7)
N2–C19–C20	123.02(8)
C20–C19–C18	119.63(8)
C19–C20–C24	117.10(8)
C21–C20–C19	119.51(8)
C21–C20–C24	123.39(8)
C20–C21–H21	120.2
C22–C21–C20	119.64(8)
C22–C21–H21	120.2
C21–C22–H22	119.0
C21–C22–C23	121.91(8)
C23–C22–H22	119.0
C18–C23–C22	119.73(8)
C18–C23–H23	120.1
C22–C23–H23	120.1
C20–C24–H24	120.2
C25–C24–C20	119.59(8)
C25–C24–H24	120.2

C24–C25–H25	120.6
C24–C25–C26	118.80(8)
C26–C25–H25	120.6
N2–C26–C25	123.93(9)
N2–C26–H26	118.0
C25–C26–H26	118.0
C28–C27–N3	120.31(7)
C28–C27–C32	119.49(8)
C32–C27–N3	120.19(7)
C27–C28–H28	119.6
C27–C28–C29	120.84(8)
C29–C28–H28	119.6
C28–C29–H29	120.3
C30–C29–C28	119.43(8)
C30–C29–H29	120.3
O2–C30–C29	124.86(8)
O2–C30–C31	115.34(8)
C29–C30–C31	119.79(8)
C30–C31–H31	119.9
C32–C31–C30	120.25(8)
C32–C31–H31	119.9
C27–C32–H32	119.9
C31–C32–C27	120.17(8)
C31–C32–H32	119.9
O2–C33–H33A	109.5
O2–C33–H33B	109.5
O2–C33–H33C	109.5
H33A–C33–H33B	109.5
H33A–C33–H33C	109.5
H33B–C33–H33C	109.5

Crystallographic Data of **29** (CCDC: 2416896)

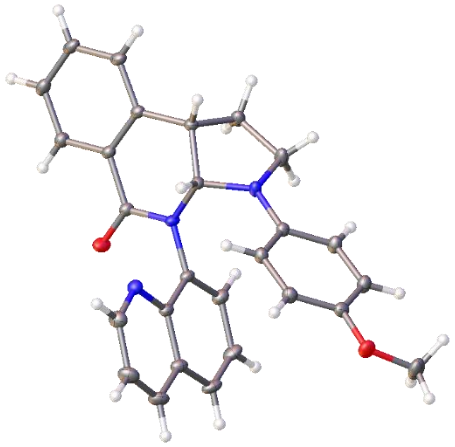

Figure S12: Structure of **29**.

Table S7. Crystal data and structure refinement for **29**

CCDC number	2416896
Empirical formula	$C_{27}H_{23}N_3O_2$
Formula weight	421.48
Temperature [K]	100.00
Crystal system	monoclinic
Space group (number)	$P2_1/c$ (14)
a [Å]	9.0721(9)
b [Å]	27.381(3)
c [Å]	8.7834(7)
α [°]	90
β [°]	107.896(3)
γ [°]	90
Volume [Å^3]	2076.3(3)
Z	4
ρ_{calc} [gcm^{-3}]	1.348
μ [mm^{-1}]	0.086
F(000)	888
Crystal size [mm^3]	0.5×0.341×0.221
Crystal colour	yellow

Crystal shape	block
Radiation	MoK_{α} (λ=0.71073 Å)
2θ range [°]	4.72 to 61.02 (0.70 Å)
Index ranges	$-12 \leq h \leq 12$
	$0 \leq k \leq 39$
	$0 \leq l \leq 12$
Reflections collected	6311
Independent reflections	6311
	R_{int} = 0.0472
	R_{sigma} = 0.0253
Completeness to	99.8 %
θ = 25.242°	
Data / Restraints / Parameters	6311/0/290
Absorption correction	0.635898/0.874934
T_{min}/T_{max} (method)	(multi-scan)
Goodness-of-fit on F^2	1.049
Final R indexes	R_1 = 0.0443
[$I \geq 2\sigma(I)$]	wR_2 = 0.1217
Final R indexes	R_1 = 0.0467
[all data]	wR_2 = 0.1236
Largest peak/hole [eÅ^{-3}]	0.45/−0.26

Table S8. Bond lengths [Å] and angles [°] for **29**.

**Atom–Atom**	**Length [Å]**		
O1–C1	1.2355(12)	C1–C2	1.4903(12)
O2–C24	1.3794(11)	C2–C3	1.3958(13)
O2–C27	1.4229(15)	C2–C11	1.3969(13)
N1–C1	1.3686(12)	C3–C4	1.5010(13)
N1–C5	1.4980(11)	C3–C8	1.4007(12)
N1–C12	1.4349(11)	C4–H4	1.0000
N2–C5	1.4393(12)	C4–C5	1.5295(12)
N2–C7	1.4598(12)	C4–C6	1.5472(14)
N2–C21	1.3982(12)	C5–H5	1.0000
N3–C13	1.3693(12)	C6–H6A	0.9900
N3–C17	1.3203(13)	C6–H6B	0.9900

C6–C7	1.5317(14)
C7–H7A	0.9900
C7–H7B	0.9900
C8–H8	0.9500
C8–C9	1.3927(14)
C9–H9	0.9500
C9–C10	1.3952(15)
C10–H10	0.9500
C10–C11	1.3918(13)
C11–H11	0.9500
C12–C13	1.4233(13)
C12–C20	1.3712(13)
C13–C14	1.4231(12)
C14–C15	1.4157(14)
C14–C18	1.4207(14)
C15–H15	0.9500
C15–C16	1.3734(16)
C16–H16	0.9500
C16–C17	1.4113(15)
C17–H17	0.9500
C18–H18	0.9500
C18–C19	1.3680(16)
C19–H19	0.9500
C19–C20	1.4185(14)
C20–H20	0.9500
C21–C22	1.4082(13)
C21–C26	1.3985(13)
C22–H22	0.9500
C22–C23	1.3875(13)
C23–H23	0.9500
C23–C24	1.3993(14)
C24–C25	1.3869(14)
C25–H25	0.9500
C25–C26	1.3977(14)
C26–H26	0.9500
C27–H27A	0.9800
C27–H27B	0.9800
C27–H27C	0.9800

**Atom–Atom–Atom**	**Angle [°]**
C24–O2–C27	116.22(9)
C1–N1–C5	124.15(7)
C1–N1–C12	115.56(8)
C12–N1–C5	118.14(7)
C5–N2–C7	111.28(7)
C21–N2–C5	125.45(8)
C21–N2–C7	121.15(8)
C17–N3–C13	116.92(9)
O1–C1–N1	121.58(8)
O1–C1–C2	121.07(8)
N1–C1–C2	117.33(8)
C3–C2–C1	121.19(8)
C3–C2–C11	120.74(8)
C11–C2–C1	118.07(8)
C2–C3–C4	119.64(8)
C2–C3–C8	118.88(9)
C8–C3–C4	121.30(8)
C3–C4–H4	109.1
C3–C4–C5	115.22(8)
C3–C4–C6	111.60(8)
C5–C4–H4	109.1
C5–C4–C6	102.56(7)
C6–C4–H4	109.1
N1–C5–C4	110.13(7)
N1–C5–H5	110.6
N2–C5–N1	112.18(7)
N2–C5–C4	102.31(7)
N2–C5–H5	110.6
C4–C5–H5	110.6
C4–C6–H6A	110.8
C4–C6–H6B	110.8

H6A–C6–H6B	108.8	C17–C16–H16	120.7
C7–C6–C4	104.95(8)	N3–C17–C16	124.80(10)
C7–C6–H6A	110.8	N3–C17–H17	117.6
C7–C6–H6B	110.8	C16–C17–H17	117.6
N2–C7–C6	104.58(8)	C14–C18–H18	119.8
N2–C7–H7A	110.8	C19–C18–C14	120.35(9)
N2–C7–H7B	110.8	C19–C18–H18	119.8
C6–C7–H7A	110.8	C18–C19–H19	119.8
C6–C7–H7B	110.8	C18–C19–C20	120.49(9)
H7A–C7–H7B	108.9	C20–C19–H19	119.8
C3–C8–H8	119.7	C12–C20–C19	120.34(9)
C9–C8–C3	120.56(9)	C12–C20–H20	119.8
C9–C8–H8	119.7	C19–C20–H20	119.8
C8–C9–H9	120.0	N2–C21–C22	122.23(8)
C8–C9–C10	120.03(9)	N2–C21–C26	120.29(8)
C10–C9–H9	120.0	C26–C21–C22	117.43(9)
C9–C10–H10	120.0	C21–C22–H22	119.6
C11–C10–C9	119.93(9)	C23–C22–C21	120.86(9)
C11–C10–H10	120.0	C23–C22–H22	119.6
C2–C11–H11	120.1	C22–C23–H23	119.7
C10–C11–C2	119.84(9)	C22–C23–C24	120.68(9)
C10–C11–H11	120.1	C24–C23–H23	119.7
C13–C12–N1	119.80(8)	O2–C24–C23	116.02(9)
C20–C12–N1	119.57(9)	O2–C24–C25	124.55(9)
C20–C12–C13	120.60(8)	C25–C24–C23	119.42(9)
N3–C13–C12	118.58(8)	C24–C25–H25	120.2
N3–C13–C14	122.72(9)	C24–C25–C26	119.61(9)
C12–C13–C14	118.69(8)	C26–C25–H25	120.2
C15–C14–C13	117.87(9)	C21–C26–H26	119.0
C15–C14–C18	122.67(9)	C25–C26–C21	121.99(9)
C18–C14–C13	119.46(9)	C25–C26–H26	119.0
C14–C15–H15	120.5	O2–C27–H27A	109.5
C16–C15–C14	118.95(9)	O2–C27–H27B	109.5
C16–C15–H15	120.5	O2–C27–H27C	109.5
C15–C16–H16	120.7	H27A–C27–H27B	109.5
C15–C16–C17	118.70(10)	H27A–C27–H27C	109.5

H27B–C27–H27C	109.5

XI. ^{1}H and ^{13}C-NMR Spectra of Compounds

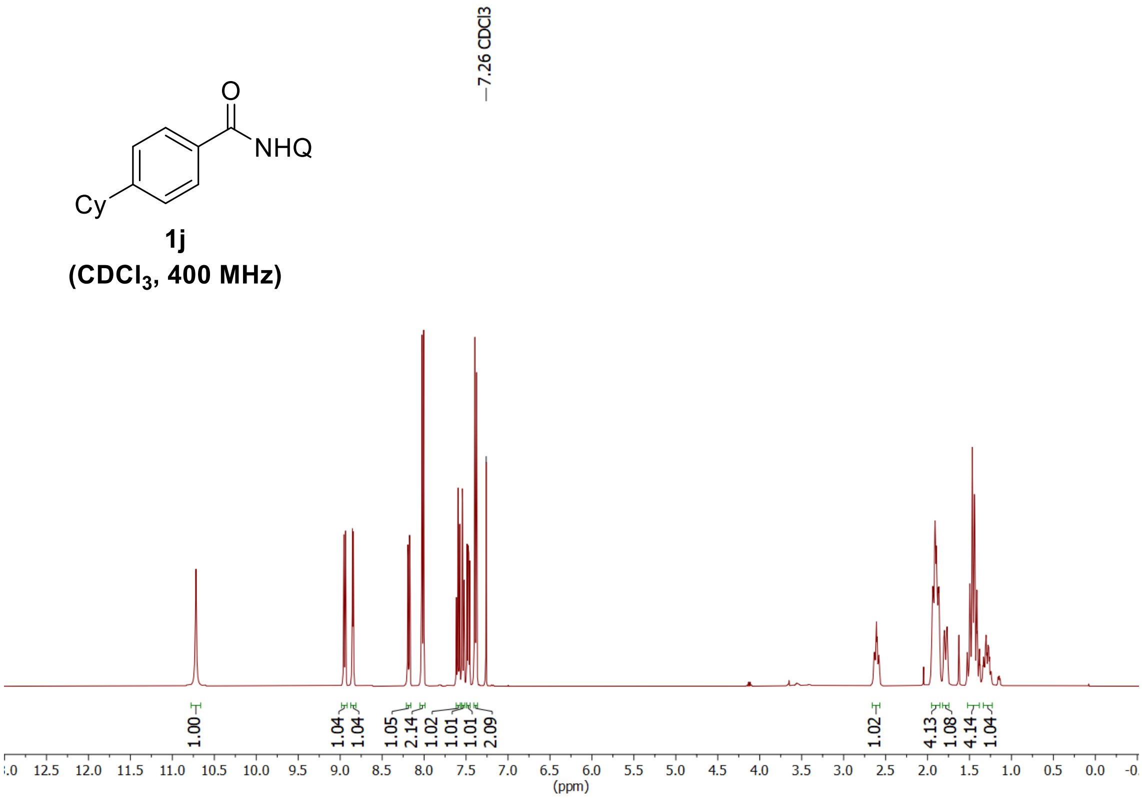

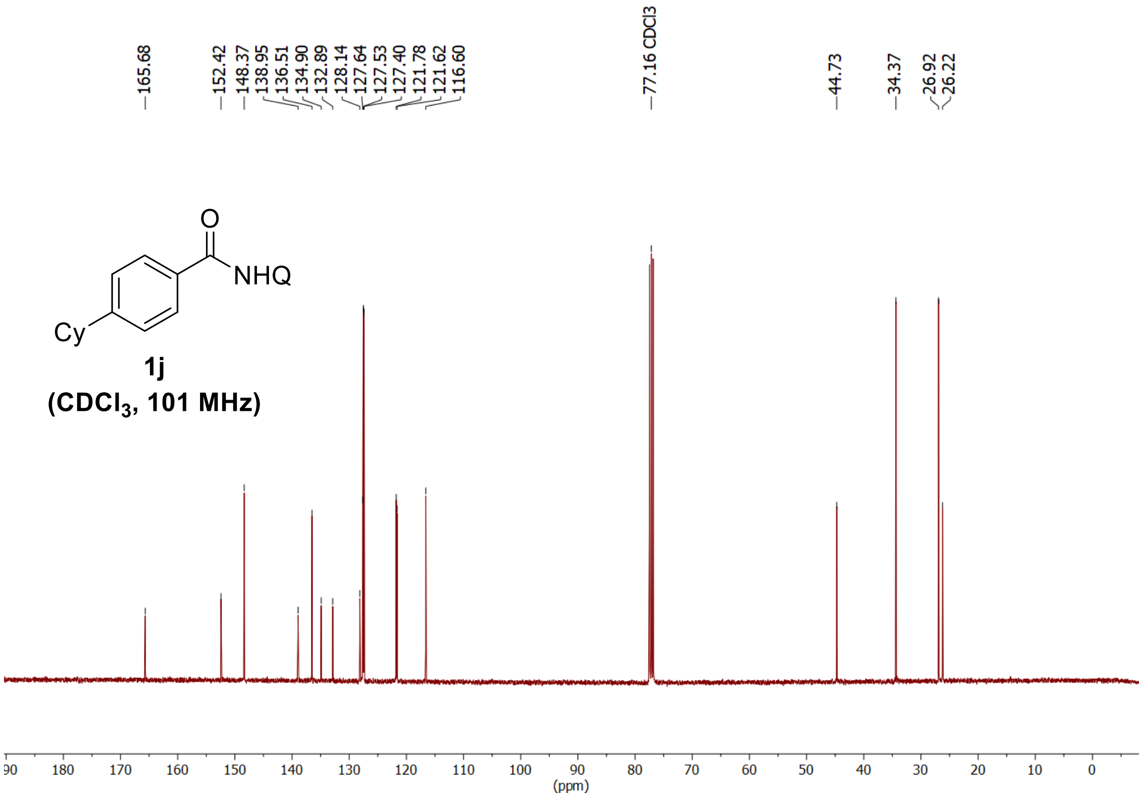

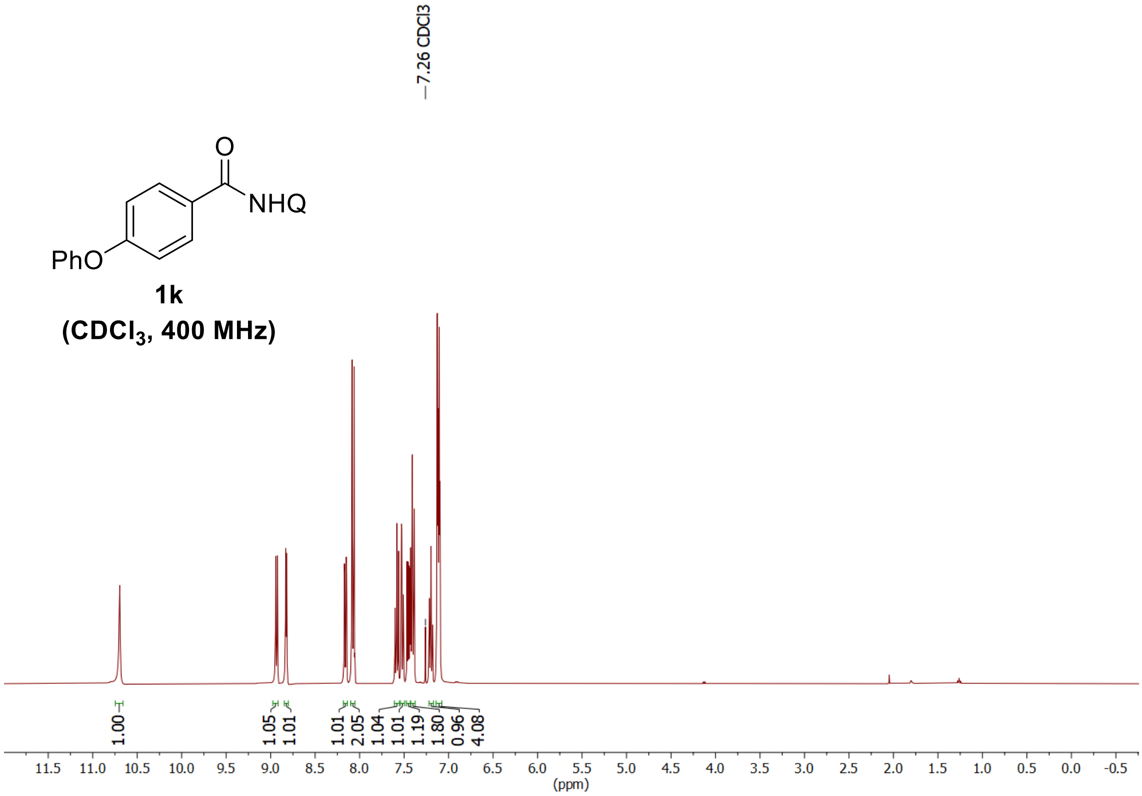

1k
($CDCl_3$, 400 MHz)

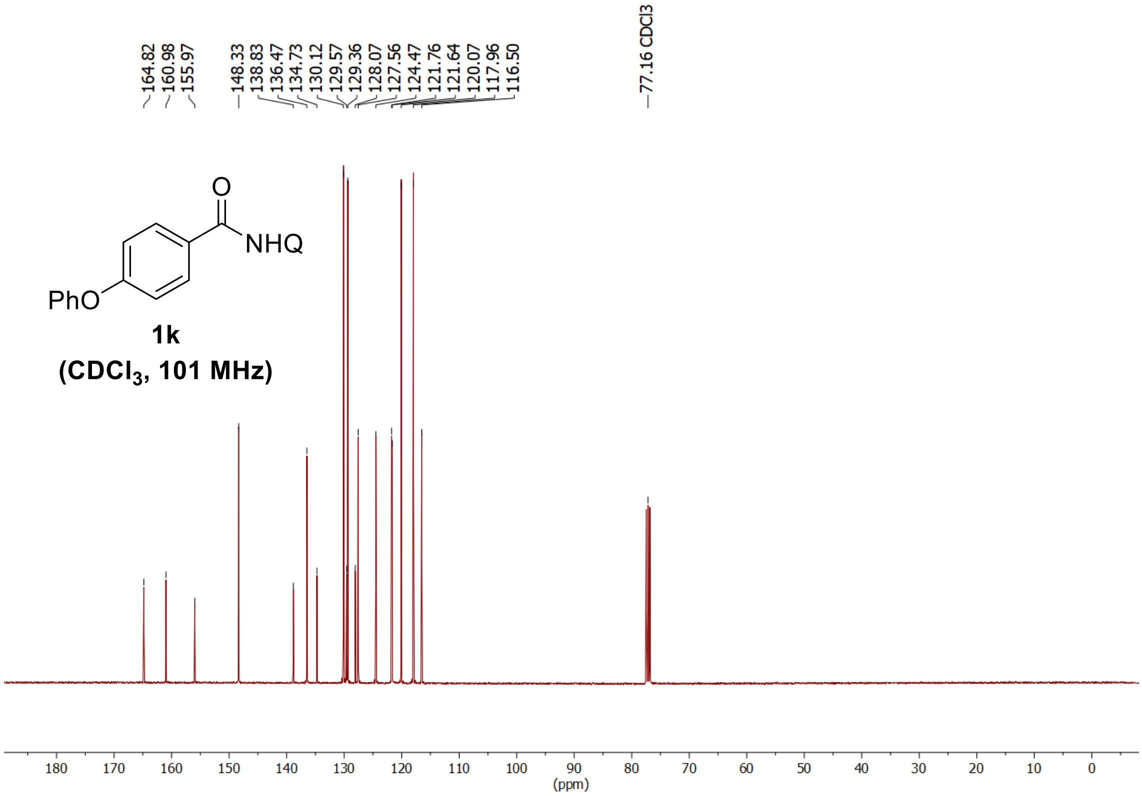

1k
($CDCl_3$, 101 MHz)

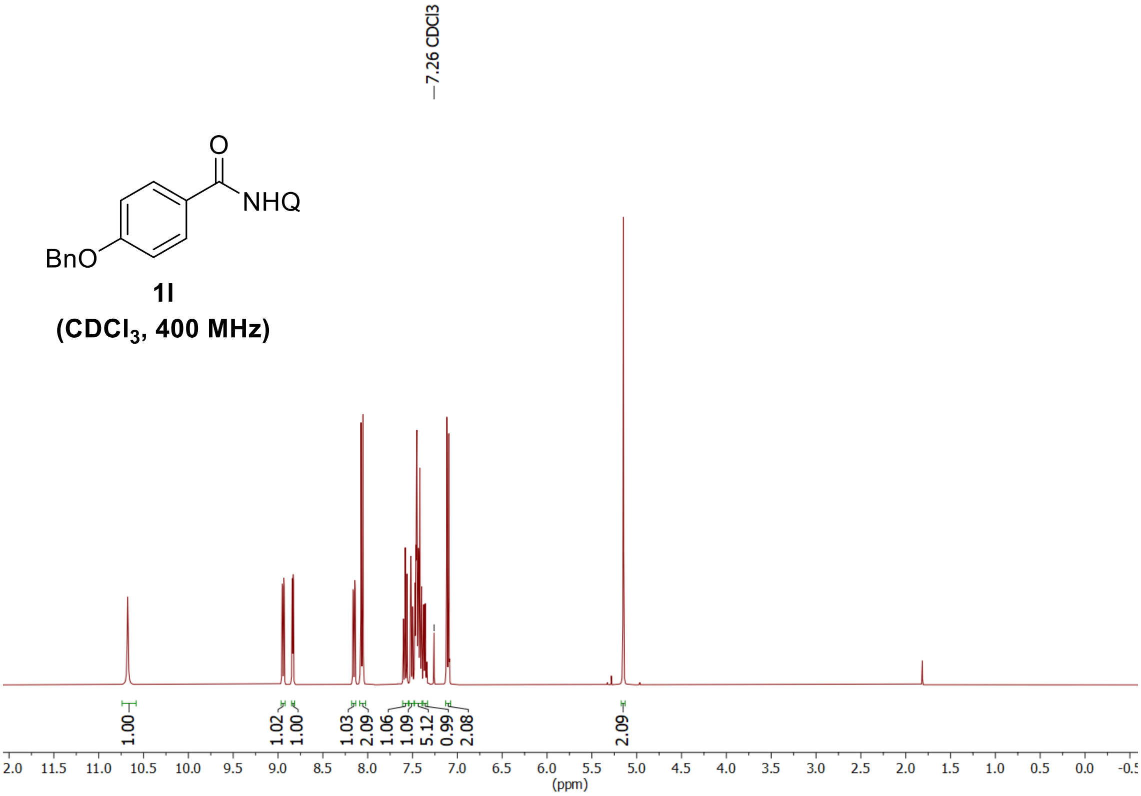
7.26 CDCl3
NHQ
BnO
1l
(CDCl3, 400 MHz)
1.00
1.02
1.00
1.03
2.09
1.06
1.09
5.12
0.99
2.08
2.09
(ppm)

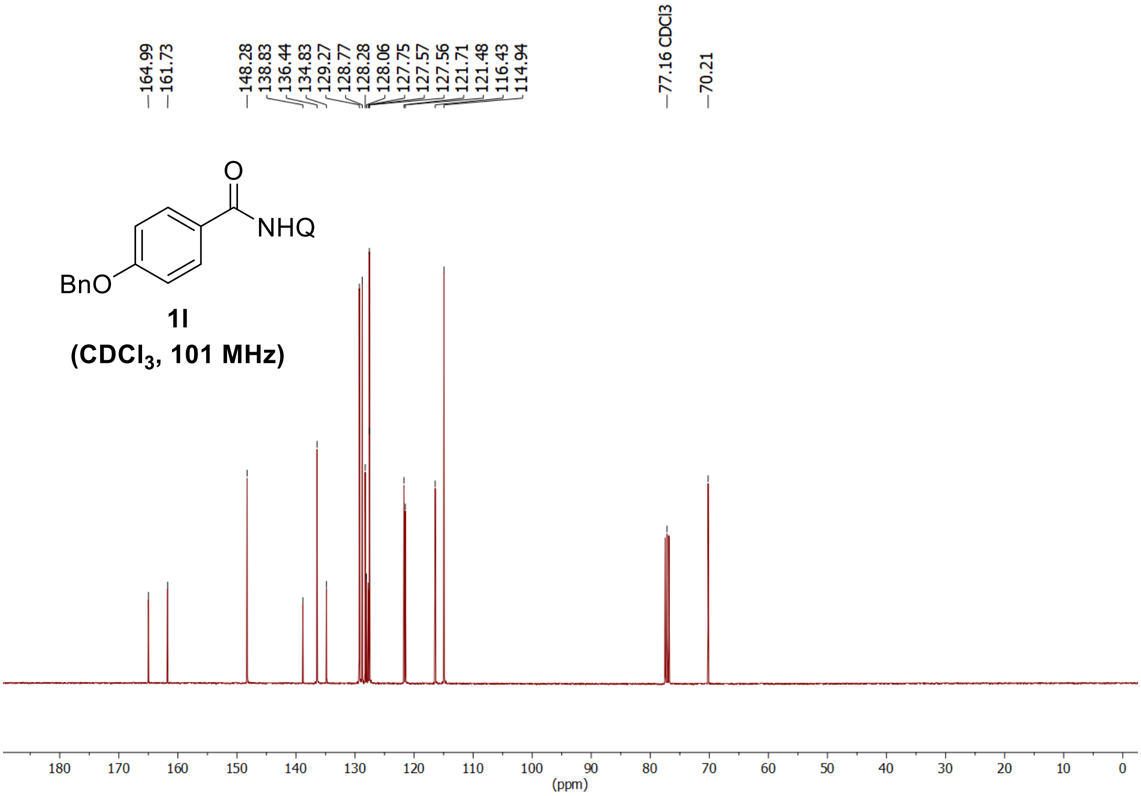
164.99
161.73
148.28
138.83
136.44
134.83
129.27
128.77
128.28
128.06
127.75
127.57
127.56
121.71
121.48
116.43
114.94
77.16 CDCl3
70.21
NHQ
BnO
1l
(CDCl3, 101 MHz)
(ppm)

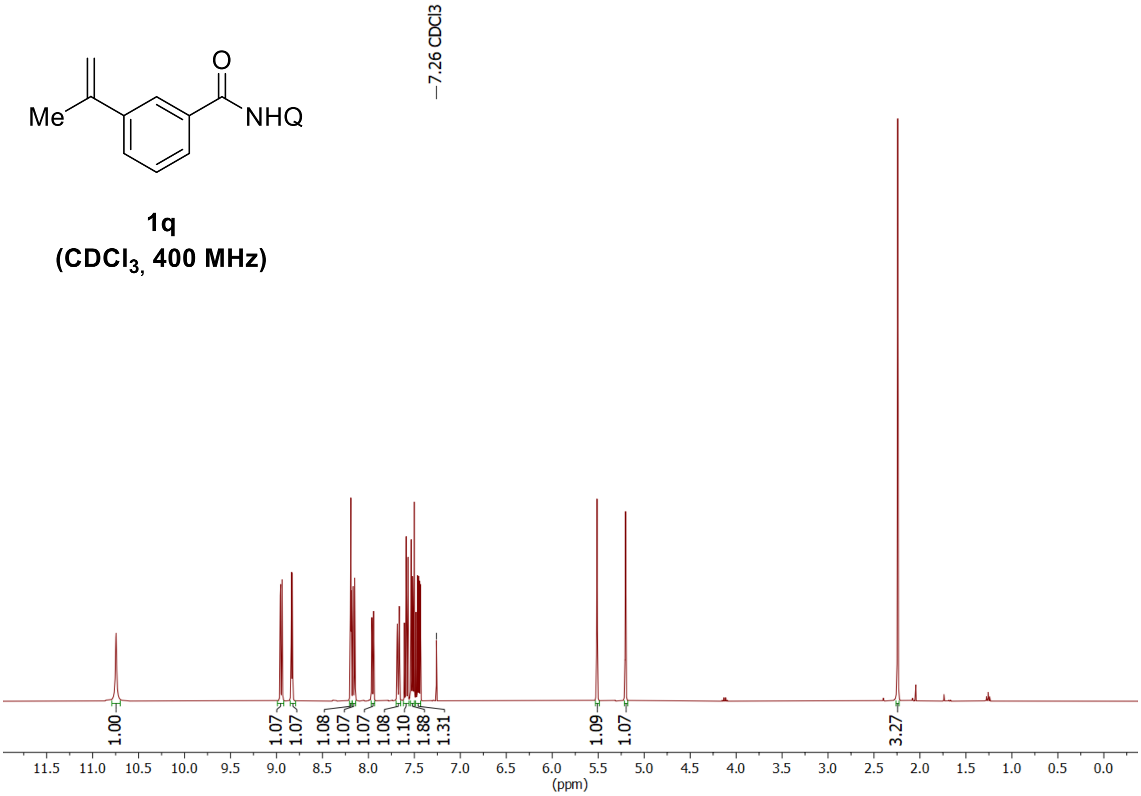

7.26 CDCl3
Me
O
NHQ
1q
(CDCl3, 400 MHz)
1.00
1.07
1.07
1.08
1.07
1.07
1.08
1.10
1.88
1.31
1.09
1.07
3.27
(ppm)

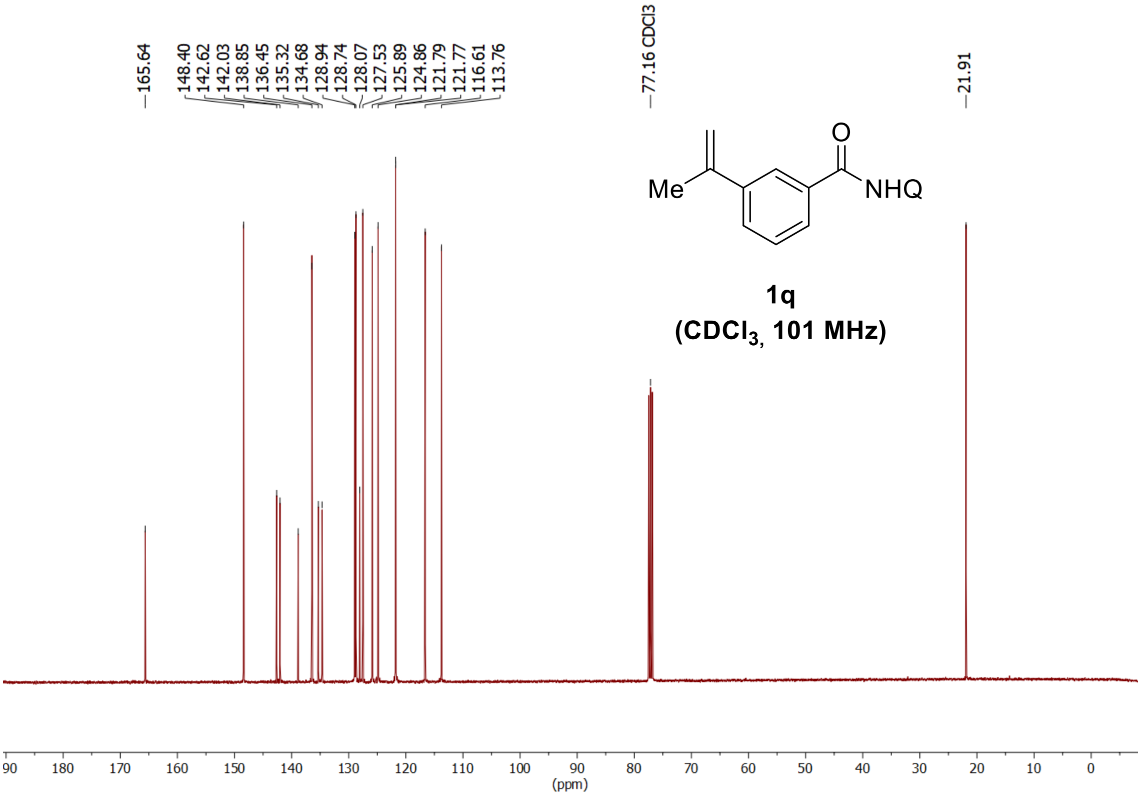

165.64
148.40
142.62
142.03
138.85
136.45
135.32
134.68
128.94
128.74
128.07
127.53
125.89
124.86
121.79
121.77
116.61
113.76
77.16 CDCl3
21.91
Me
O
NHQ
1q
(CDCl3, 101 MHz)
(ppm)

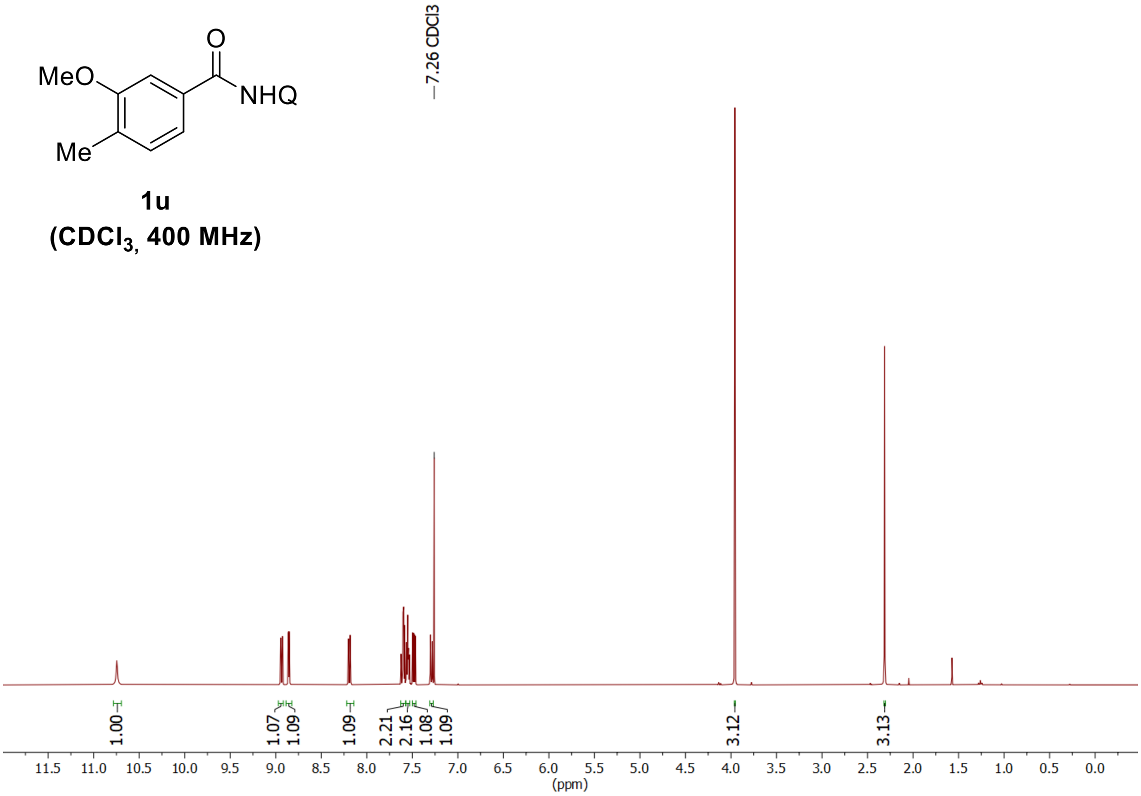
MeO
Me
O
NHQ
1u
(CDCl$_3$, 400 MHz)
7.26 CDCl3
1.00
1.07
1.09
1.09
2.21
2.16
1.08
1.09
3.12
3.13
(ppm)

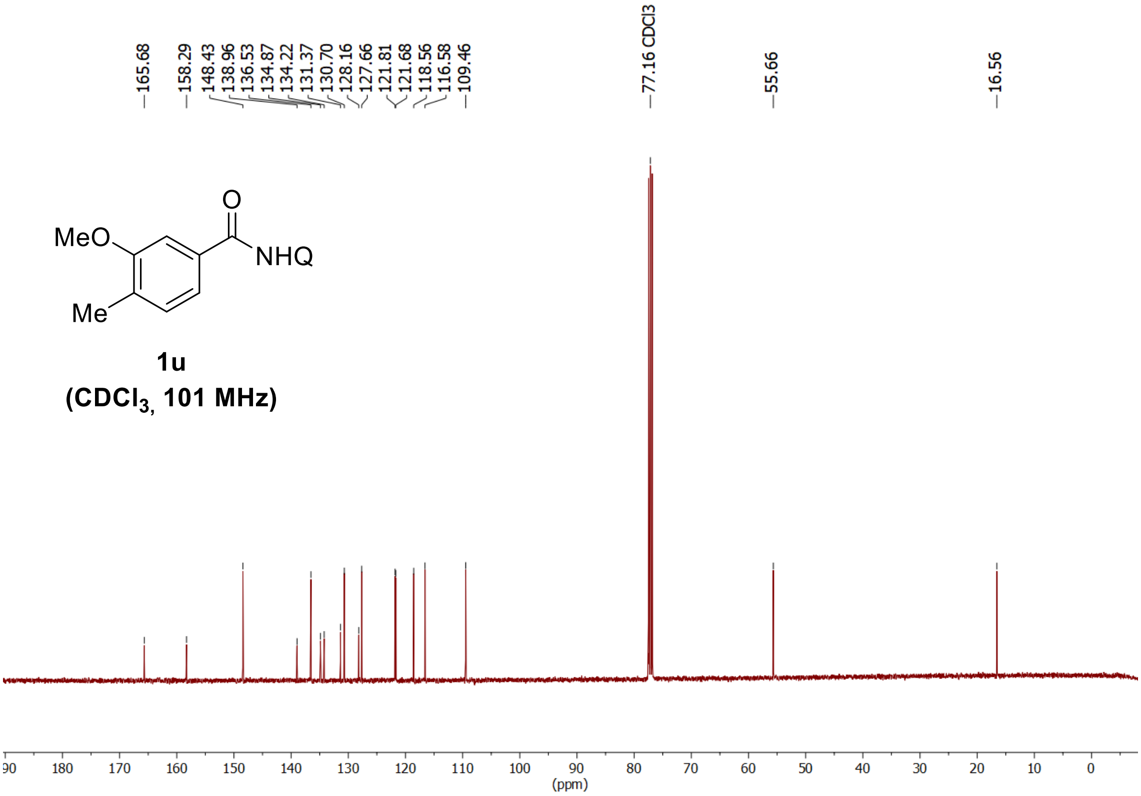
165.68
158.29
148.43
138.96
136.53
134.87
134.22
131.37
130.70
128.16
127.66
121.81
121.68
118.56
116.58
109.46
77.16 CDCl3
55.66
16.56
MeO
Me
O
NHQ
1u
(CDCl$_3$, 101 MHz)
(ppm)

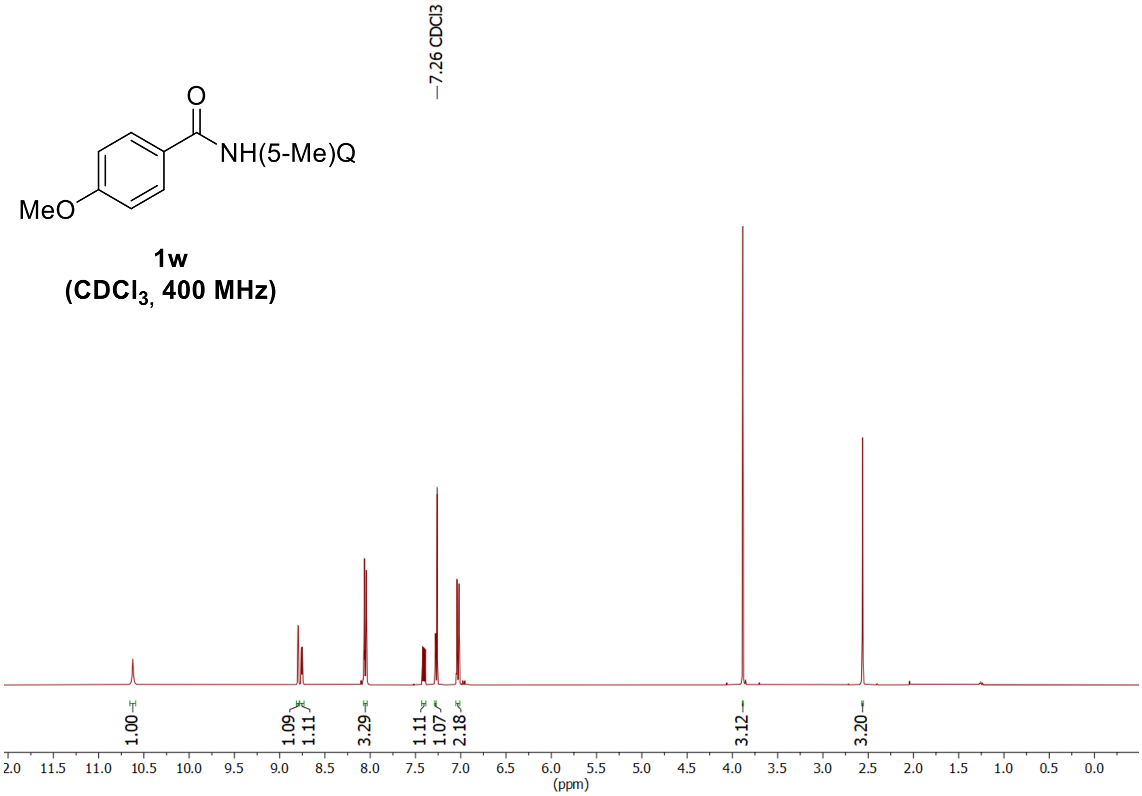
7.26 CDCl3
NH(5-Me)Q
MeO
O
1w
(CDCl3, 400 MHz)
1.00
1.09
1.11
3.29
1.11
1.07
2.18
3.12
3.20
(ppm)

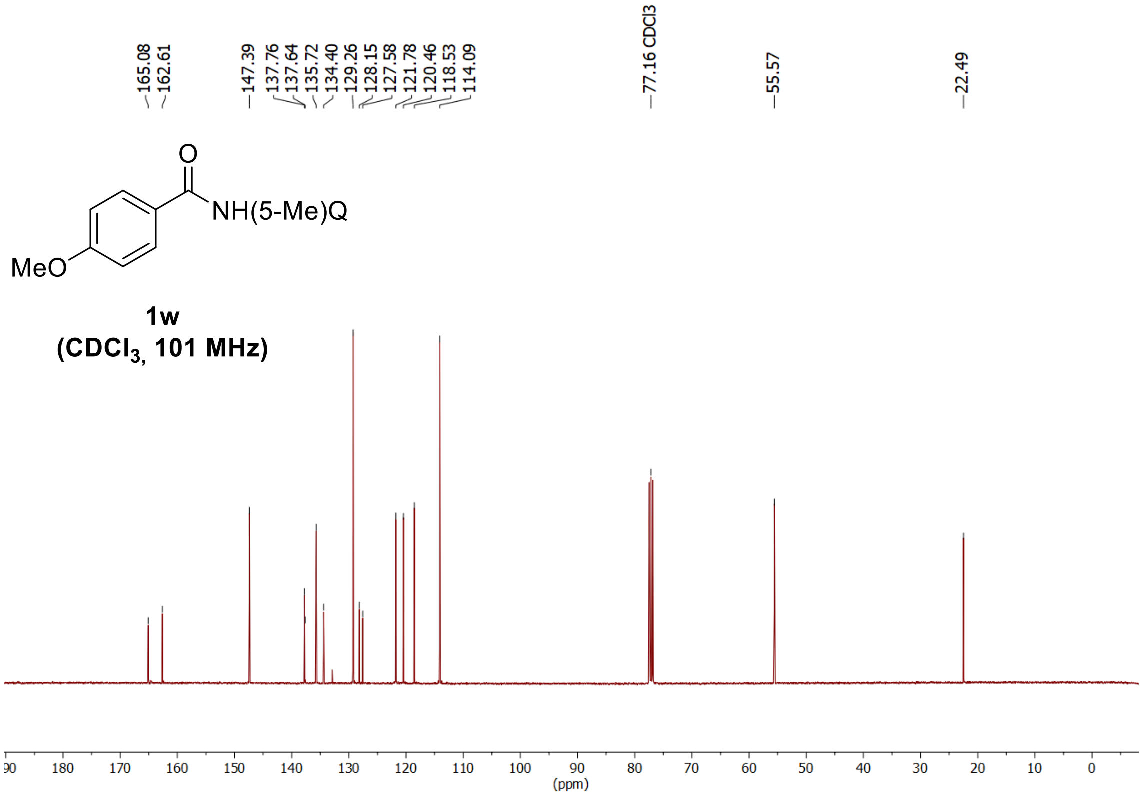
165.08
162.61
147.39
137.76
137.64
135.72
134.40
129.26
128.15
127.58
121.78
120.46
118.53
114.09
77.16 CDCl3
55.57
22.49
NH(5-Me)Q
MeO
O
1w
(CDCl3, 101 MHz)
(ppm)

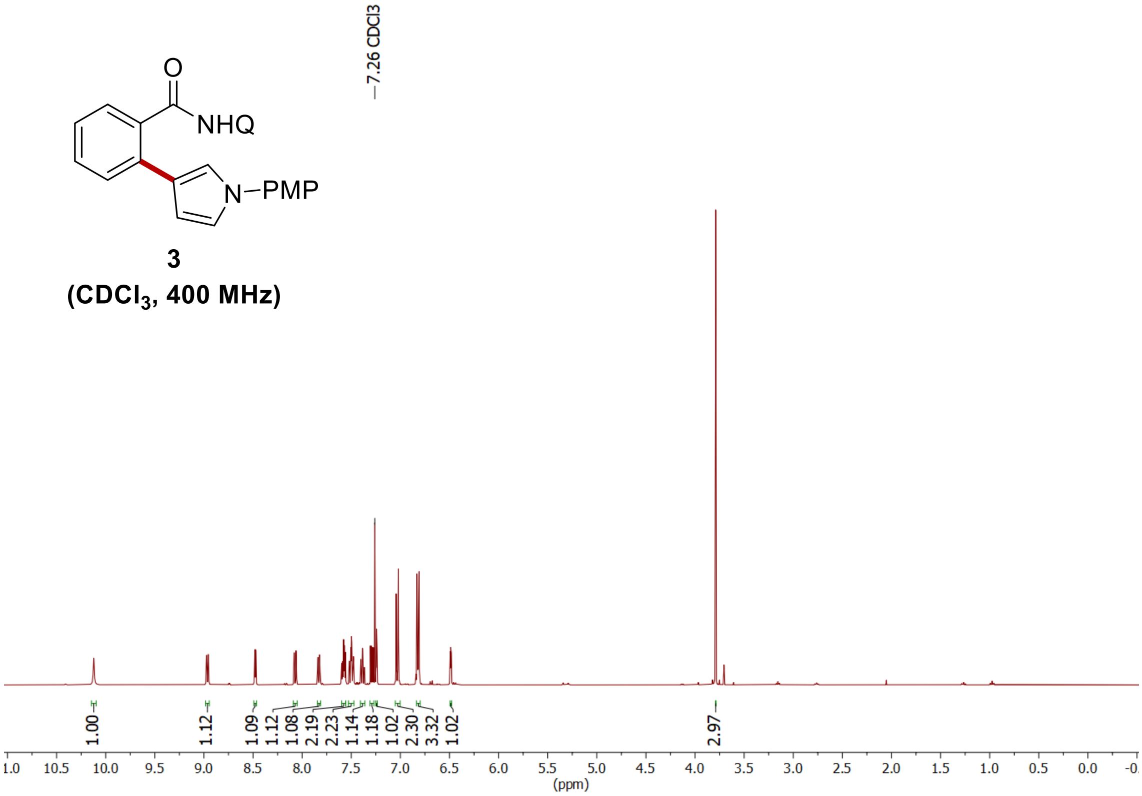

7.26 CDCl3
O
NHQ
N–PMP
3
(CDCl$_3$, 400 MHz)
1.00
1.12
1.09
1.12
1.08
2.19
2.23
1.14
1.18
1.02
2.30
3.32
1.02
2.97
(ppm)

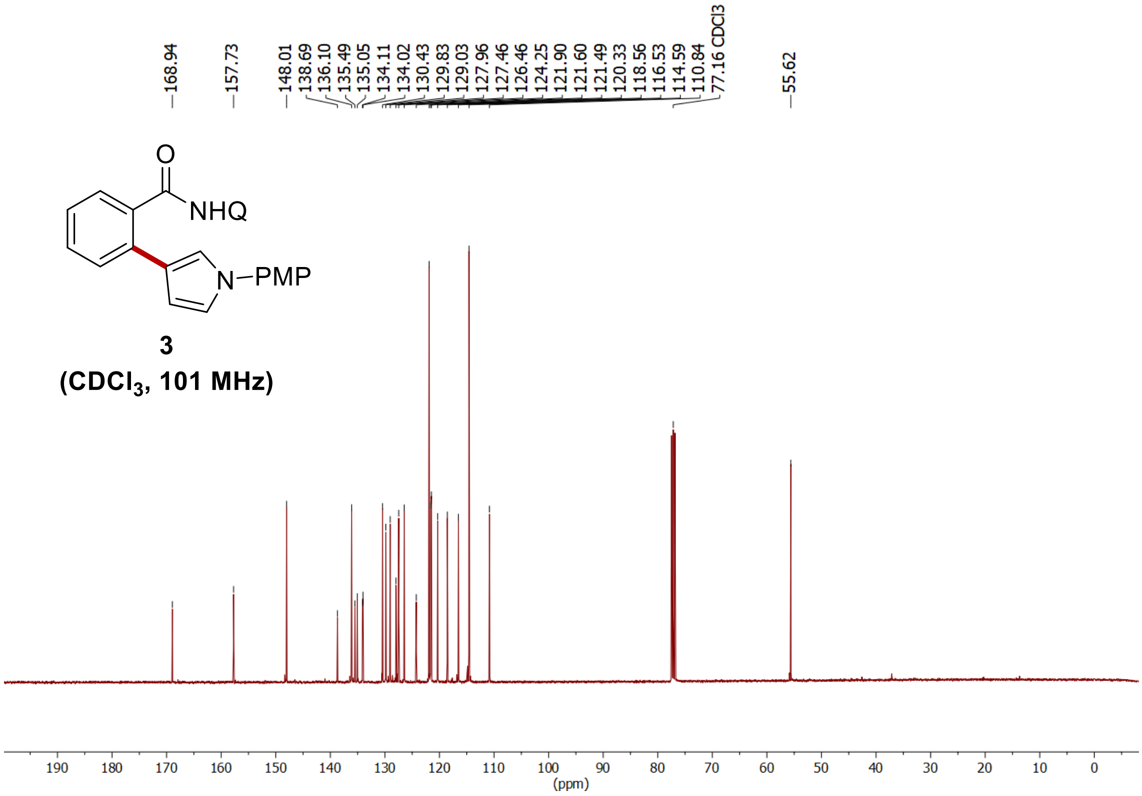

168.94
157.73
148.01
138.69
136.10
135.49
135.05
134.11
134.02
130.43
129.83
129.03
127.96
127.46
126.46
124.25
121.90
121.60
121.49
120.33
118.56
116.53
114.59
110.84
77.16 CDCl3
55.62
O
NHQ
N–PMP
3
(CDCl$_3$, 101 MHz)
(ppm)

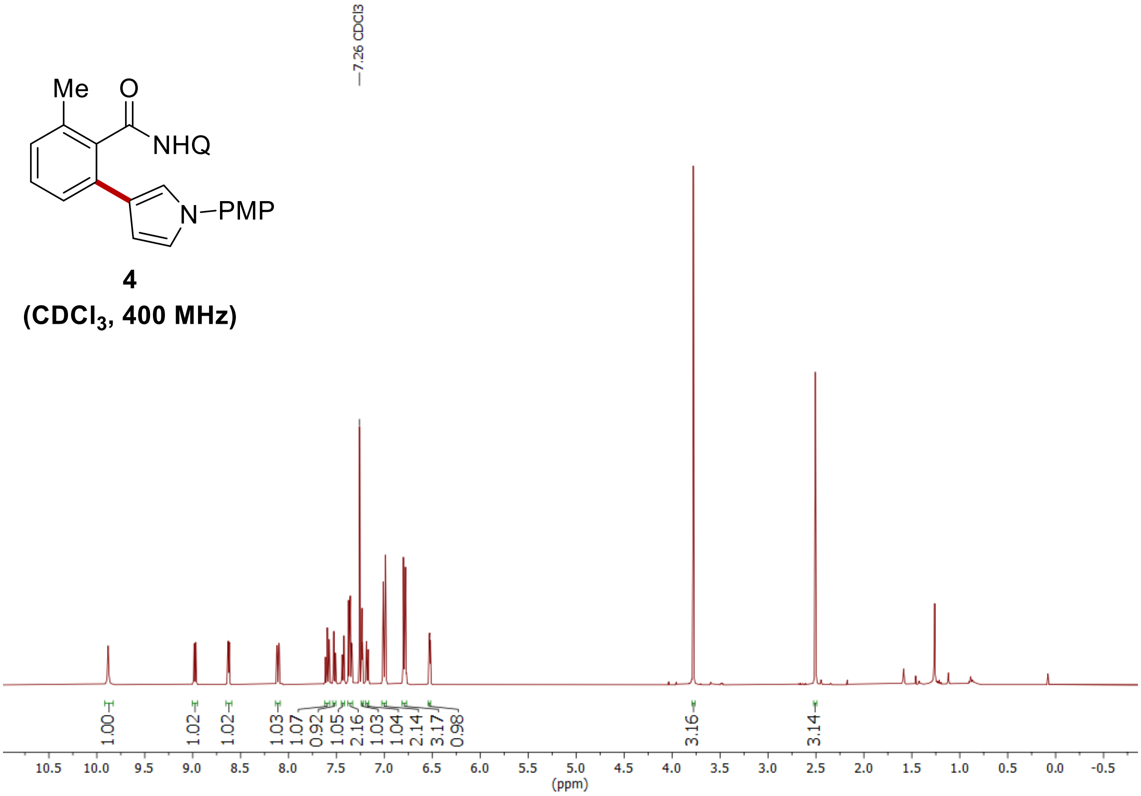
7.26 CDCl3
Me O
NHQ
N–PMP
4
(CDCl3, 400 MHz)
1.00 1.02 1.02 1.03 1.07 0.92 1.05 2.16 1.03 1.04 2.14 3.17 0.98 3.16 3.14
10.5 10.0 9.5 9.0 8.5 8.0 7.5 7.0 6.5 6.0 5.5 5.0 4.5 4.0 3.5 3.0 2.5 2.0 1.5 1.0 0.5 0.0 -0.5
(ppm)

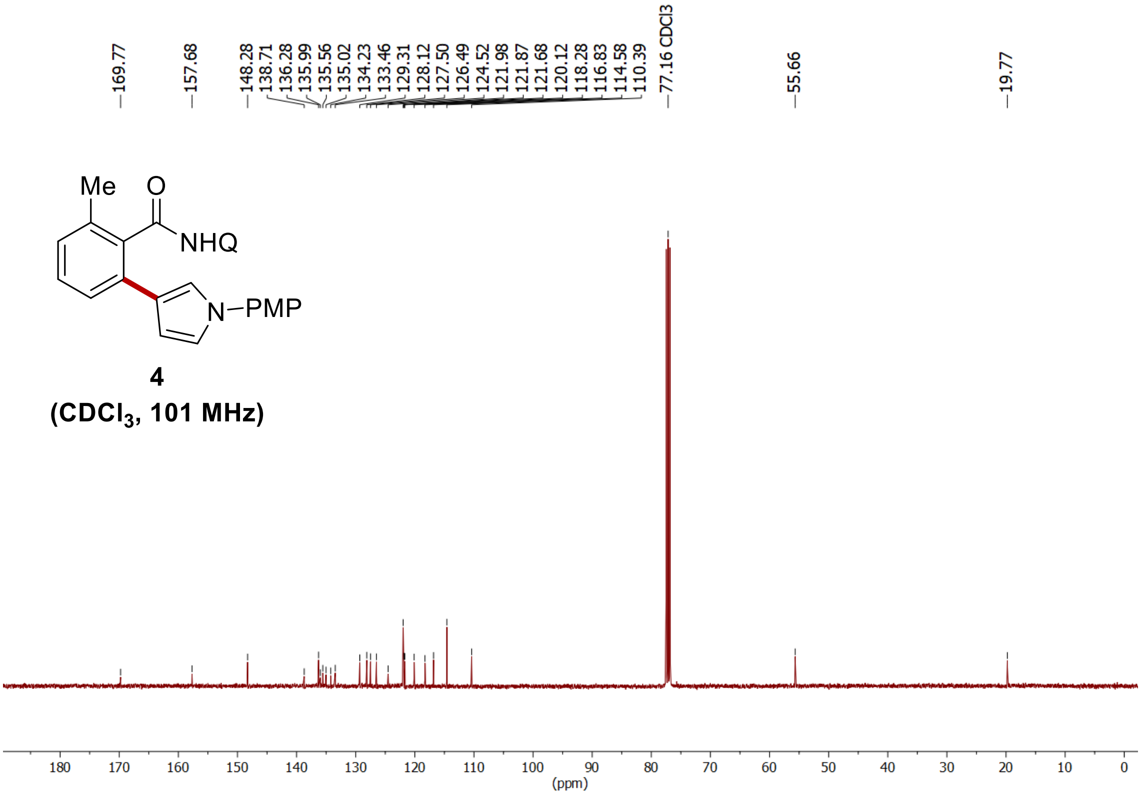
169.77
157.68
148.28 138.71 136.28 135.99 135.56 135.02 134.23 133.46 129.31 128.12 127.50 126.49 124.52 121.98 121.87 121.68 120.12 118.28 116.83 114.58 110.39
77.16 CDCl3
55.66
19.77
Me O
NHQ
N–PMP
4
(CDCl3, 101 MHz)
180 170 160 150 140 130 120 110 100 90 80 70 60 50 40 30 20 10 0
(ppm)

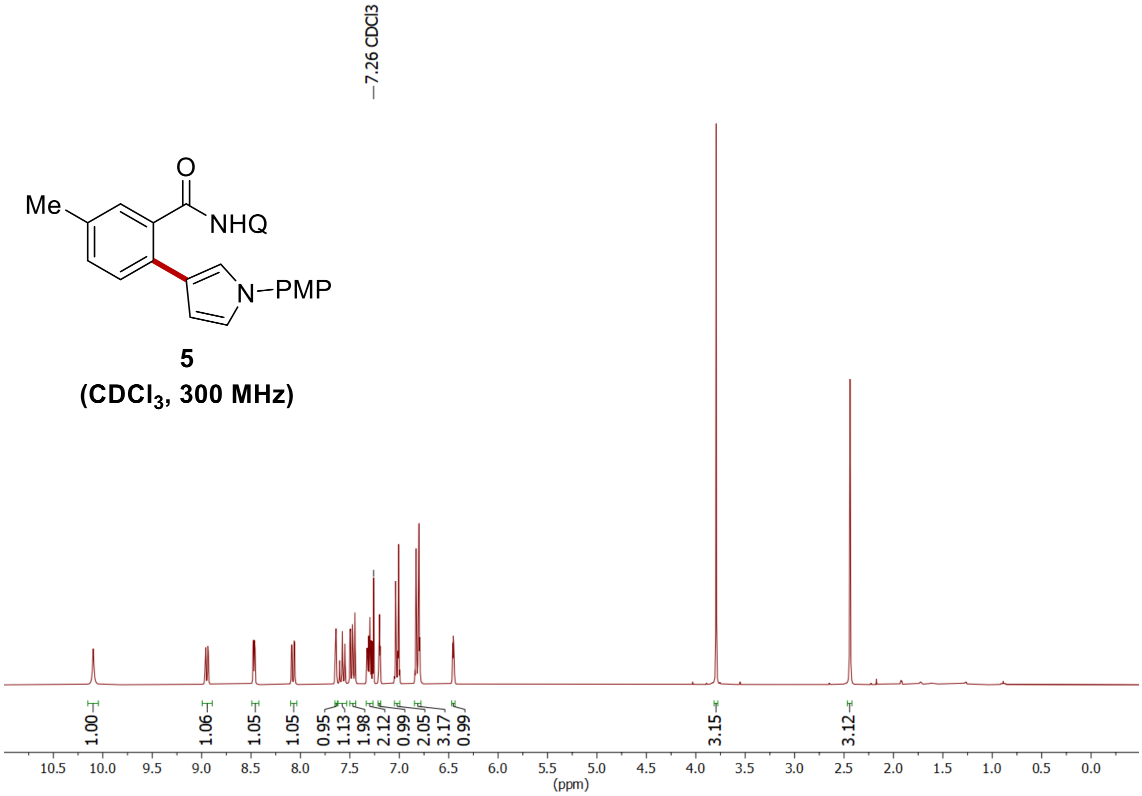
7.26 CDCl3
Me
O
NHQ
N–PMP
5
(CDCl$_3$, 300 MHz)
1.00
1.06
1.05
1.05
0.95
1.13
1.98
2.12
0.99
2.05
3.17
0.99
3.15
3.12
10.5 10.0 9.5 9.0 8.5 8.0 7.5 7.0 6.5 6.0 5.5 5.0 4.5 4.0 3.5 3.0 2.5 2.0 1.5 1.0 0.5 0.0
(ppm)

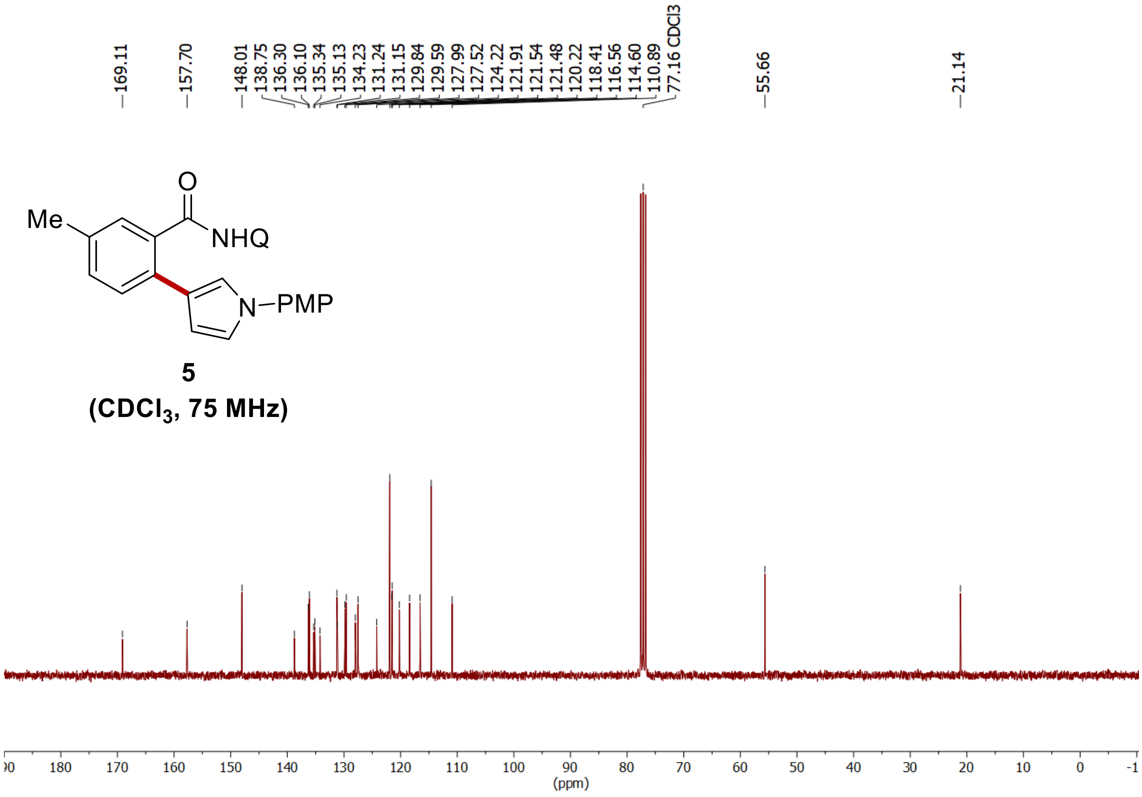
169.11
157.70
148.01
138.75
136.30
136.10
135.34
135.13
134.23
131.24
131.15
129.84
129.59
127.99
127.52
124.22
121.91
121.54
121.48
120.22
118.41
116.56
114.60
110.89
77.16 CDCl3
55.66
21.14
Me
O
NHQ
N–PMP
5
(CDCl$_3$, 75 MHz)
180 170 160 150 140 130 120 110 100 90 80 70 60 50 40 30 20 10 0 -1
(ppm)

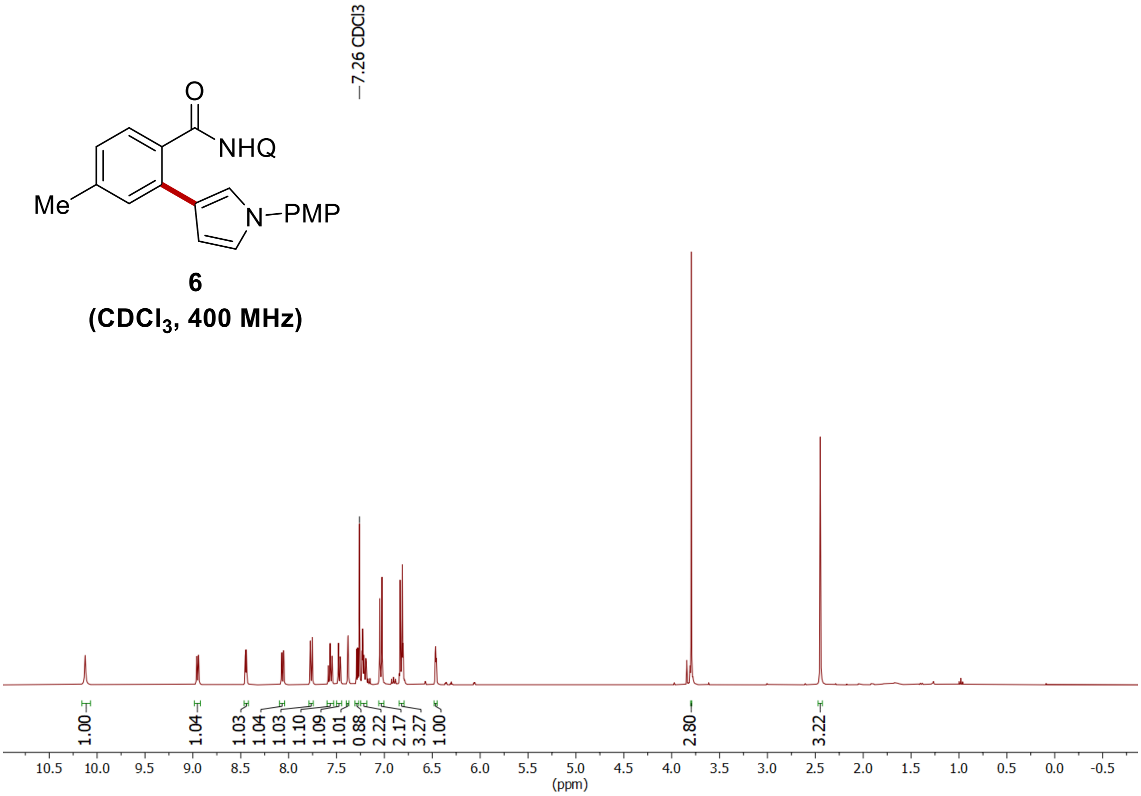
O
NHQ
Me
N–PMP
6
(CDCl3, 400 MHz)
7.26 CDCl3
1.00
1.04
1.03
1.04
1.03
1.10
1.09
1.01
0.88
2.22
2.17
3.27
1.00
2.80
3.22
(ppm)

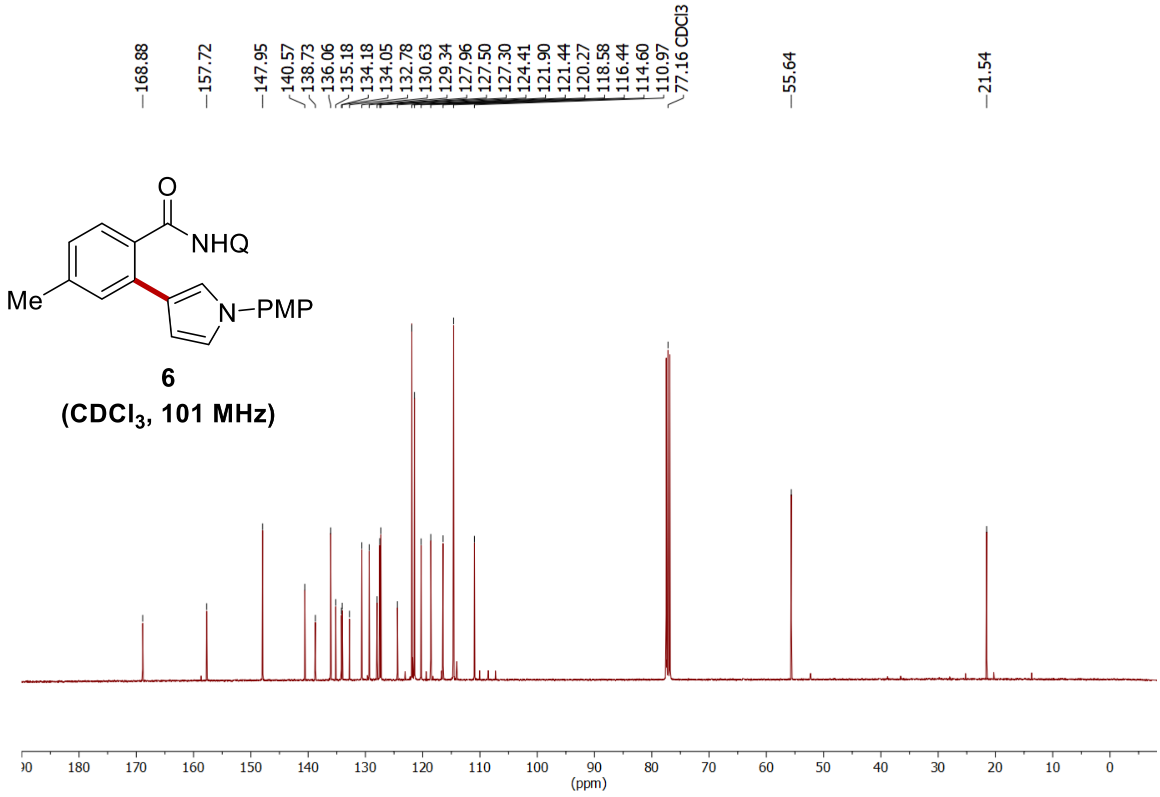
168.88
157.72
147.95
140.57
138.73
136.06
135.18
134.18
134.05
132.78
130.63
129.34
127.96
127.50
127.30
124.41
121.90
121.44
120.27
118.58
116.44
114.60
110.97
77.16 CDCl3
55.64
21.54
O
NHQ
Me
N–PMP
6
(CDCl3, 101 MHz)
(ppm)

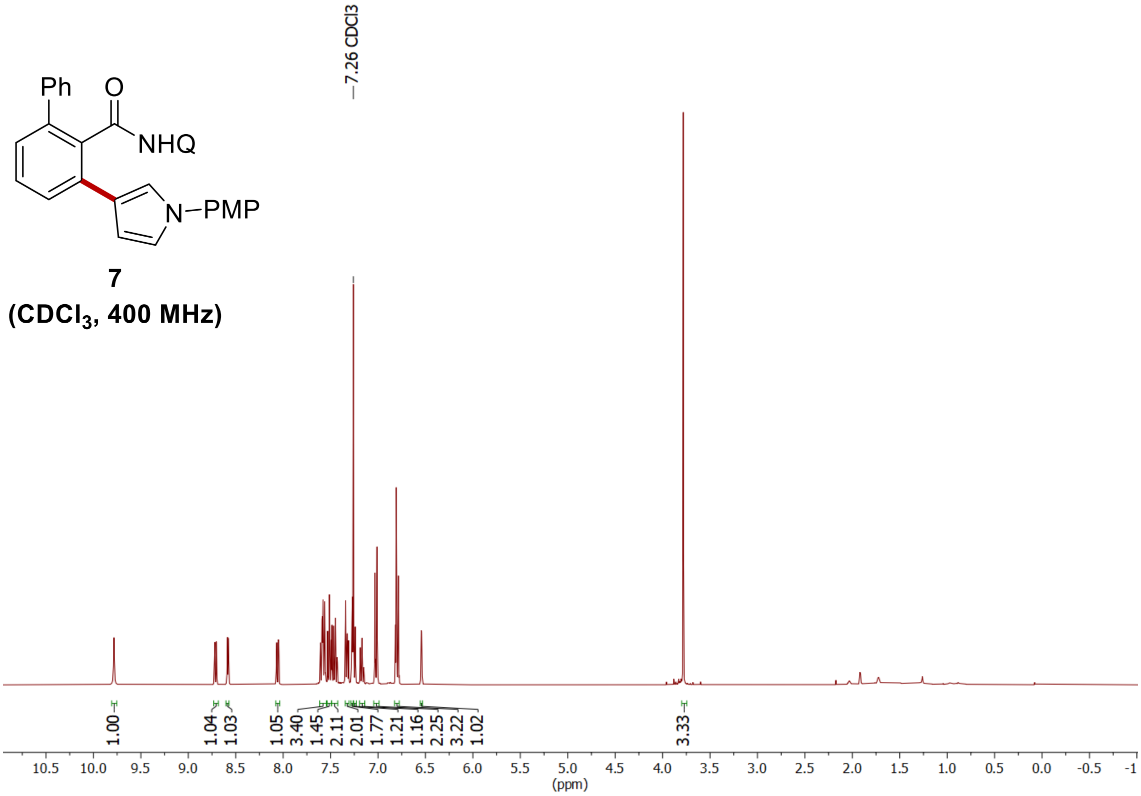
7.26 CDCl3
Ph
O
NHQ
N–PMP
7
(CDCl3, 400 MHz)
1.00
1.04
1.03
1.05
3.40
1.45
2.11
2.01
1.77
1.21
1.16
2.25
3.22
1.02
3.33
(ppm)

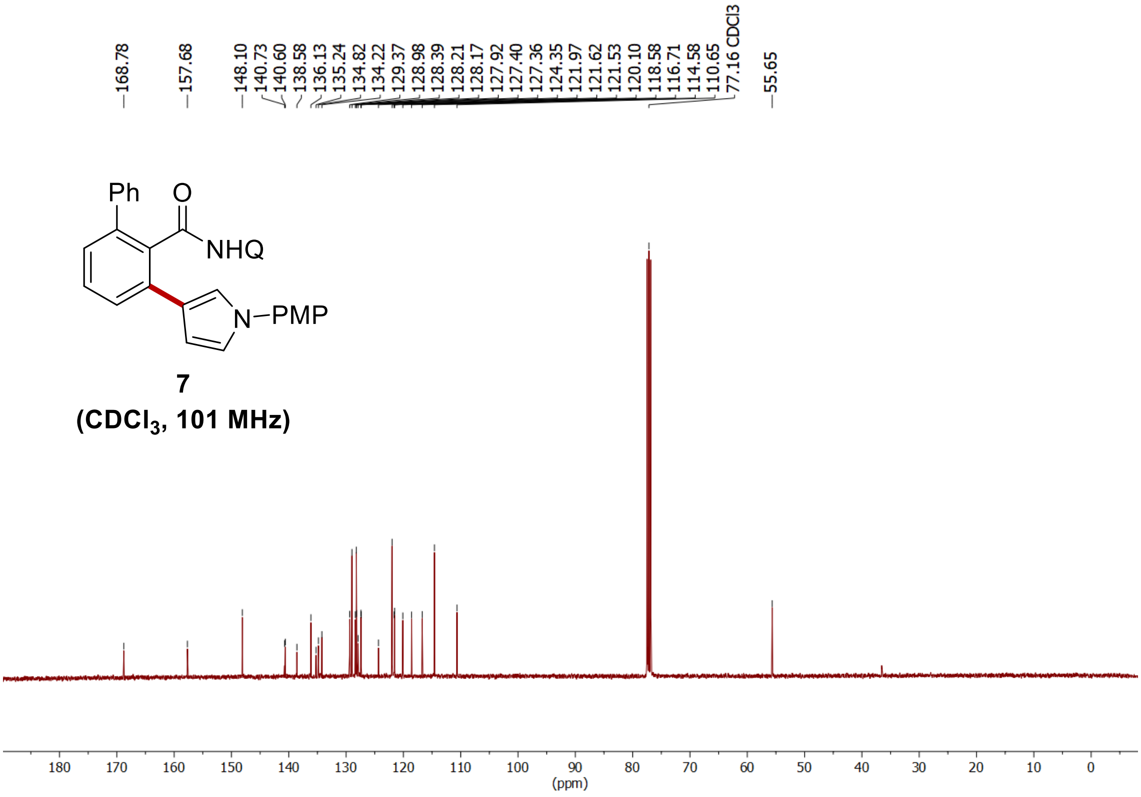
168.78
157.68
148.10
140.73
140.60
138.58
136.13
135.24
134.82
134.22
129.37
128.98
128.39
128.21
128.17
127.92
127.40
127.36
124.35
121.97
121.62
121.53
120.10
118.58
116.71
114.58
110.65
77.16 CDCl3
55.65
Ph
O
NHQ
N–PMP
7
(CDCl3, 101 MHz)
(ppm)

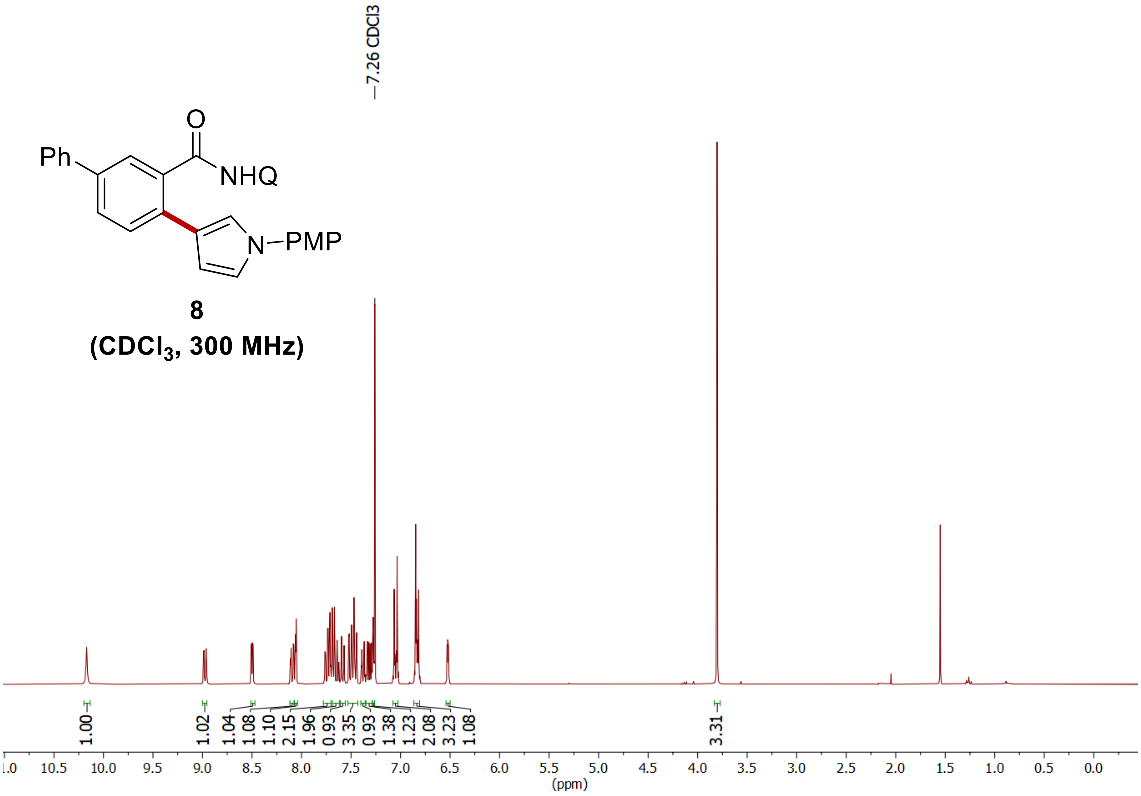
Ph
O
NHQ
N–PMP
8
(CDCl3, 300 MHz)
7.26 CDCl3
1.00
1.02
1.04
1.08
1.10
2.15
1.96
0.93
3.35
0.93
1.38
1.23
2.08
3.23
1.08
3.31
(ppm)

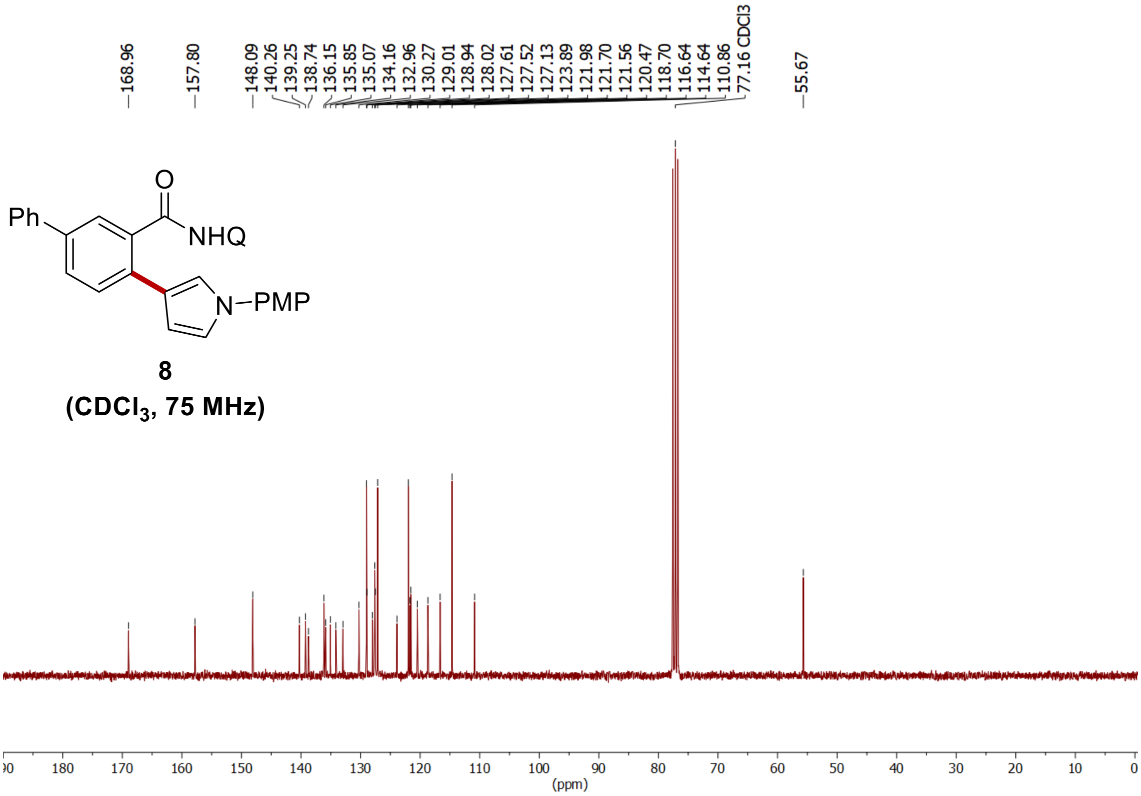
Ph
O
NHQ
N–PMP
8
(CDCl3, 75 MHz)
168.96
157.80
148.09
140.26
139.25
138.74
136.15
135.85
135.07
134.16
132.96
130.27
129.01
128.94
128.02
127.61
127.52
127.13
123.89
121.98
121.70
121.56
120.47
118.70
116.64
114.64
110.86
77.16 CDCl3
55.67
(ppm)

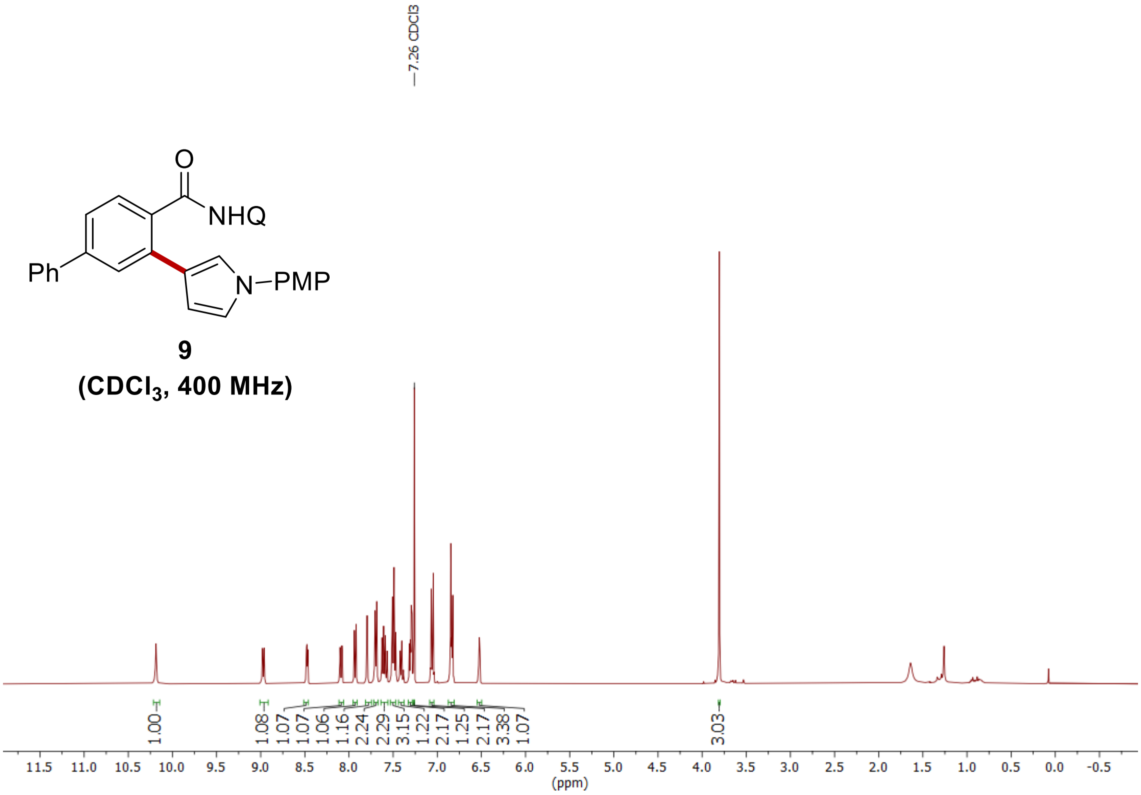
7.26 CDCl3
O
NHQ
Ph
N−PMP
9
(CDCl3, 400 MHz)
1.00
1.08
1.07
1.07
1.06
1.16
2.24
2.29
3.15
1.22
2.17
1.25
2.17
3.38
1.07
3.03
11.5 11.0 10.5 10.0 9.5 9.0 8.5 8.0 7.5 7.0 6.5 6.0 5.5 5.0 4.5 4.0 3.5 3.0 2.5 2.0 1.5 1.0 0.5 0.0 -0.5
(ppm)

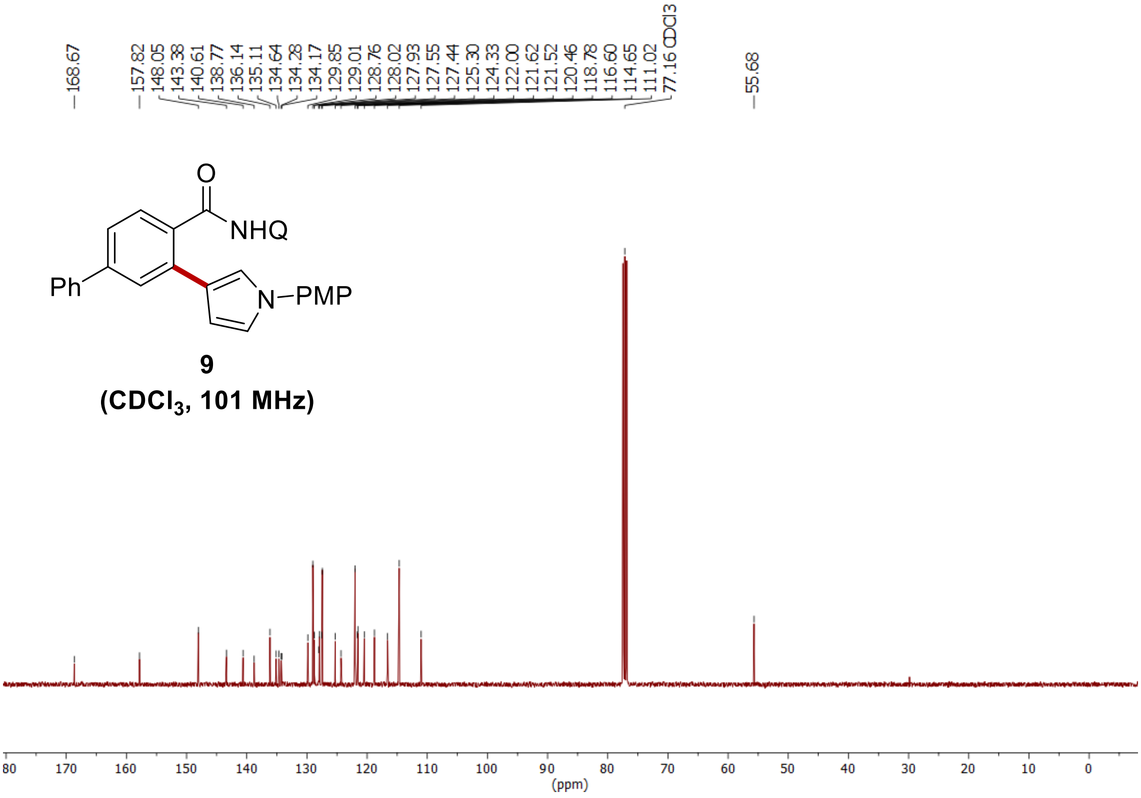
168.67
157.82
148.05
143.38
140.61
138.77
136.14
135.11
134.64
134.28
134.17
129.85
129.01
128.76
128.02
127.93
127.55
127.44
125.30
124.33
122.00
121.62
121.52
120.46
118.78
116.60
114.65
111.02
77.16 CDCl3
55.68
O
NHQ
Ph
N−PMP
9
(CDCl3, 101 MHz)
80 170 160 150 140 130 120 110 100 90 80 70 60 50 40 30 20 10 0
(ppm)

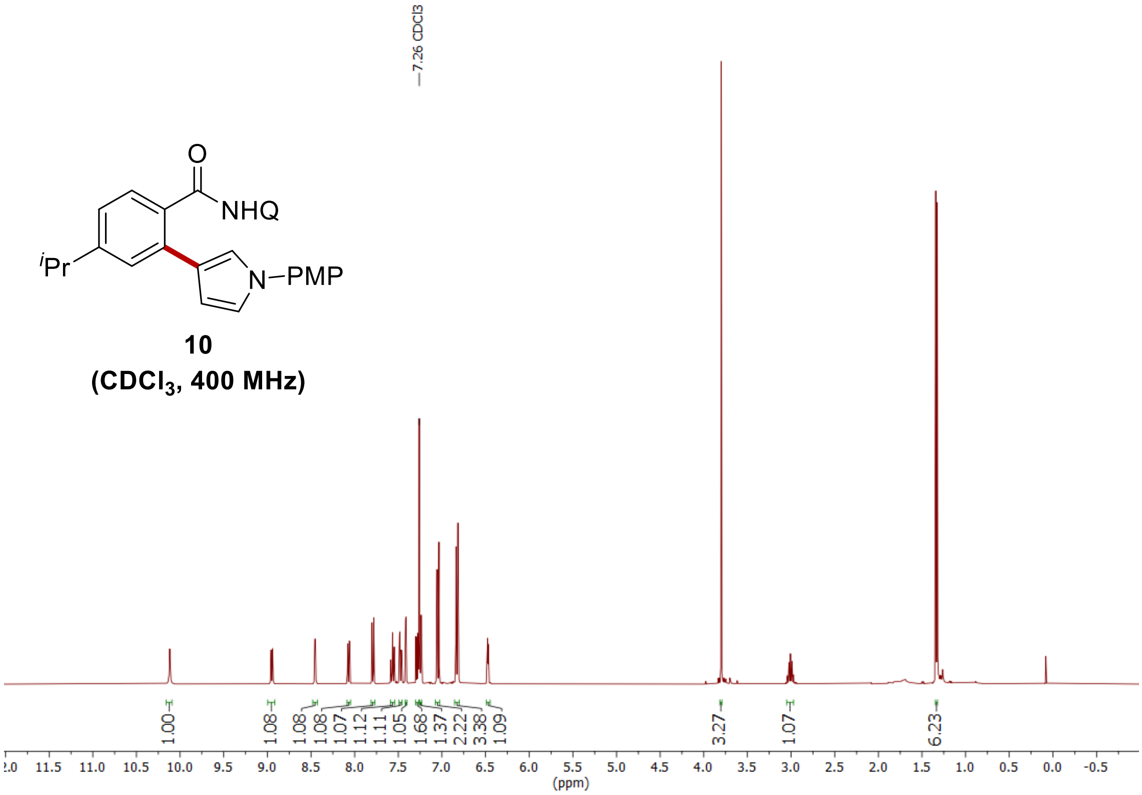
7.26 CDCl3
O
NHQ
iPr
N–PMP
10
(CDCl3, 400 MHz)
1.00
1.08
1.08
1.08
1.07
1.12
1.11
1.05
1.68
1.37
2.22
3.38
1.09
3.27
1.07
6.23
(ppm)

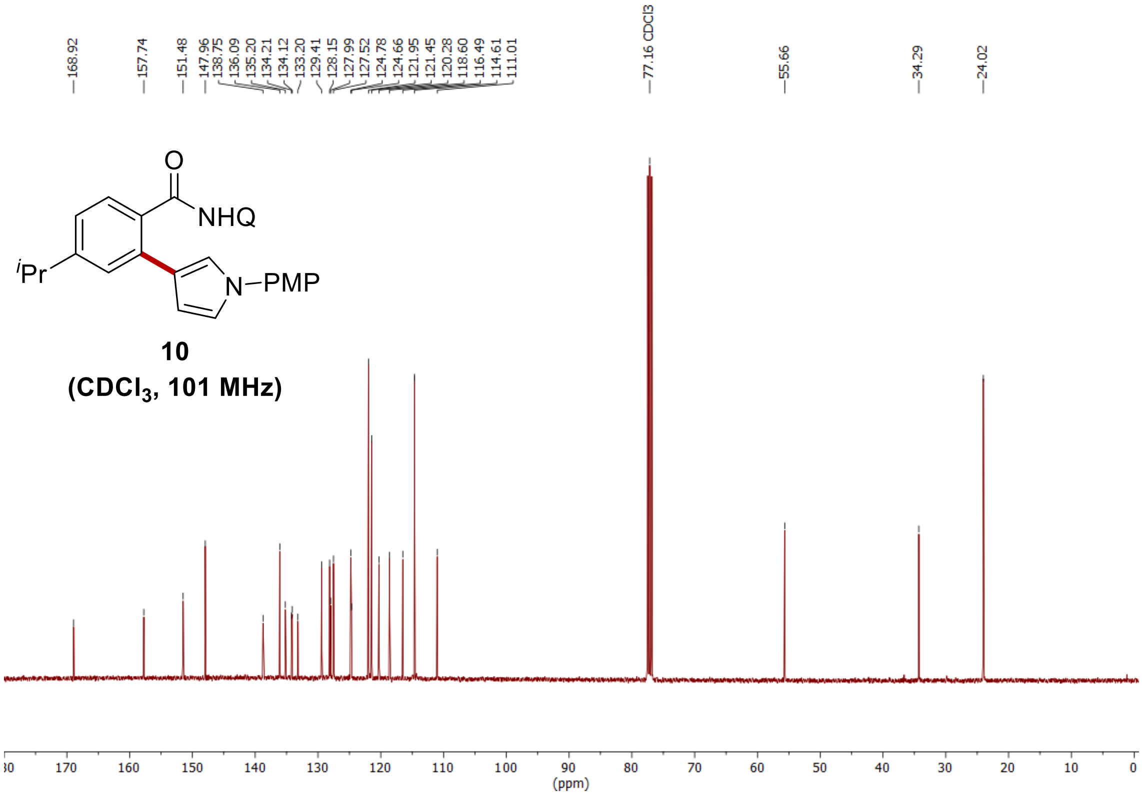
168.92
157.74
151.48
147.96
138.75
136.09
135.20
134.21
134.12
133.20
129.41
128.15
127.99
127.52
124.78
124.66
121.95
121.45
120.28
118.60
116.49
114.61
111.01
77.16 CDCl3
55.66
34.29
24.02
O
NHQ
iPr
N–PMP
10
(CDCl3, 101 MHz)
(ppm)

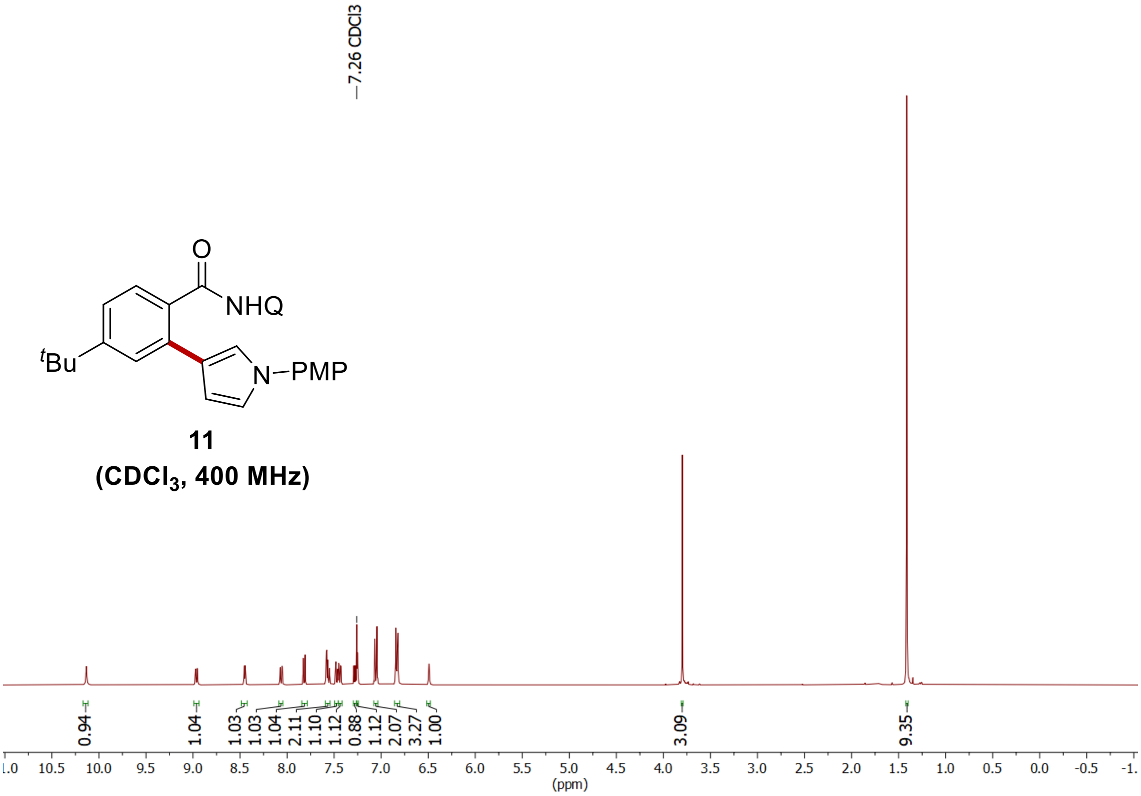
7.26 CDCl3
O
NHQ
tBu
N−PMP
11
(CDCl3, 400 MHz)
0.94
1.04
1.03
1.03
1.04
2.11
1.10
1.12
0.88
1.12
2.07
3.27
1.00
3.09
9.35
(ppm)

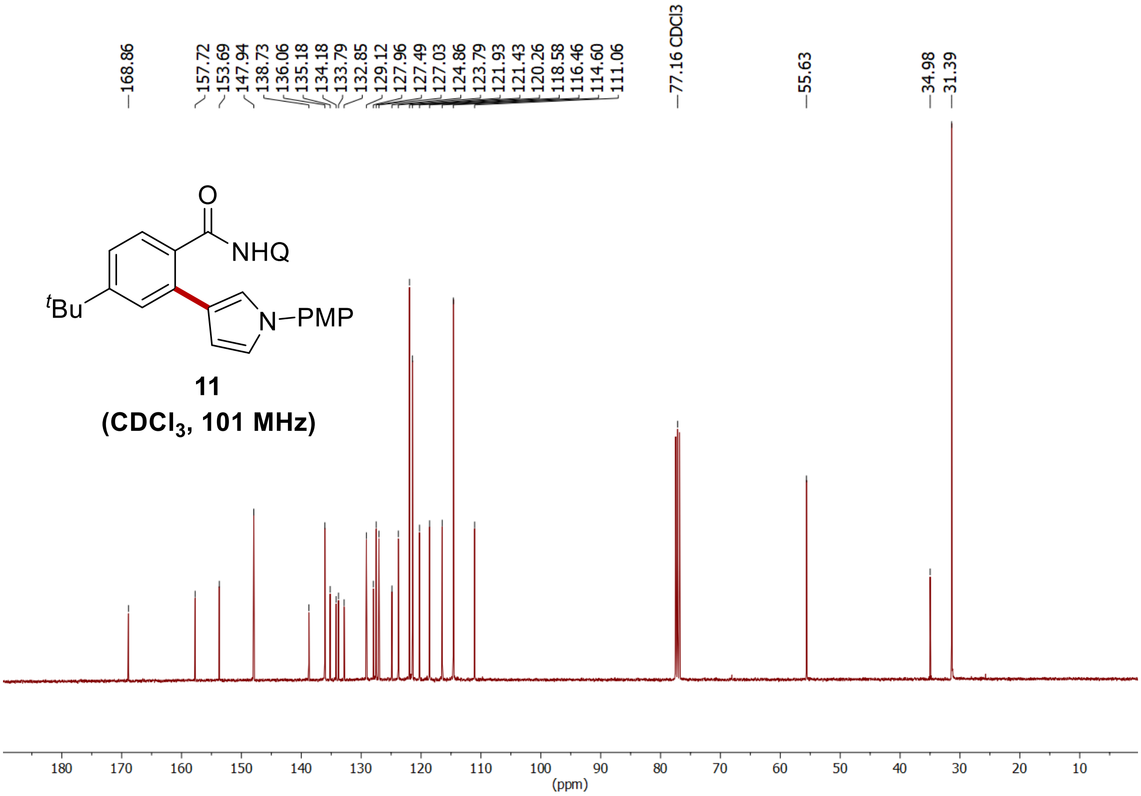
168.86
157.72
153.69
147.94
138.73
136.06
135.18
134.18
133.79
132.85
129.12
127.96
127.49
127.03
124.86
123.79
121.93
121.43
120.26
118.58
116.46
114.60
111.06
77.16 CDCl3
55.63
34.98
31.39
O
NHQ
tBu
N−PMP
11
(CDCl3, 101 MHz)
(ppm)

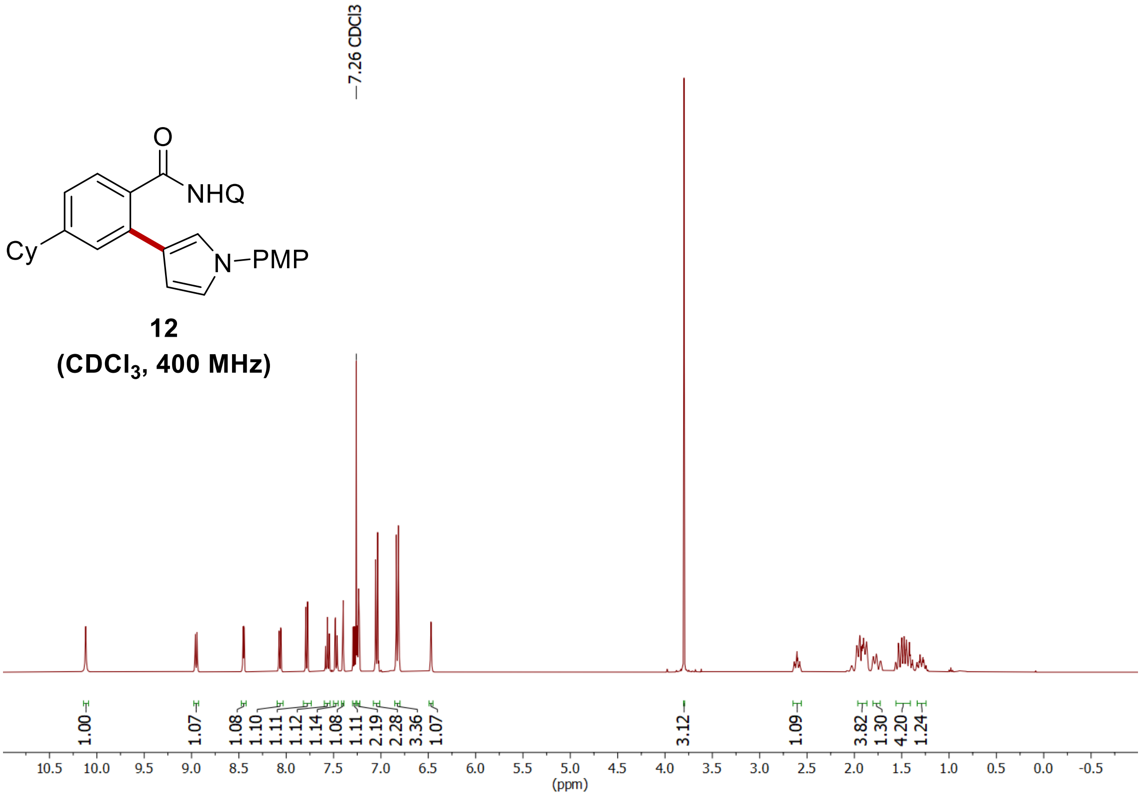

7.26 CDCl3
O
NHQ
Cy
N–PMP
12
(CDCl3, 400 MHz)
1.00
1.07
1.08
1.10
1.11
1.12
1.14
1.08
1.11
2.19
2.28
3.36
1.07
3.12
1.09
3.82
1.30
4.20
1.24
10.5 10.0 9.5 9.0 8.5 8.0 7.5 7.0 6.5 6.0 5.5 5.0 4.5 4.0 3.5 3.0 2.5 2.0 1.5 1.0 0.5 0.0 -0.5
(ppm)

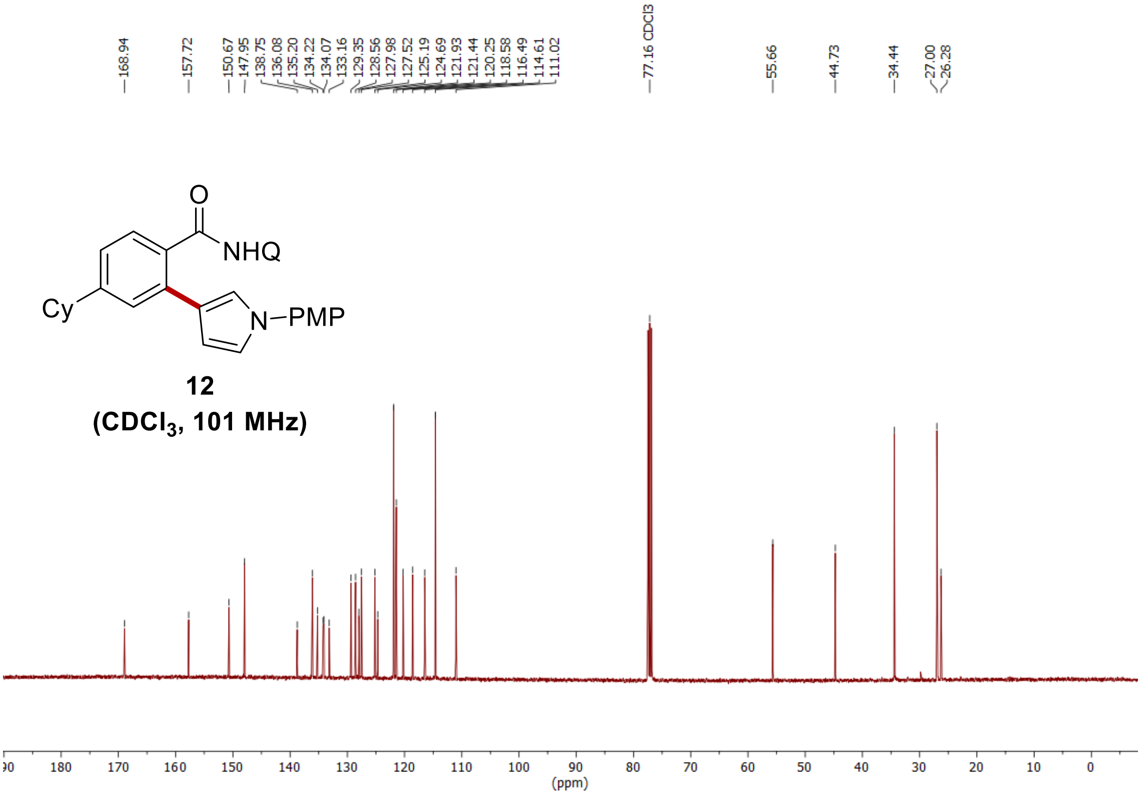

168.94
157.72
150.67
147.95
138.75
136.08
135.20
134.22
134.07
133.16
129.35
128.56
127.98
127.52
125.19
124.69
121.93
121.44
120.25
118.58
116.49
114.61
111.02
77.16 CDCl3
55.66
44.73
34.44
27.00
26.28
O
NHQ
Cy
N–PMP
12
(CDCl3, 101 MHz)
180 170 160 150 140 130 120 110 100 90 80 70 60 50 40 30 20 10 0
(ppm)

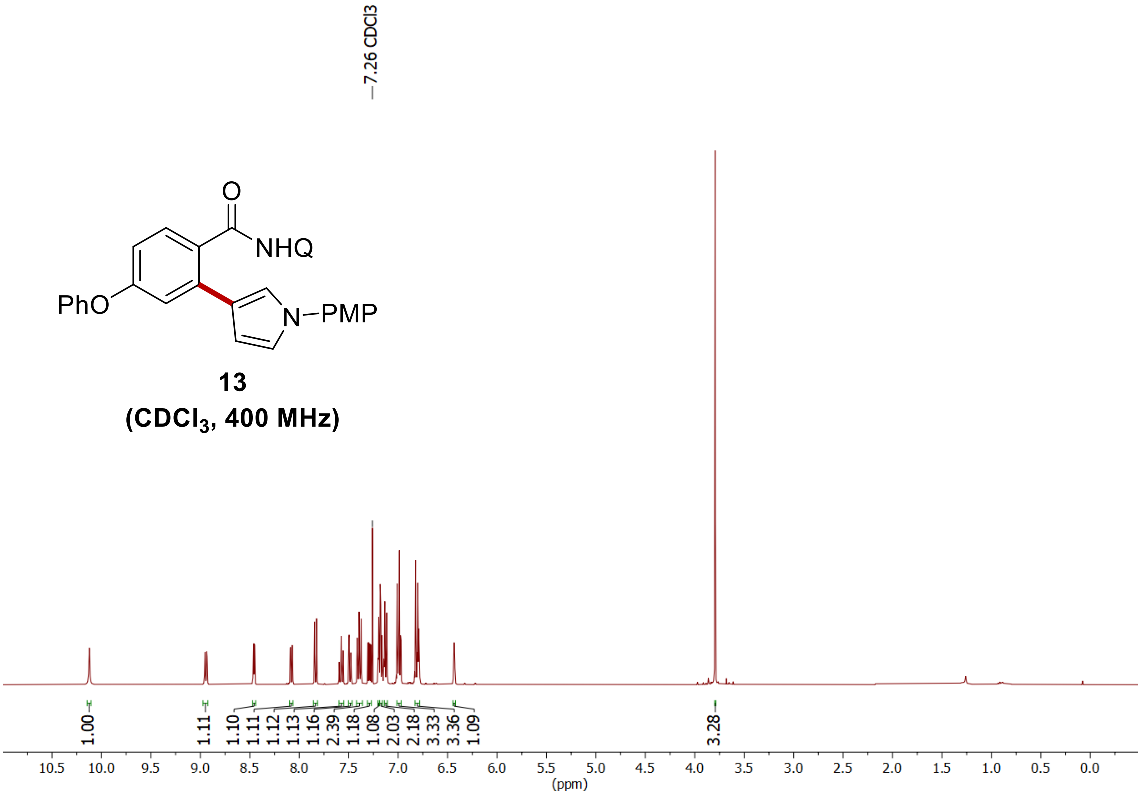

13
(CDCl$_3$, 400 MHz)

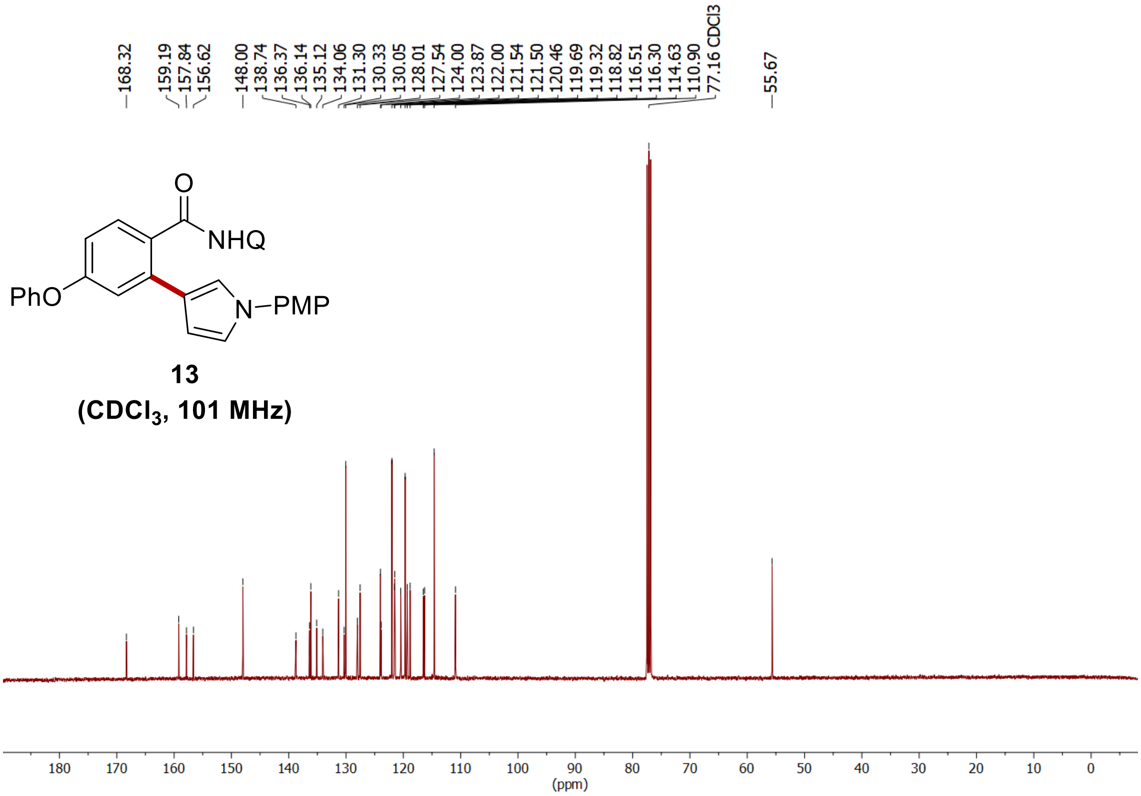

13
(CDCl$_3$, 101 MHz)

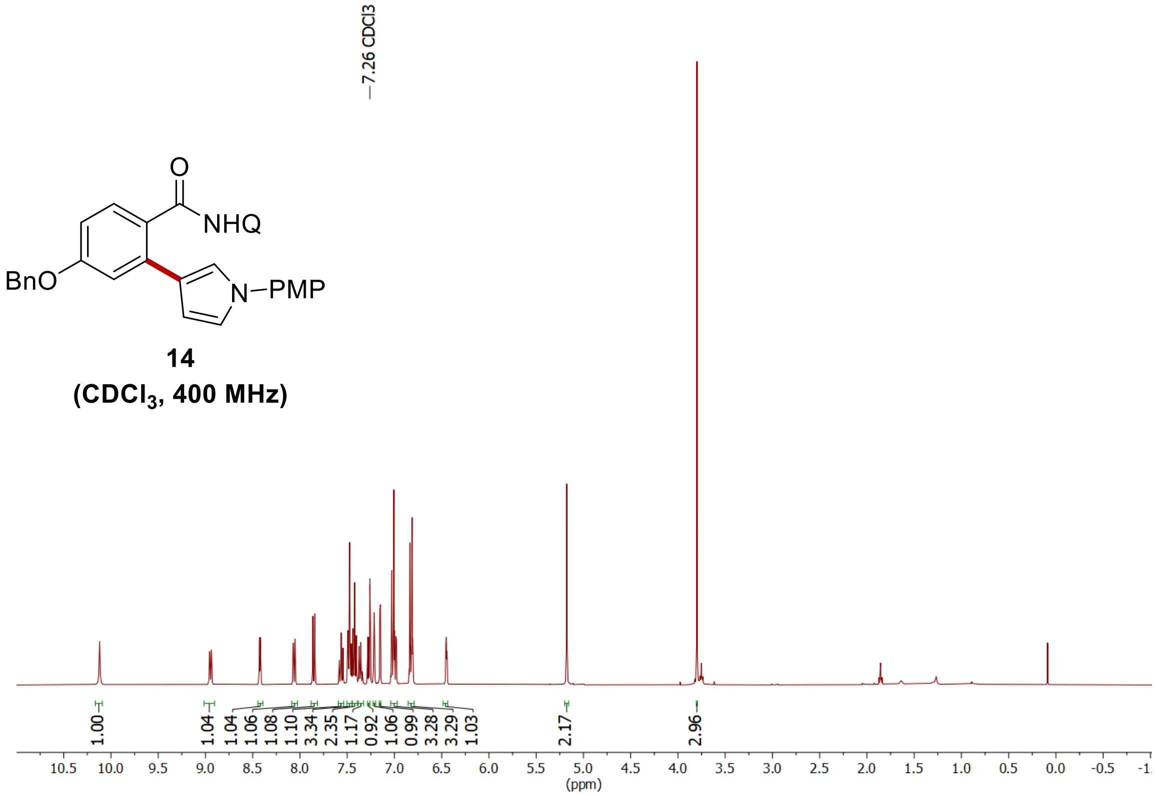
7.26 CDCl3
O
NHQ
BnO
N–PMP
14
(CDCl$_3$, 400 MHz)
1.00
1.04
1.04
1.06
1.08
1.10
3.34
2.35
1.17
0.92
1.06
0.99
3.28
3.29
1.03
2.17
2.96
10.5 10.0 9.5 9.0 8.5 8.0 7.5 7.0 6.5 6.0 5.5 5.0 4.5 4.0 3.5 3.0 2.5 2.0 1.5 1.0 0.5 0.0 -0.5 -1
(ppm)

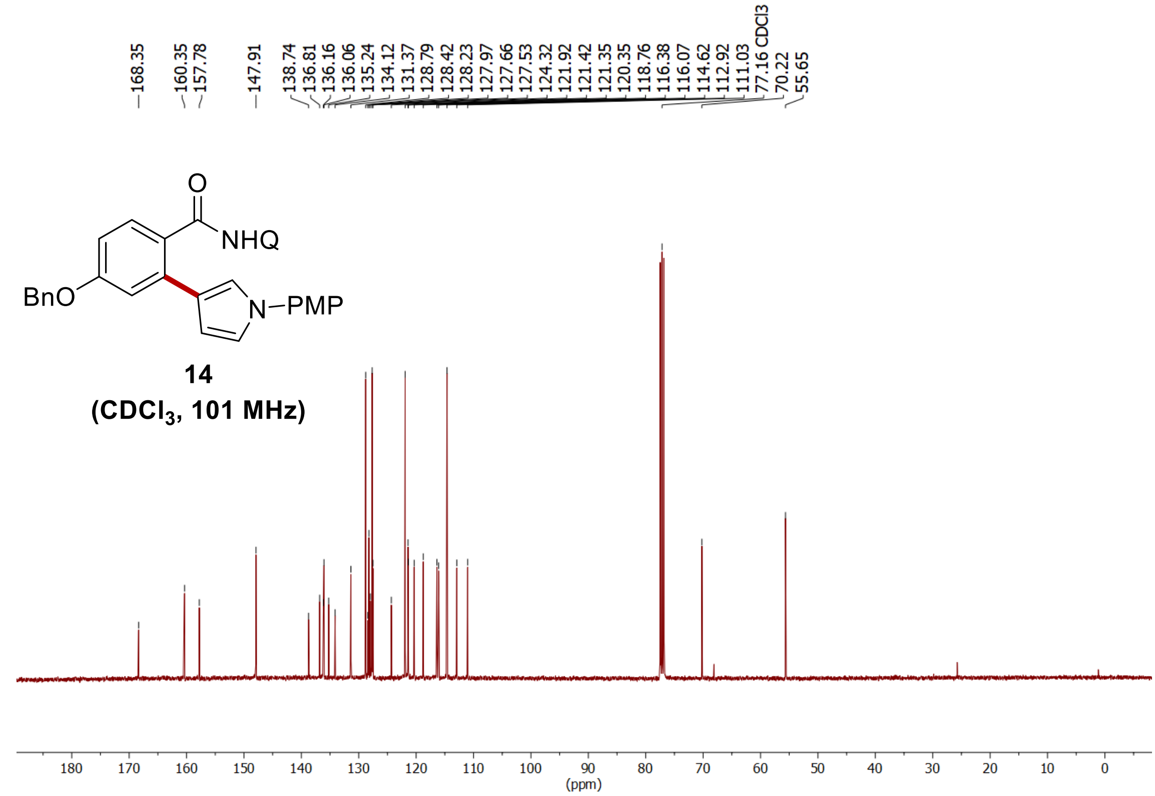
168.35
160.35
157.78
147.91
138.74
136.81
136.16
136.06
135.24
134.12
131.37
128.79
128.42
128.23
127.97
127.66
127.53
124.32
121.92
121.42
121.35
120.35
118.76
116.38
116.07
114.62
112.92
111.03
77.16 CDCl3
70.22
55.65
O
NHQ
BnO
N–PMP
14
(CDCl$_3$, 101 MHz)
180 170 160 150 140 130 120 110 100 90 80 70 60 50 40 30 20 10 0
(ppm)

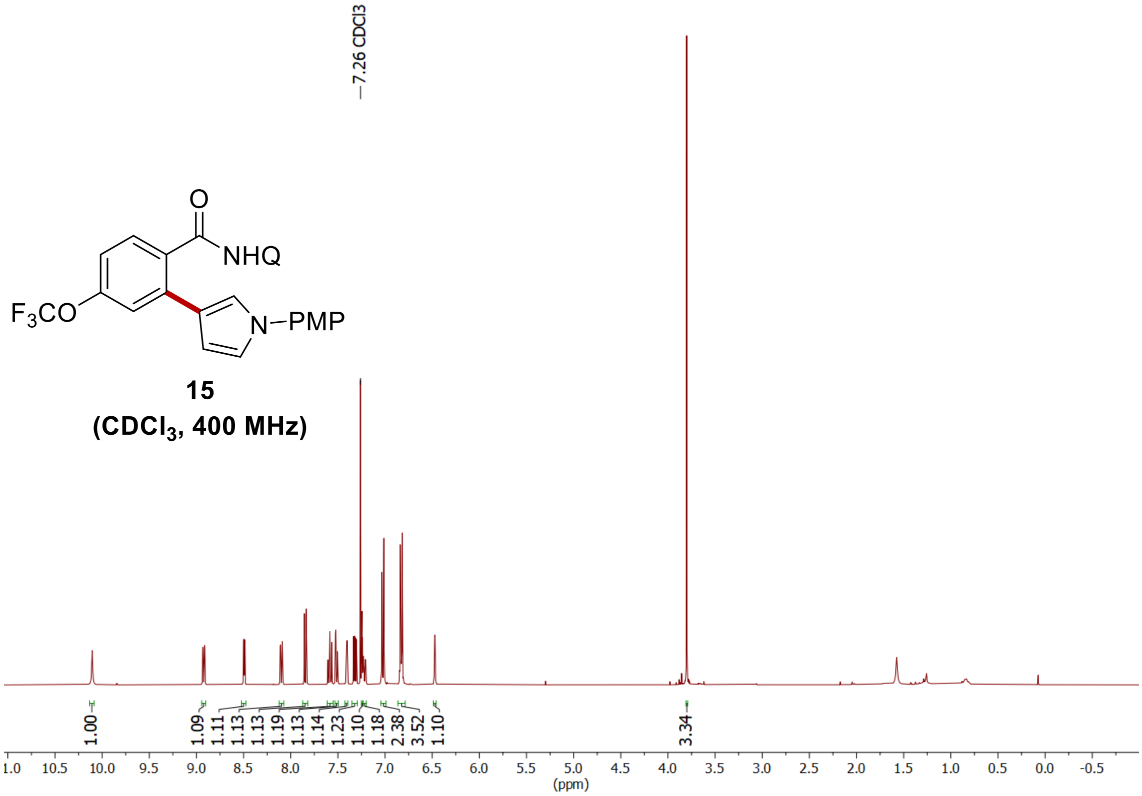
7.26 CDCl3
O
NHQ
F3CO
N–PMP
15
(CDCl3, 400 MHz)
1.00
1.09
1.11
1.13
1.13
1.19
1.13
1.14
1.23
1.10
1.18
2.38
3.52
1.10
3.34
(ppm)

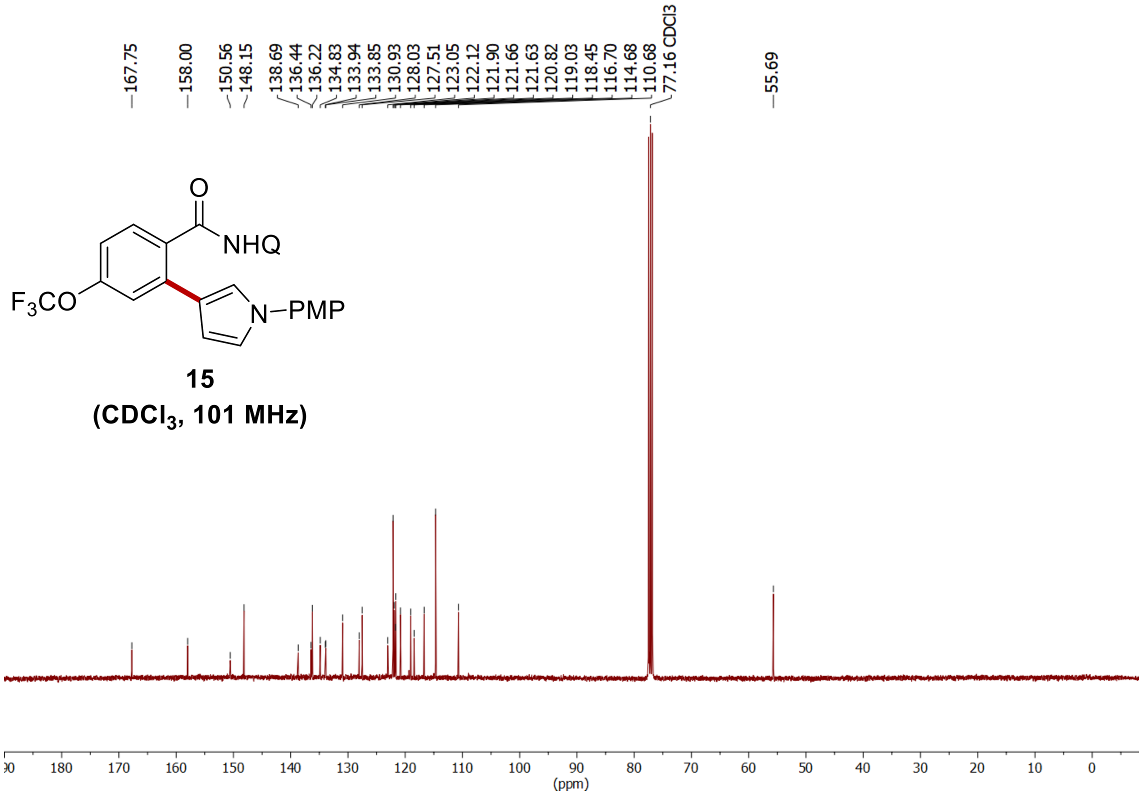
167.75
158.00
150.56
148.15
138.69
136.44
136.22
134.83
133.94
133.85
130.93
128.03
127.51
123.05
122.12
121.90
121.66
121.63
120.82
119.03
118.45
116.70
114.68
110.68
77.16 CDCl3
55.69
O
NHQ
F3CO
N–PMP
15
(CDCl3, 101 MHz)
(ppm)

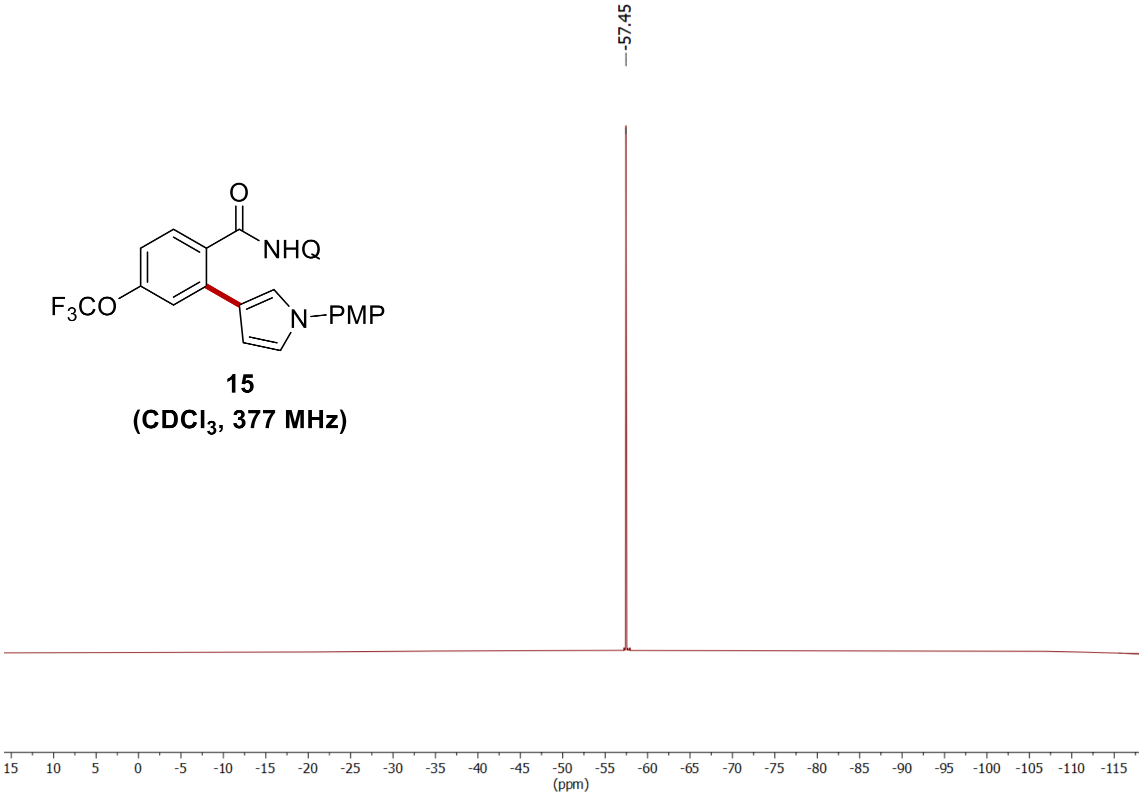
-57.45
O
NHQ
F3CO
N–PMP
15
(CDCl3, 377 MHz)
15 10 5 0 -5 -10 -15 -20 -25 -30 -35 -40 -45 -50 -55 -60 -65 -70 -75 -80 -85 -90 -95 -100 -105 -110 -115
(ppm)

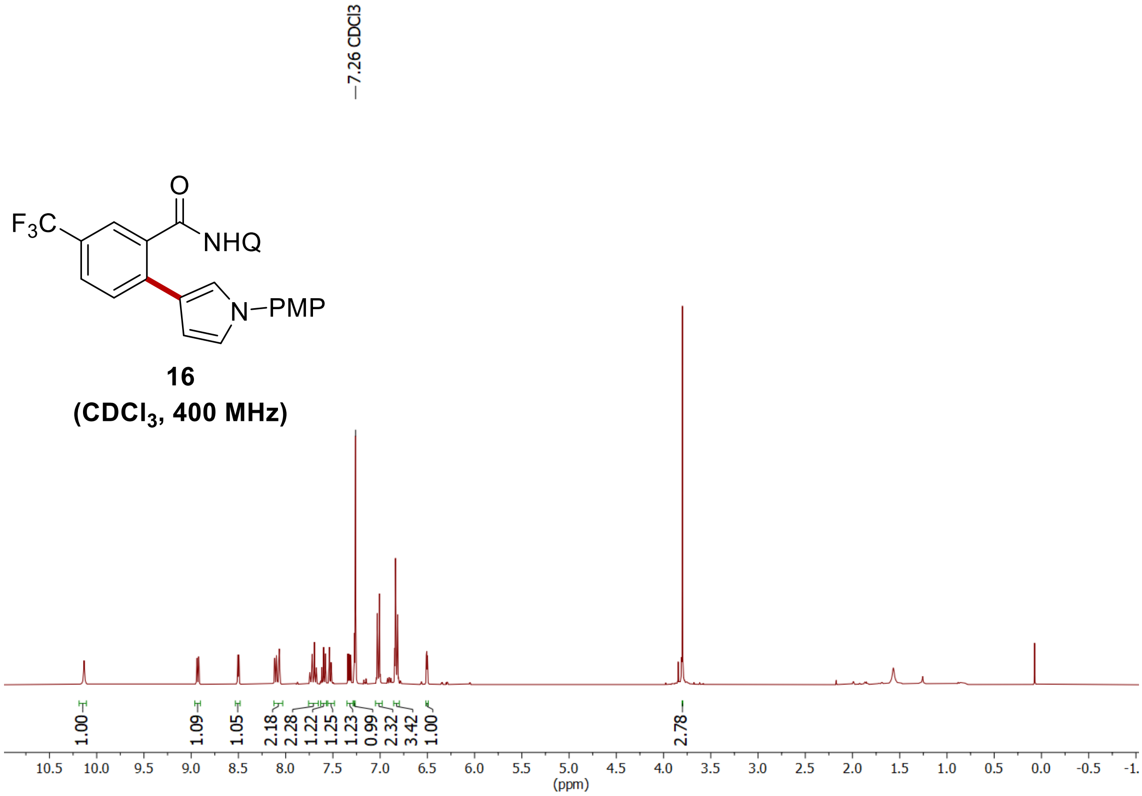
7.26 CDCl3
O
F3C
NHQ
N–PMP
16
(CDCl3, 400 MHz)
1.00
1.09
1.05
2.18
2.28
1.22
1.25
1.23
0.99
2.32
3.42
1.00
2.78
10.5 10.0 9.5 9.0 8.5 8.0 7.5 7.0 6.5 6.0 5.5 5.0 4.5 4.0 3.5 3.0 2.5 2.0 1.5 1.0 0.5 0.0 -0.5 -1.
(ppm)

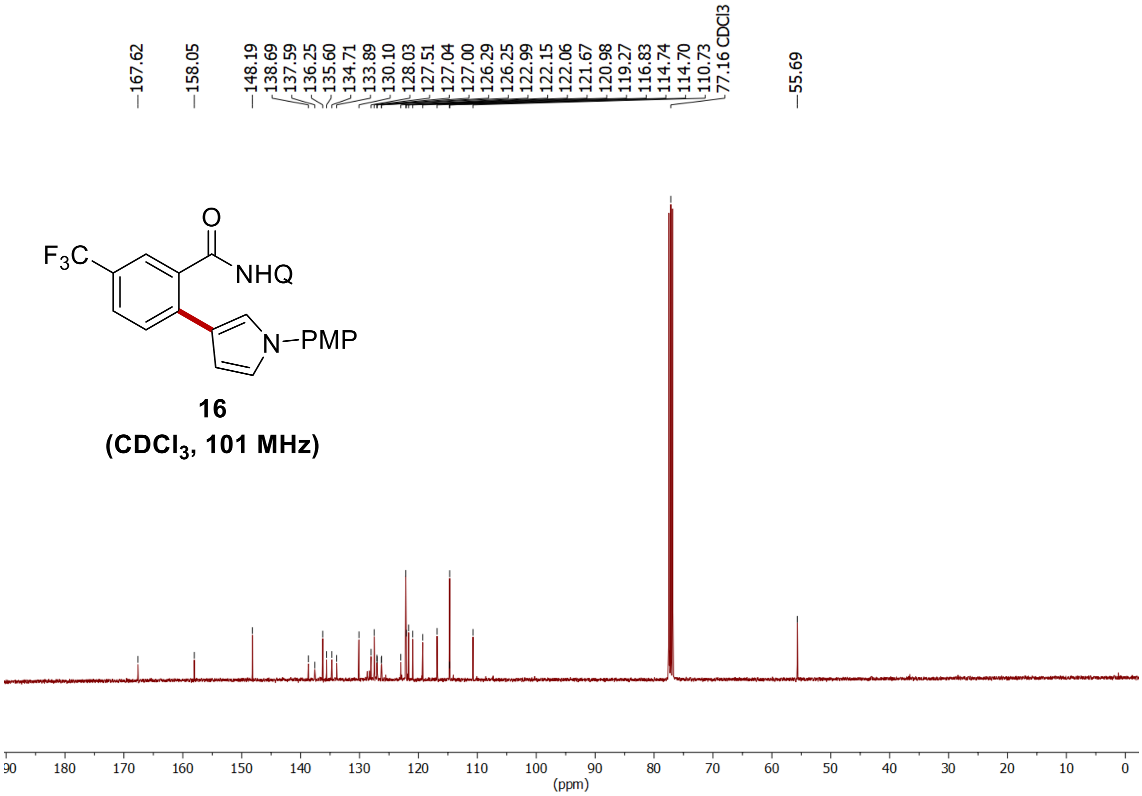
167.62
158.05
148.19
138.69
137.59
136.25
135.60
134.71
133.89
130.10
128.03
127.51
127.04
127.00
126.29
126.25
122.99
122.15
122.06
121.67
120.98
119.27
116.83
114.74
114.70
110.73
77.16 CDCl3
55.69
F3C
O
NHQ
N–PMP
16
(CDCl3, 101 MHz)
(ppm)

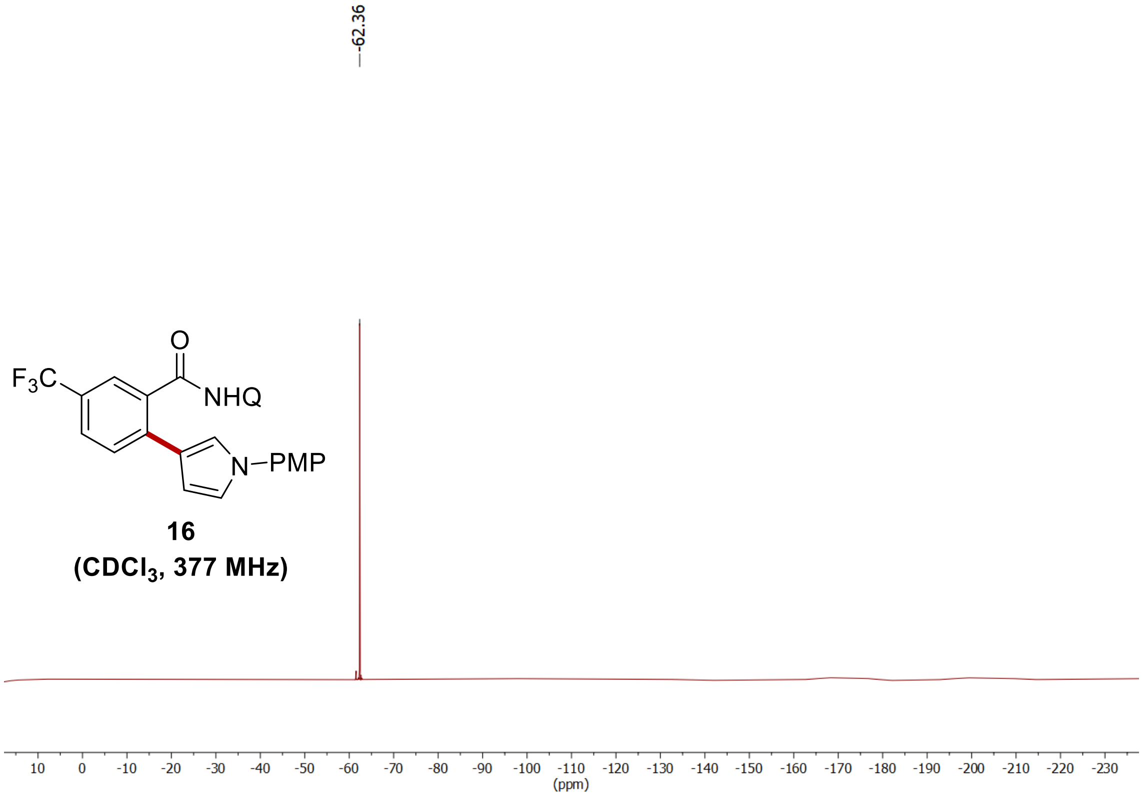
-62.36
F3C
O
NHQ
N–PMP
16
(CDCl3, 377 MHz)
(ppm)

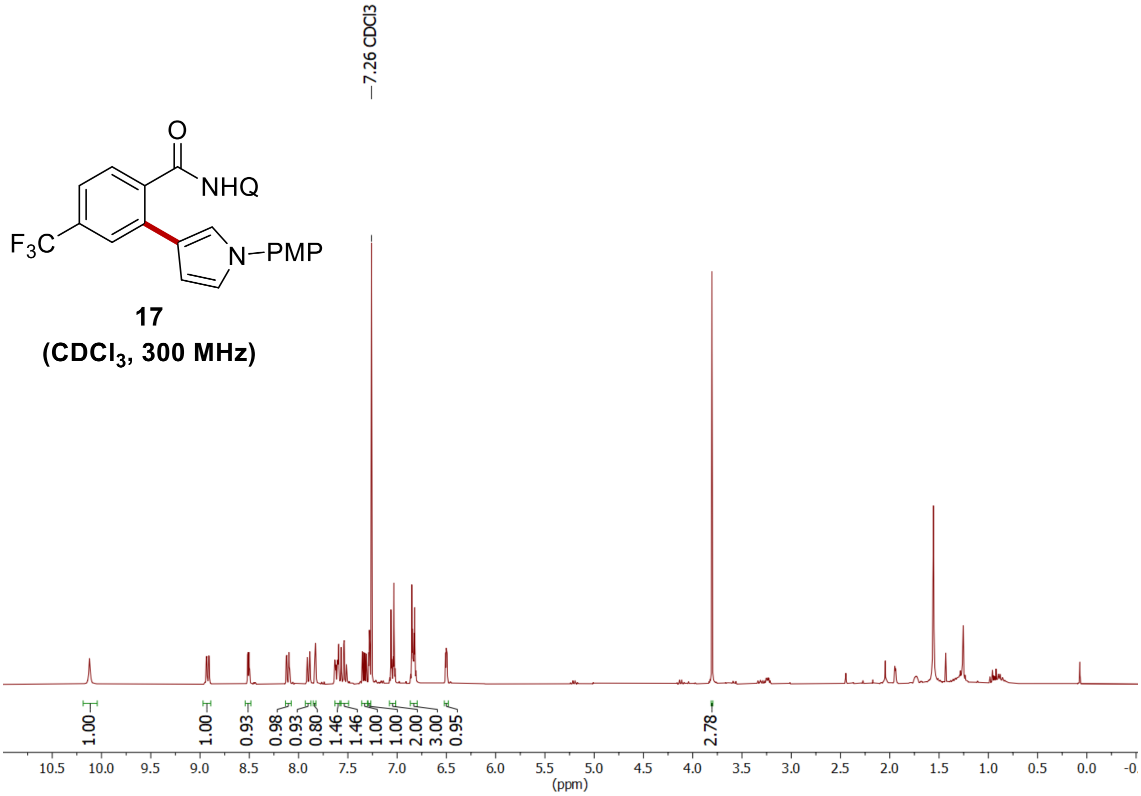

7.26 CDCl3
NHQ
F3C
N–PMP
17
(CDCl3, 300 MHz)
1.00
1.00
0.93
0.98
0.93
0.80
1.46
1.46
1.00
1.00
2.00
3.00
0.95
2.78
10.5 10.0 9.5 9.0 8.5 8.0 7.5 7.0 6.5 6.0 5.5 5.0 4.5 4.0 3.5 3.0 2.5 2.0 1.5 1.0 0.5 0.0
(ppm)

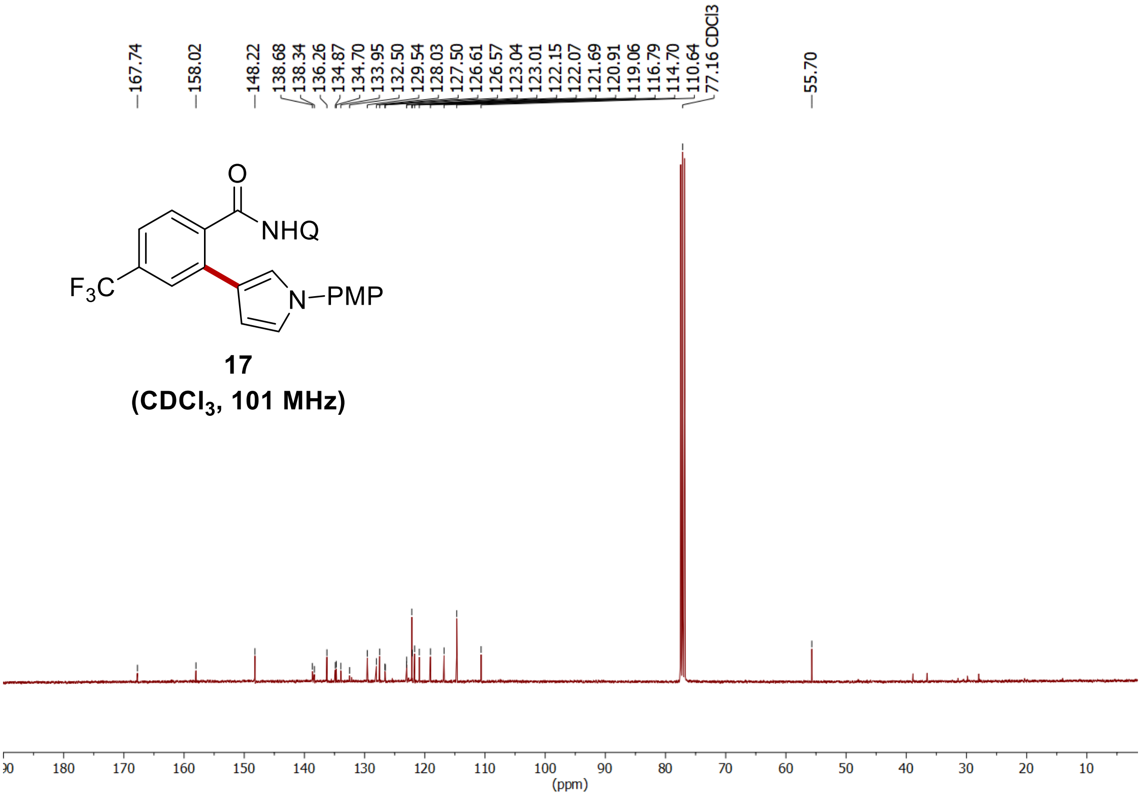

167.74
158.02
148.22
138.68
138.34
136.26
134.87
134.70
133.95
132.50
129.54
128.03
127.50
126.61
126.57
123.04
123.01
122.15
122.07
121.69
120.91
119.06
116.79
114.70
110.64
77.16 CDCl3
55.70
NHQ
F3C
N–PMP
17
(CDCl3, 101 MHz)
180 170 160 150 140 130 120 110 100 90 80 70 60 50 40 30 20 10
(ppm)

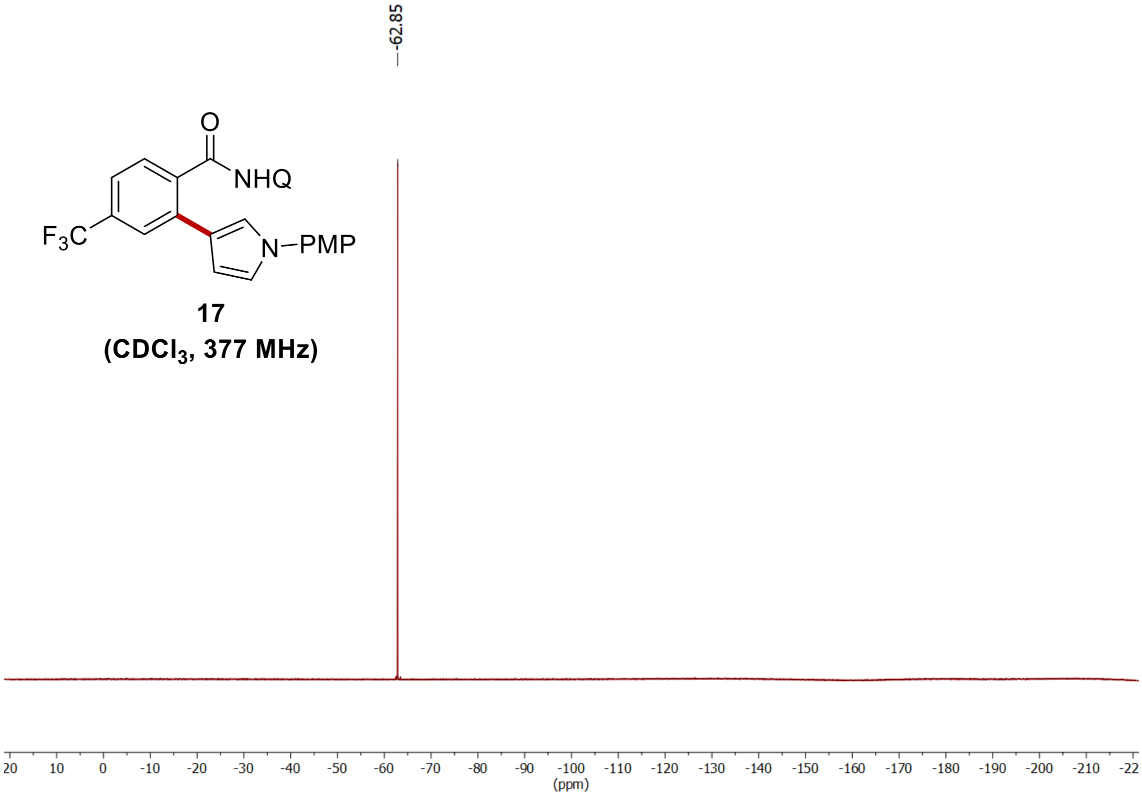

-62.85
O
NHQ
F_3C
N–PMP
17
($CDCl_3$, 377 MHz)
20 10 0 -10 -20 -30 -40 -50 -60 -70 -80 -90 -100 -110 -120 -130 -140 -150 -160 -170 -180 -190 -200 -210 -22
(ppm)

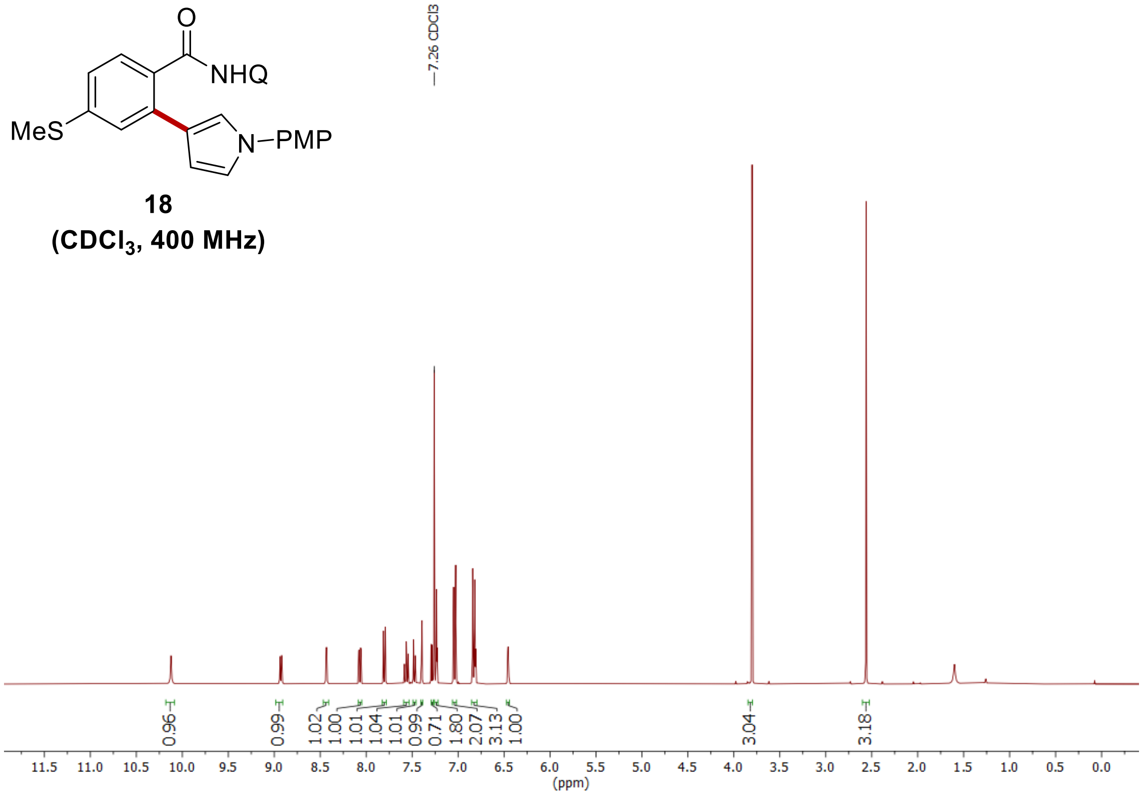

7.26 CDCl3
O
NHQ
MeS
N–PMP
18
($CDCl_3$, 400 MHz)
0.96
0.99
1.02
1.00
1.01
1.04
1.01
0.99
0.71
1.80
2.07
3.13
1.00
3.04
3.18
11.5 11.0 10.5 10.0 9.5 9.0 8.5 8.0 7.5 7.0 6.5 6.0 5.5 5.0 4.5 4.0 3.5 3.0 2.5 2.0 1.5 1.0 0.5 0.0
(ppm)

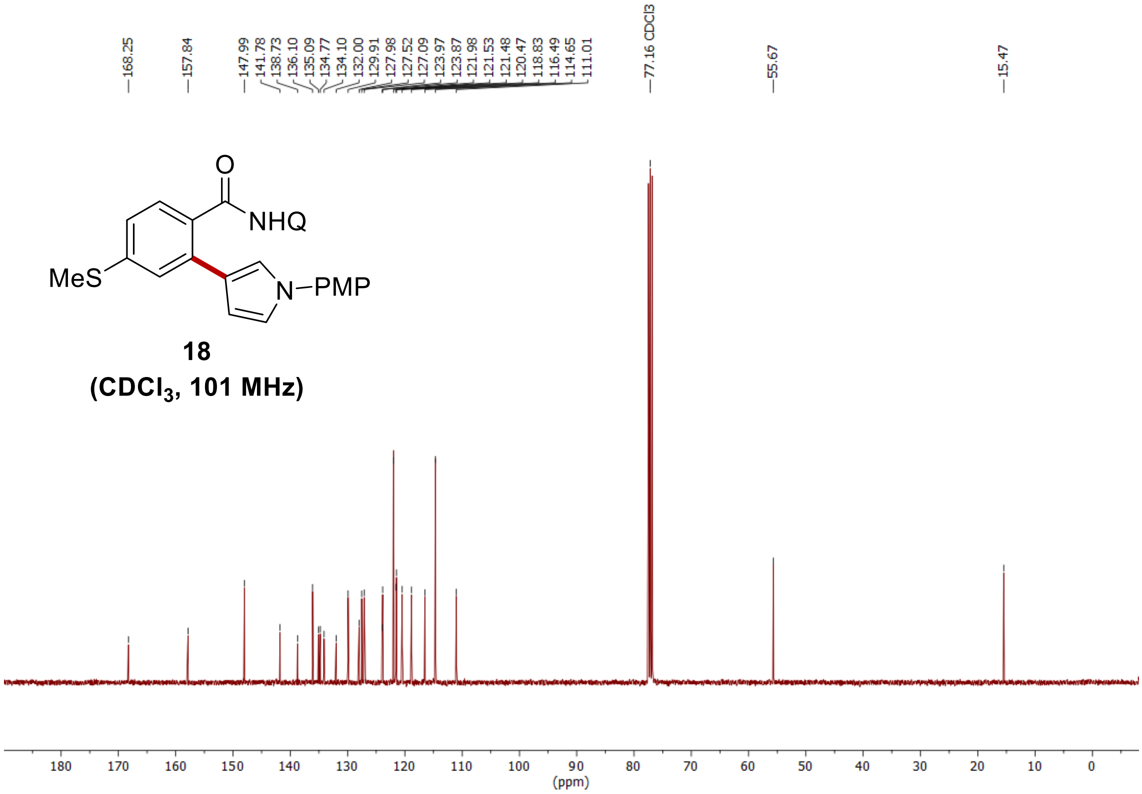
168.25
157.84
147.99
141.78
138.73
136.10
135.09
134.77
134.10
132.00
129.91
127.98
127.52
127.09
123.97
123.87
121.98
121.53
121.48
120.47
118.83
116.49
114.65
111.01
77.16 CDCl3
55.67
15.47
O
NHQ
MeS
N−PMP
18
(CDCl3, 101 MHz)
(ppm)

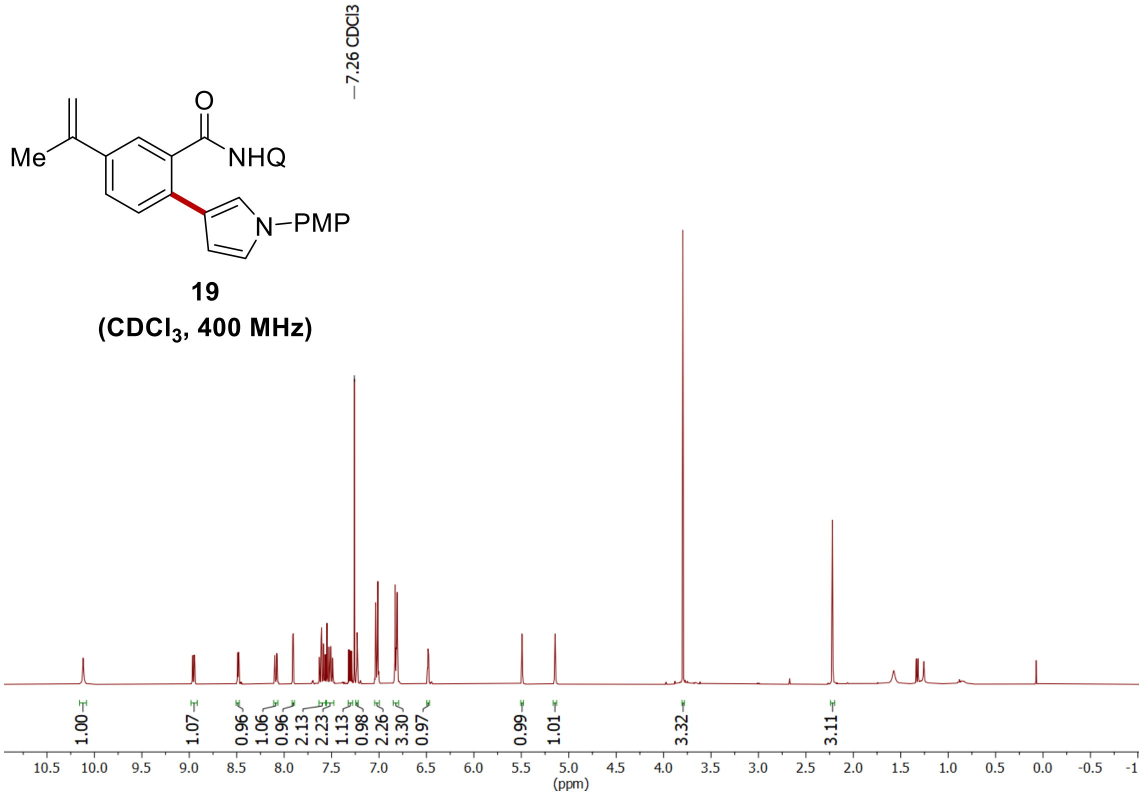
7.26 CDCl3
O
Me
NHQ
N−PMP
19
(CDCl3, 400 MHz)
1.00
1.07
0.96
1.06
0.96
2.13
2.23
1.13
0.98
2.26
3.30
0.97
0.99
1.01
3.32
3.11
(ppm)

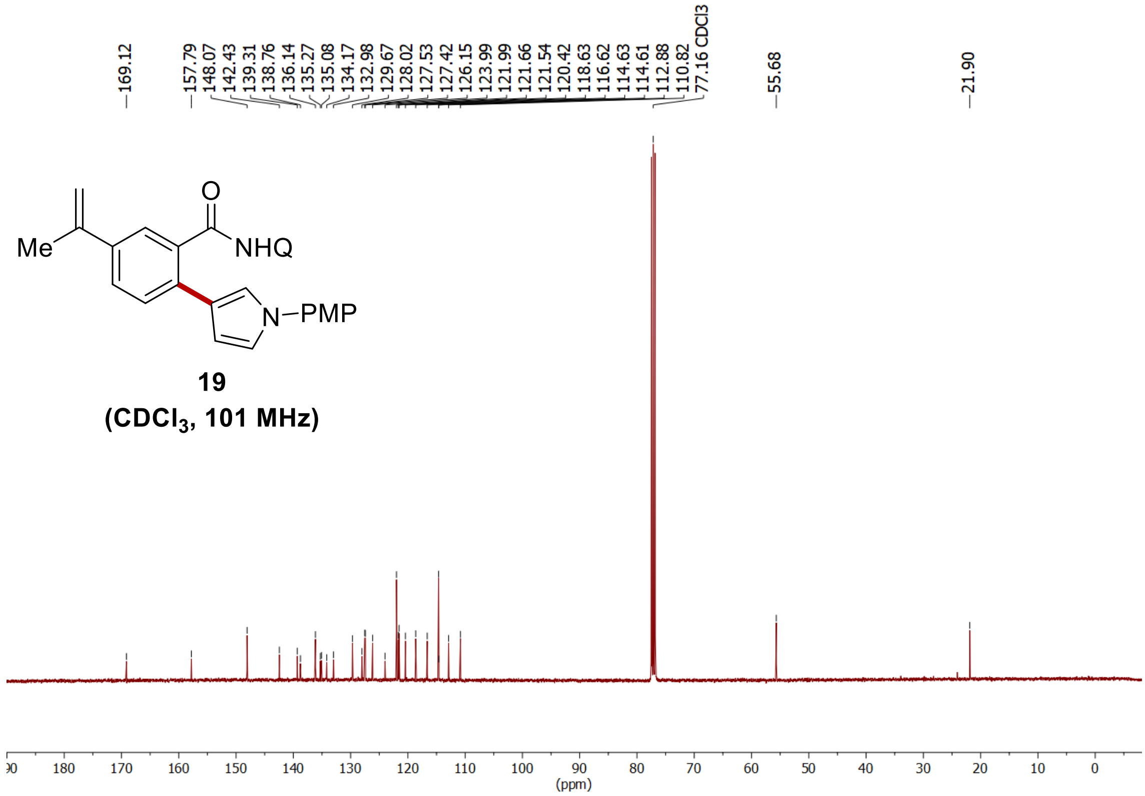

19
($CDCl_3$, 101 MHz)

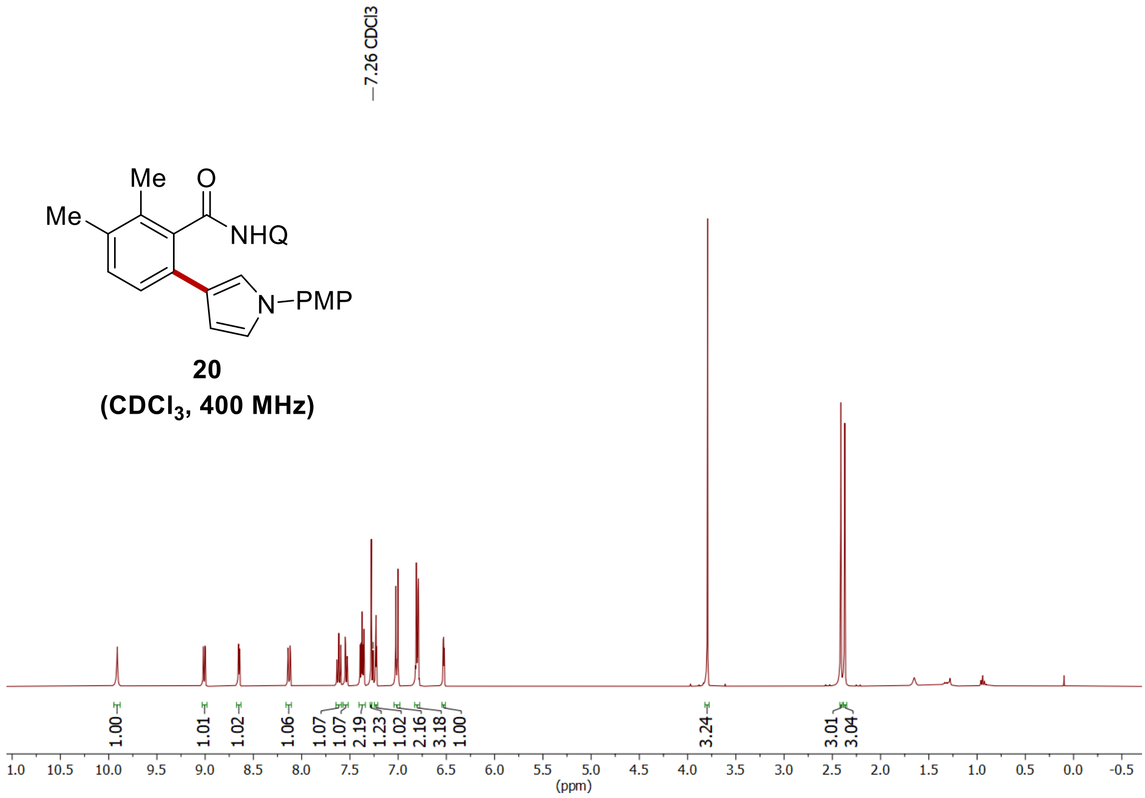

20
($CDCl_3$, 400 MHz)

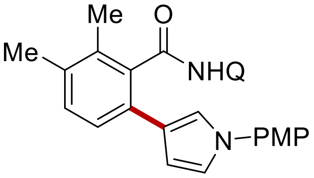

20
(CDCl$_3$, 101 MHz)

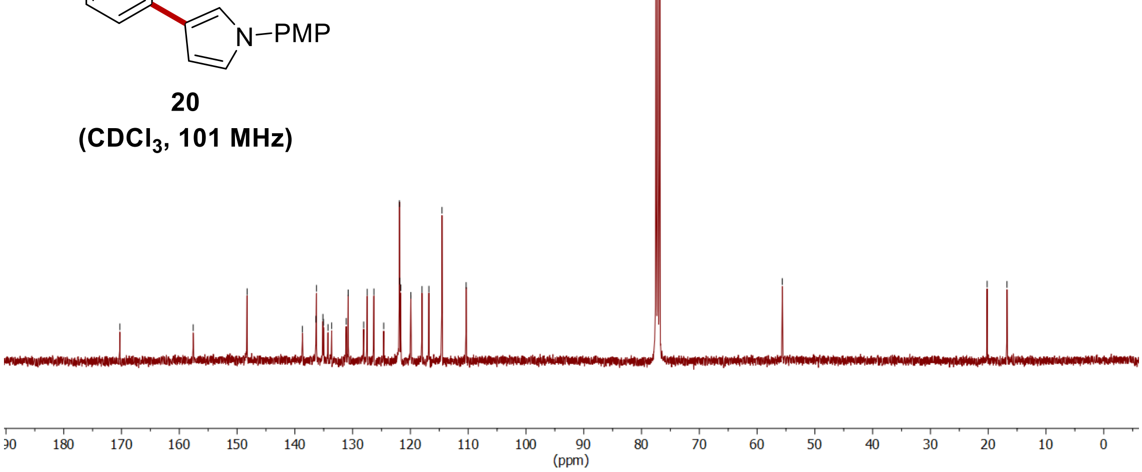

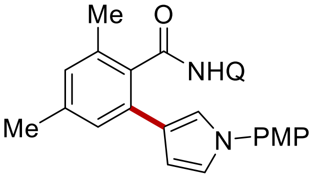

21
(CDCl$_3$, 400 MHz)

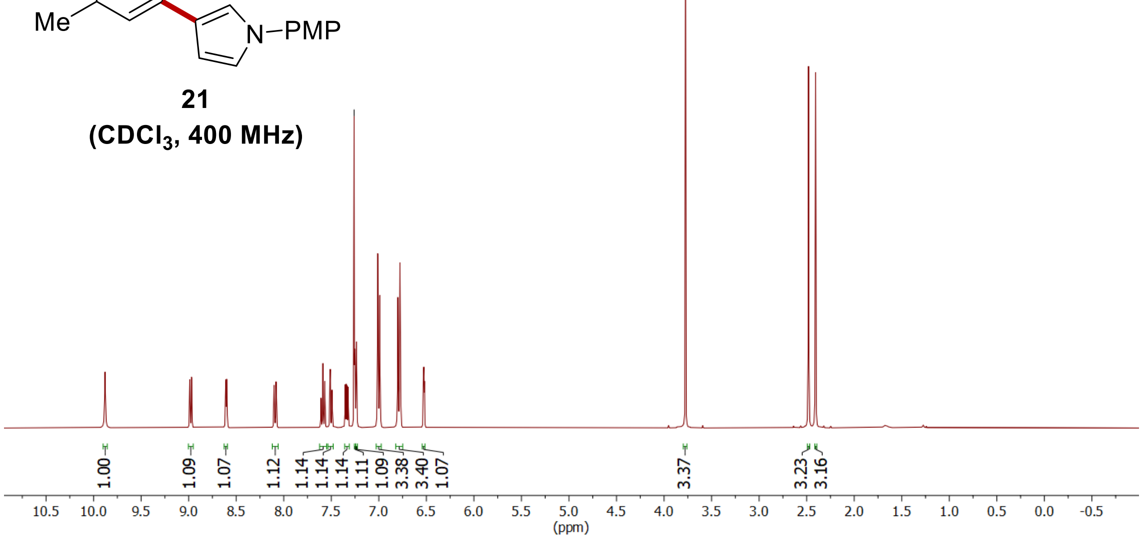

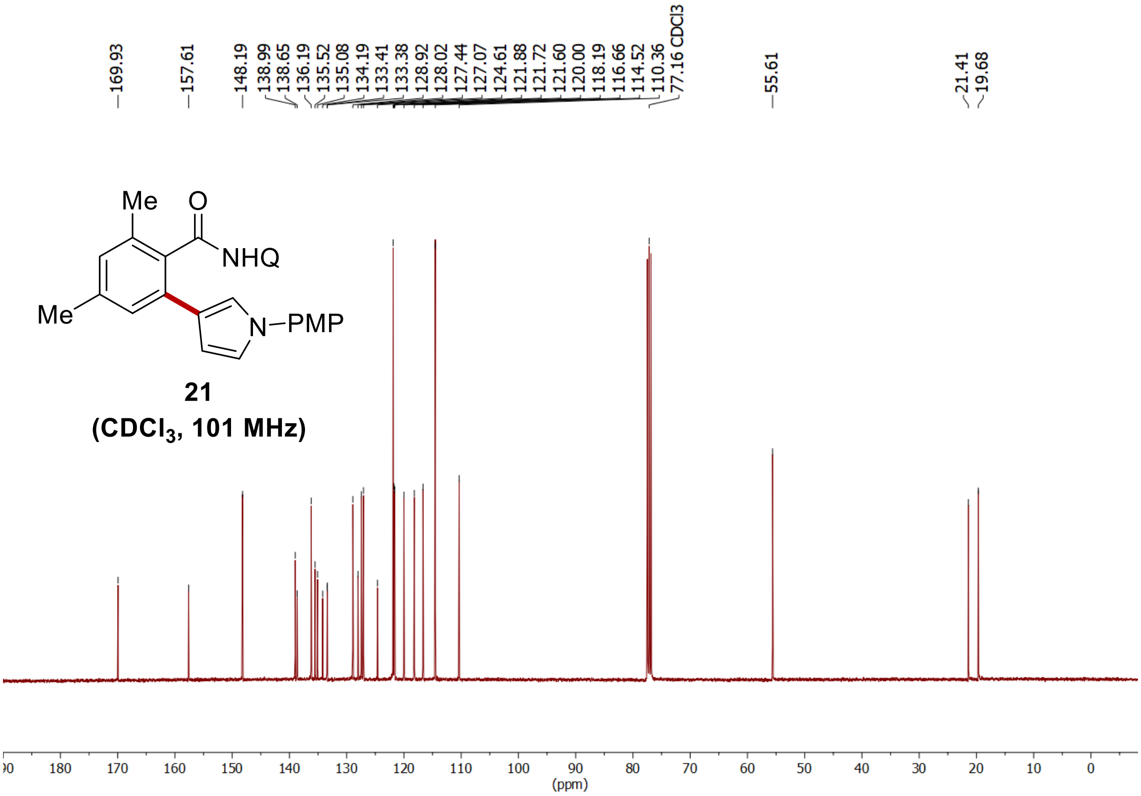
169.93
157.61
148.19
138.99
138.65
136.19
135.52
135.08
134.19
133.41
133.38
128.92
128.02
127.44
127.07
124.61
121.88
121.72
121.60
120.00
118.19
116.66
114.52
110.36
77.16 CDCl3
55.61
21.41
19.68
Me
O
NHQ
Me
N–PMP
21
(CDCl3, 101 MHz)
(ppm)

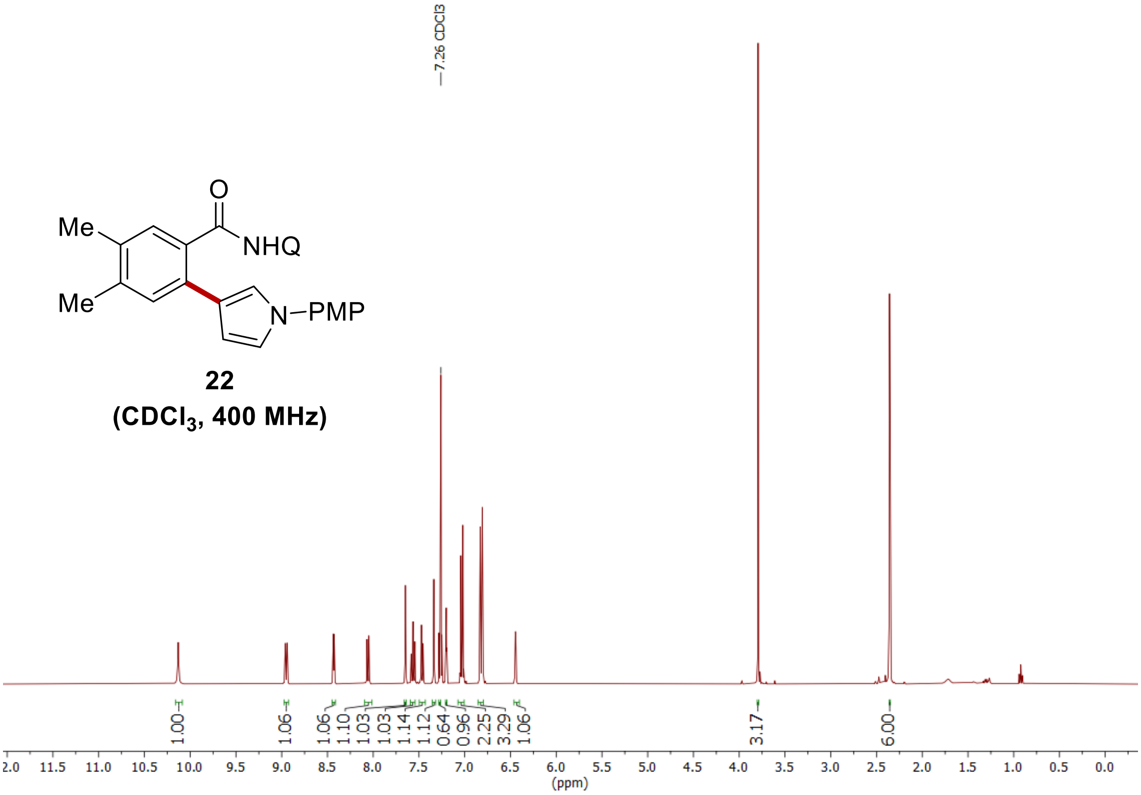
7.26 CDCl3
O
Me
NHQ
Me
N–PMP
22
(CDCl3, 400 MHz)
1.00
1.06
1.06
1.10
1.03
1.03
1.14
1.12
0.64
0.96
2.25
3.29
1.06
3.17
6.00
(ppm)

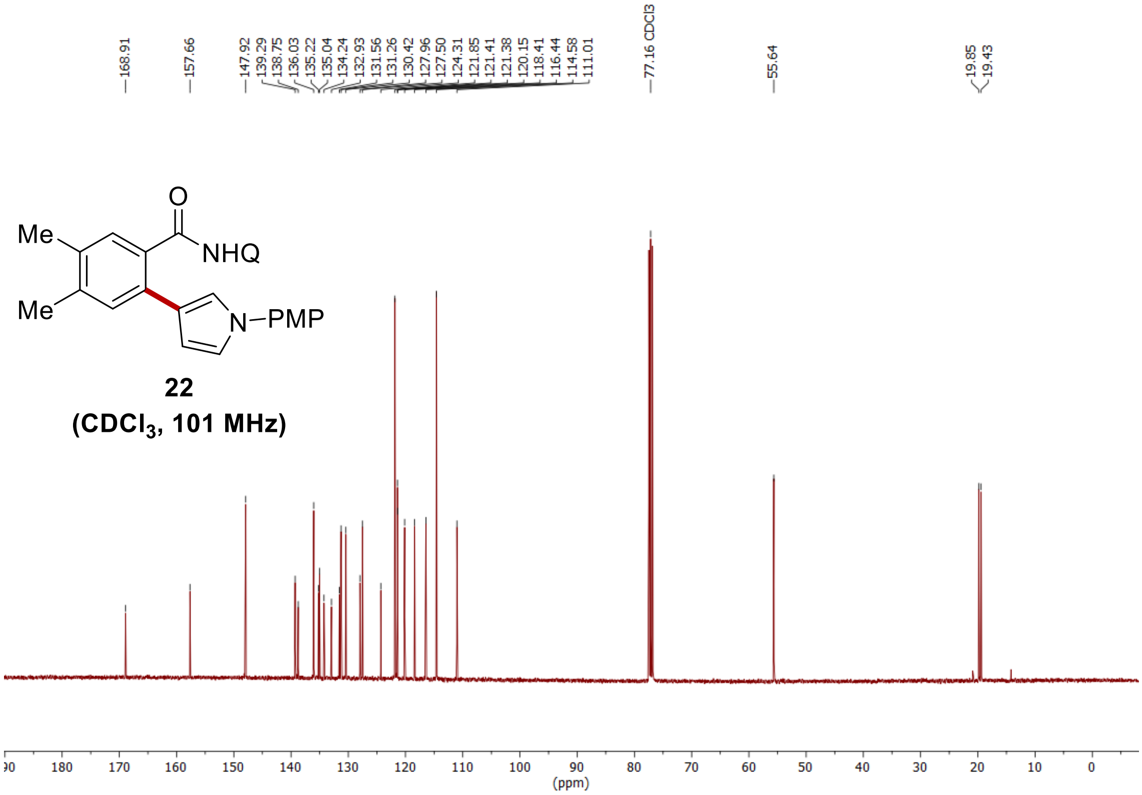
168.91
157.66
147.92
139.29
138.75
136.03
135.22
135.04
134.24
132.93
131.56
131.26
130.42
127.96
127.50
124.31
121.85
121.41
121.38
120.15
118.41
116.44
114.58
111.01
77.16 CDCl3
55.64
19.85
19.43
Me
Me
O
NHQ
N–PMP
22
($CDCl_3$, 101 MHz)
(ppm)

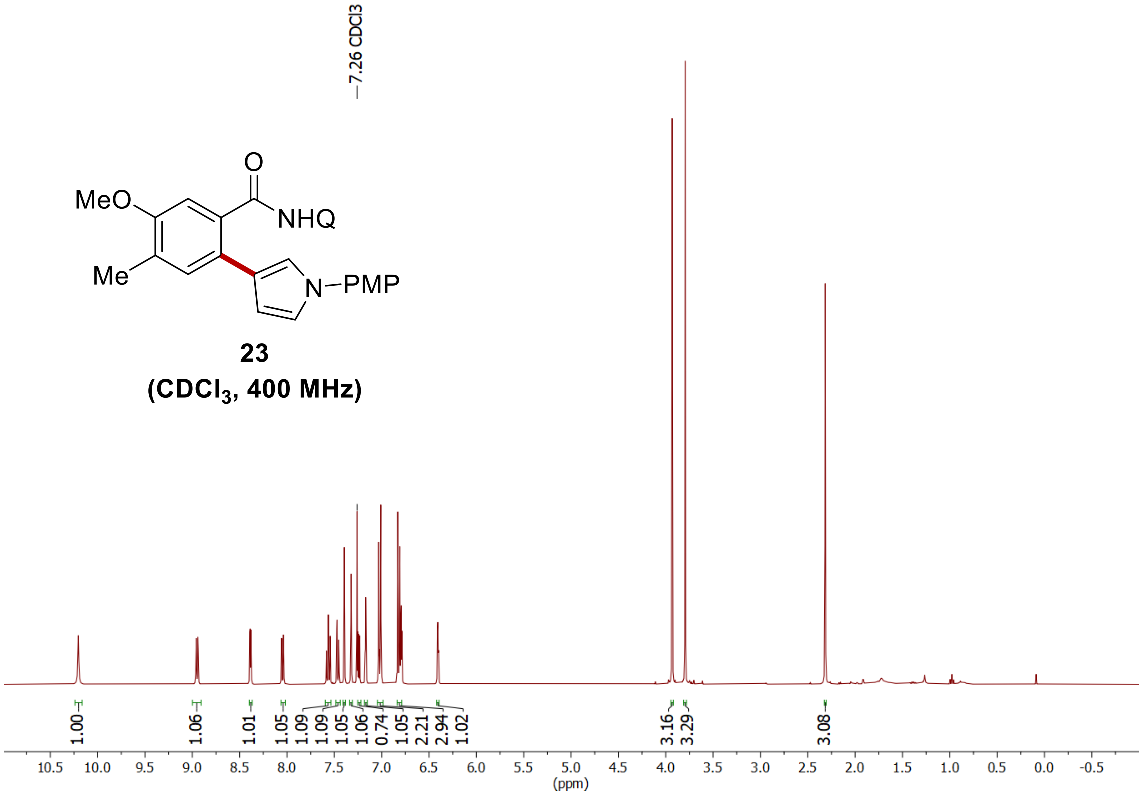
7.26 CDCl3
MeO
Me
O
NHQ
N–PMP
23
($CDCl_3$, 400 MHz)
1.00
1.06
1.01
1.05
1.09
1.09
1.05
1.06
0.74
1.05
2.21
2.94
1.02
3.16
3.29
3.08
(ppm)

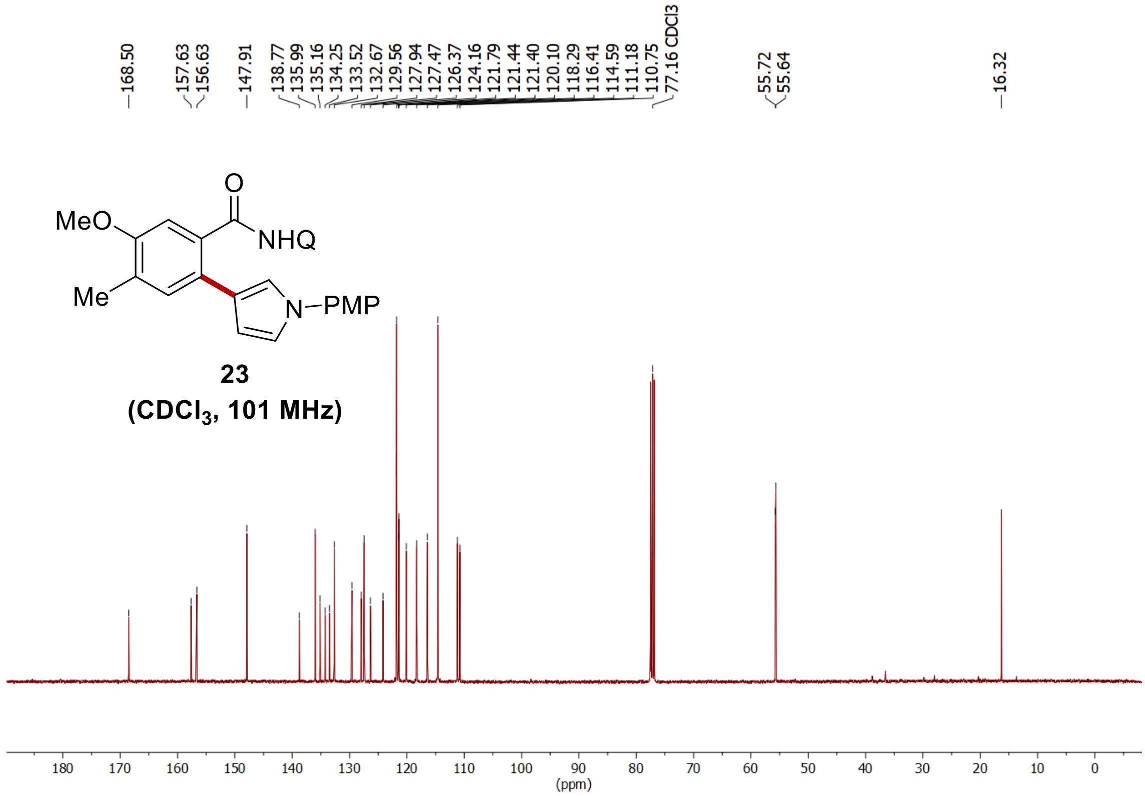
168.50
157.63
156.63
147.91
138.77
135.99
135.16
134.25
133.52
132.67
129.56
127.94
127.47
126.37
124.16
121.79
121.44
121.40
120.10
118.29
116.41
114.59
111.18
110.75
77.16 CDCl3
55.72
55.64
16.32
MeO
O
NHQ
Me
N−PMP
23
(CDCl$_3$, 101 MHz)
180 170 160 150 140 130 120 110 100 90 80 70 60 50 40 30 20 10 0
(ppm)

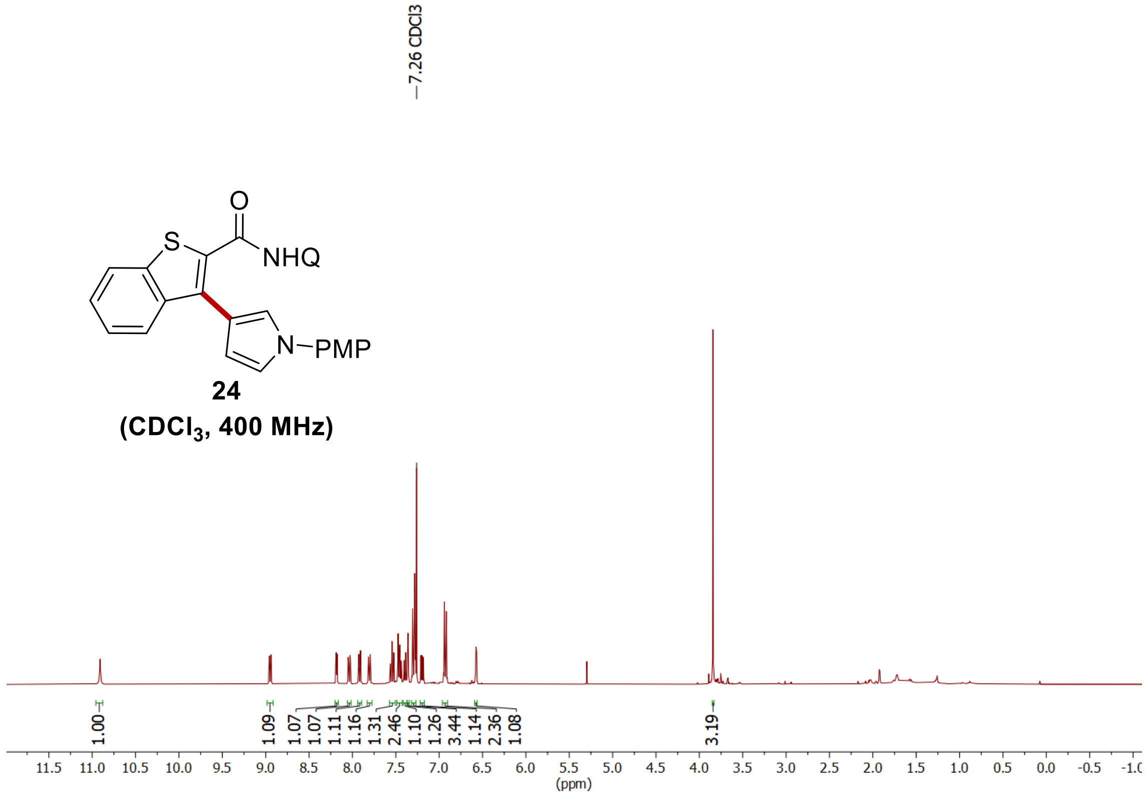
7.26 CDCl3
O
S
NHQ
N−PMP
24
(CDCl$_3$, 400 MHz)
1.00
1.09
1.07
1.07
1.11
1.16
1.31
2.46
1.10
1.26
3.44
1.14
2.36
1.08
3.19
11.5 11.0 10.5 10.0 9.5 9.0 8.5 8.0 7.5 7.0 6.5 6.0 5.5 5.0 4.5 4.0 3.5 3.0 2.5 2.0 1.5 1.0 0.5 0.0 -0.5 -1.0
(ppm)

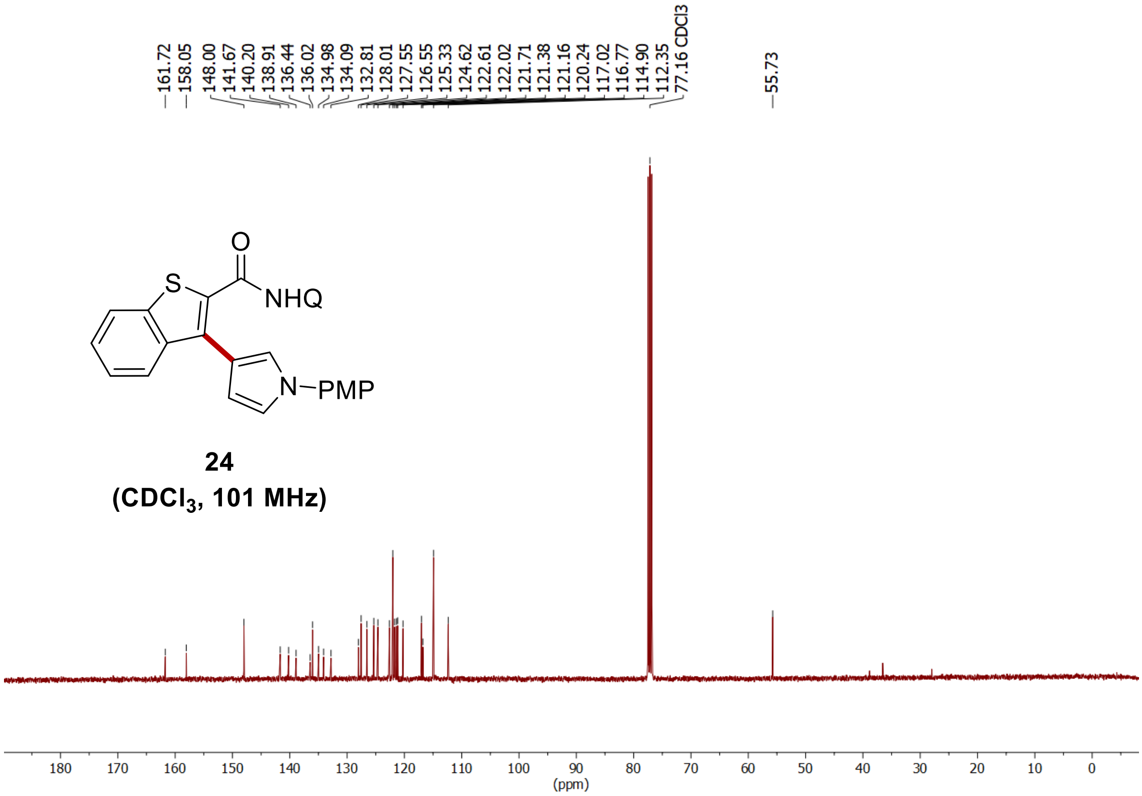

24
($CDCl_3$, 101 MHz)

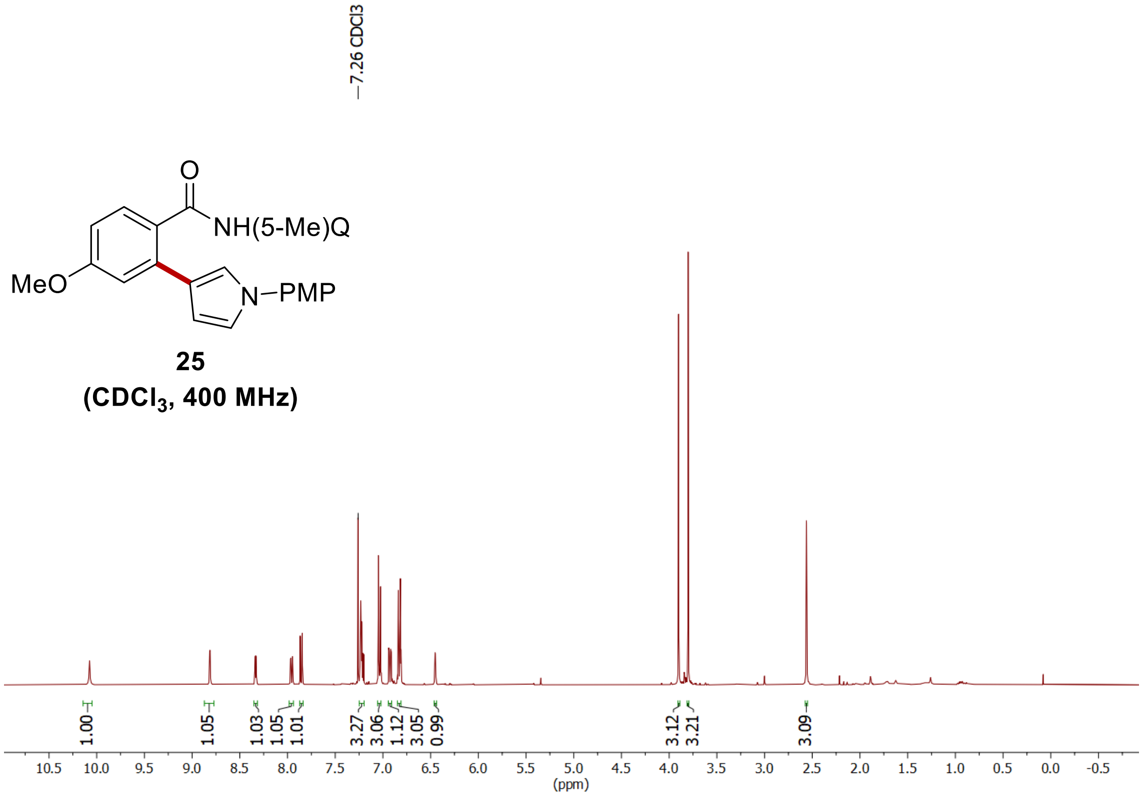

25
($CDCl_3$, 400 MHz)

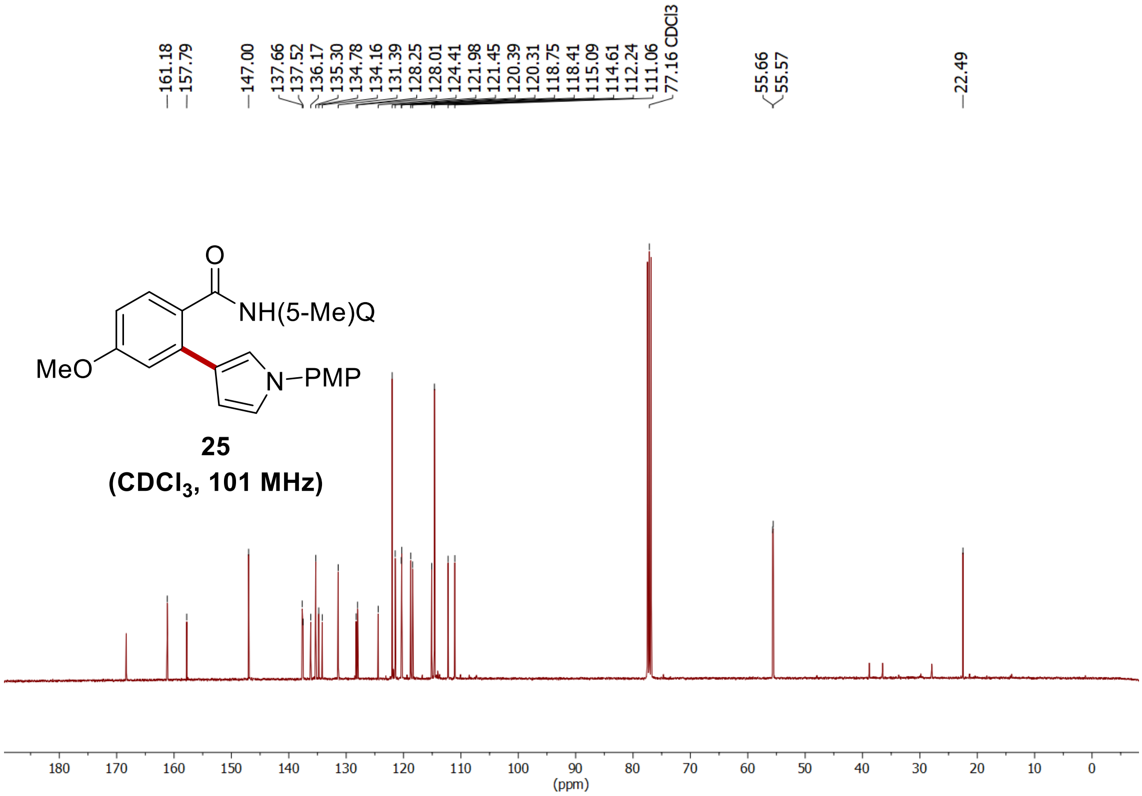

25
(CDCl$_3$, 101 MHz)

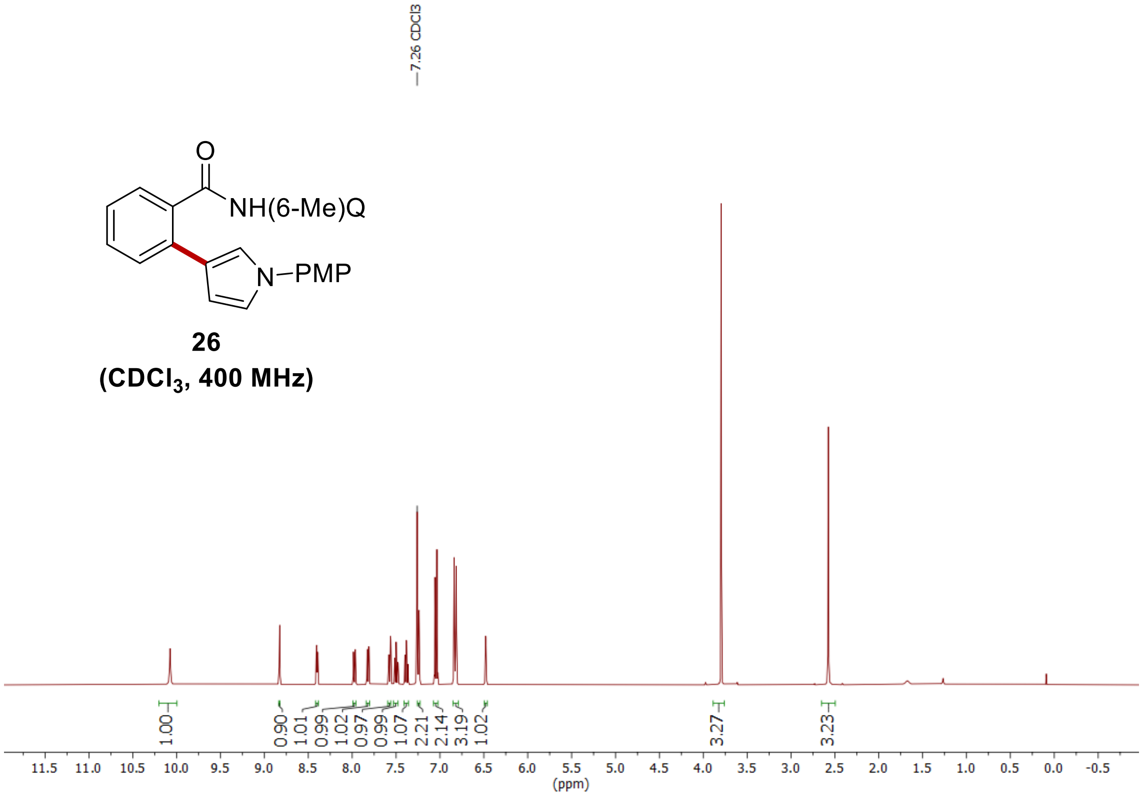

26
(CDCl$_3$, 400 MHz)

26
(CDCl$_3$, 101 MHz)

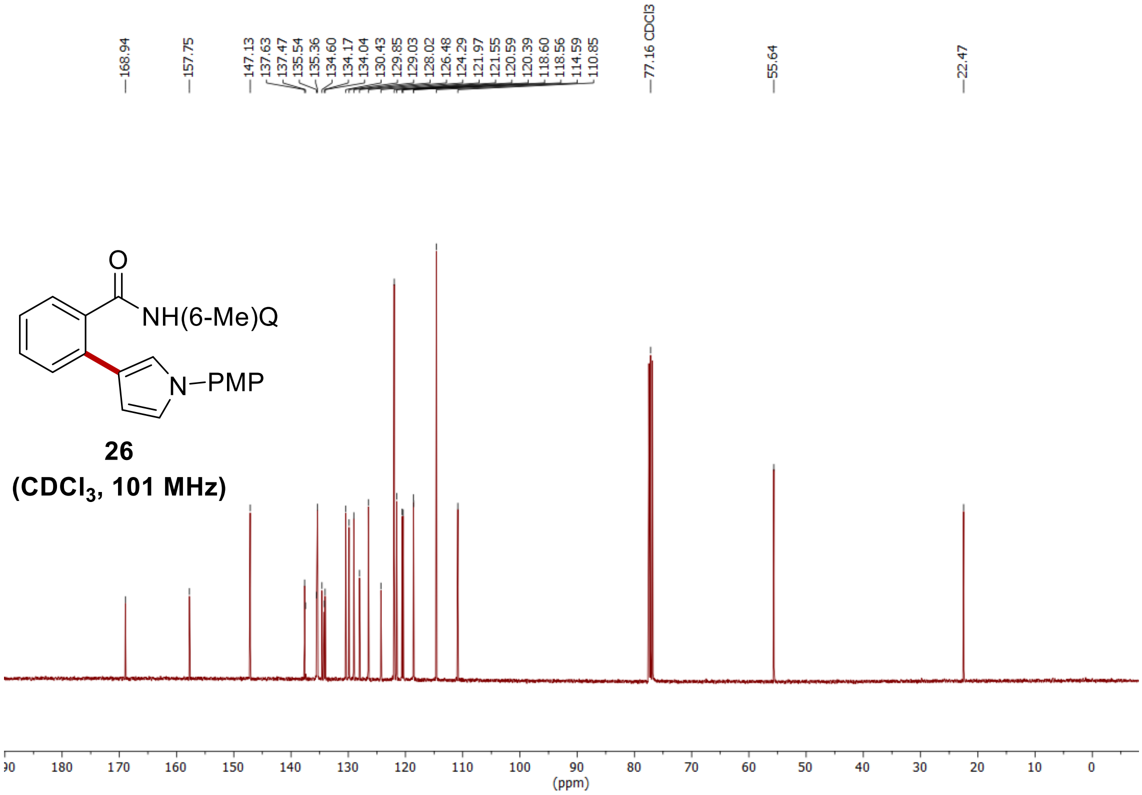

27
(CDCl$_3$, 400 MHz)

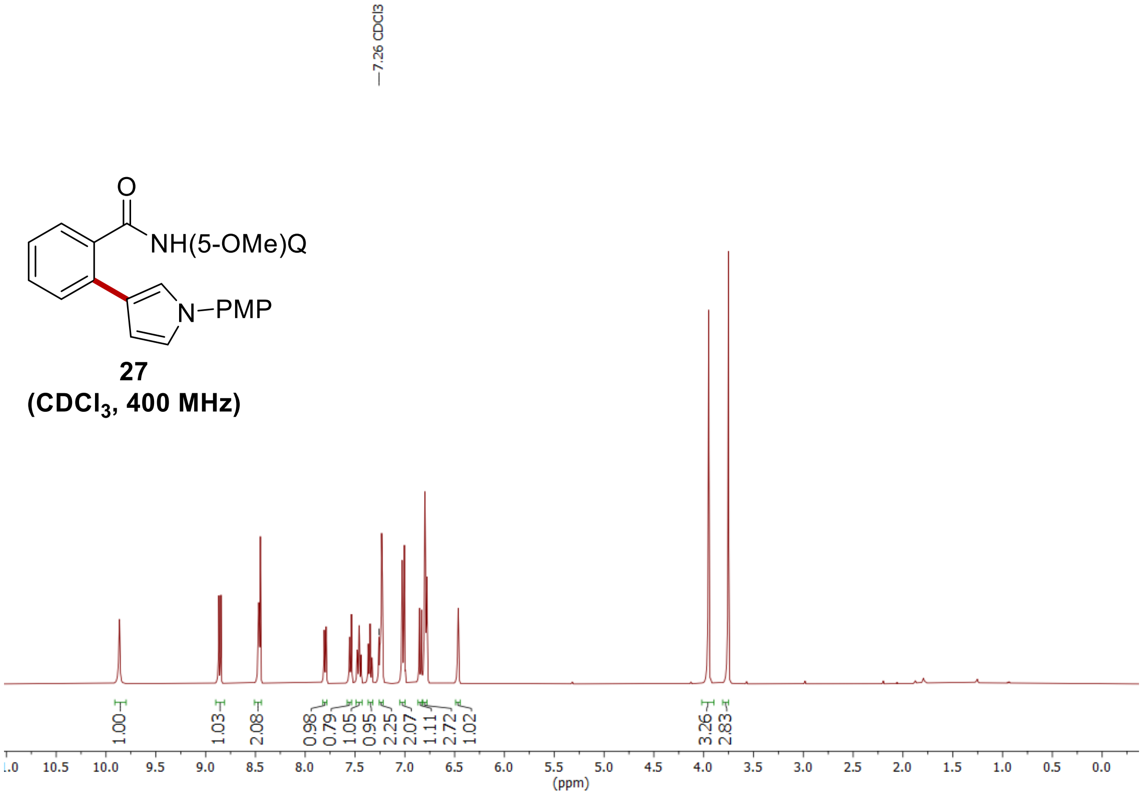

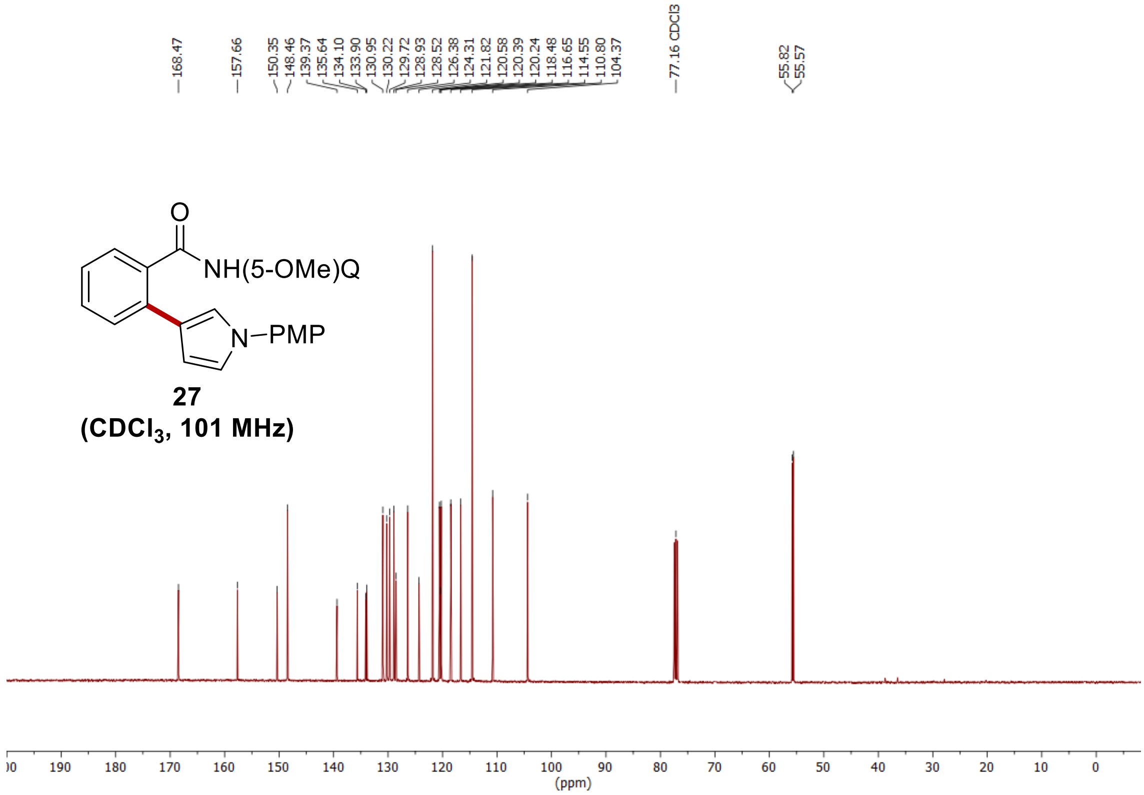
168.47
157.66
150.35
148.46
139.37
135.64
134.10
133.90
130.95
130.22
129.72
128.93
128.52
126.38
124.31
121.82
120.58
120.39
120.24
118.48
116.65
114.55
110.80
104.37
77.16 CDCl3
55.82
55.57
O
NH(5-OMe)Q
N−PMP
27
(CDCl3, 101 MHz)
(ppm)

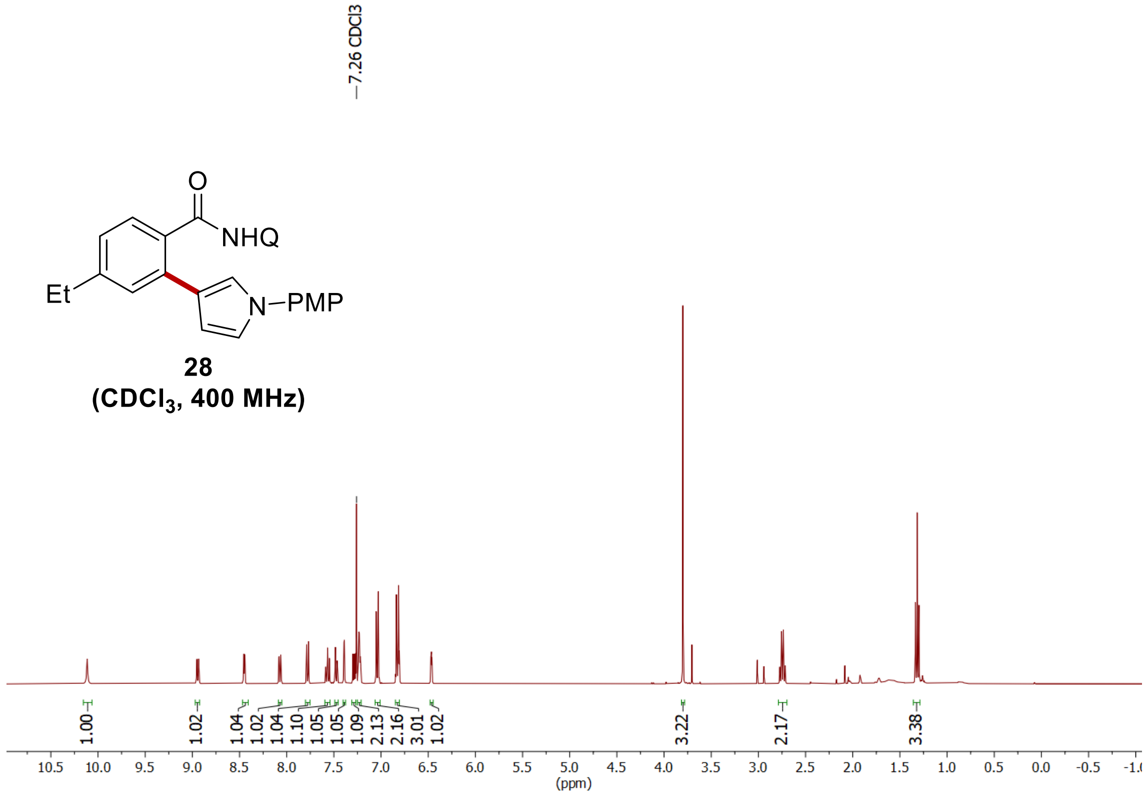
7.26 CDCl3
O
NHQ
Et
N−PMP
28
(CDCl3, 400 MHz)
1.00
1.02
1.04
1.02
1.04
1.10
1.05
1.05
1.09
2.13
2.16
3.01
1.02
3.22
2.17
3.38
(ppm)

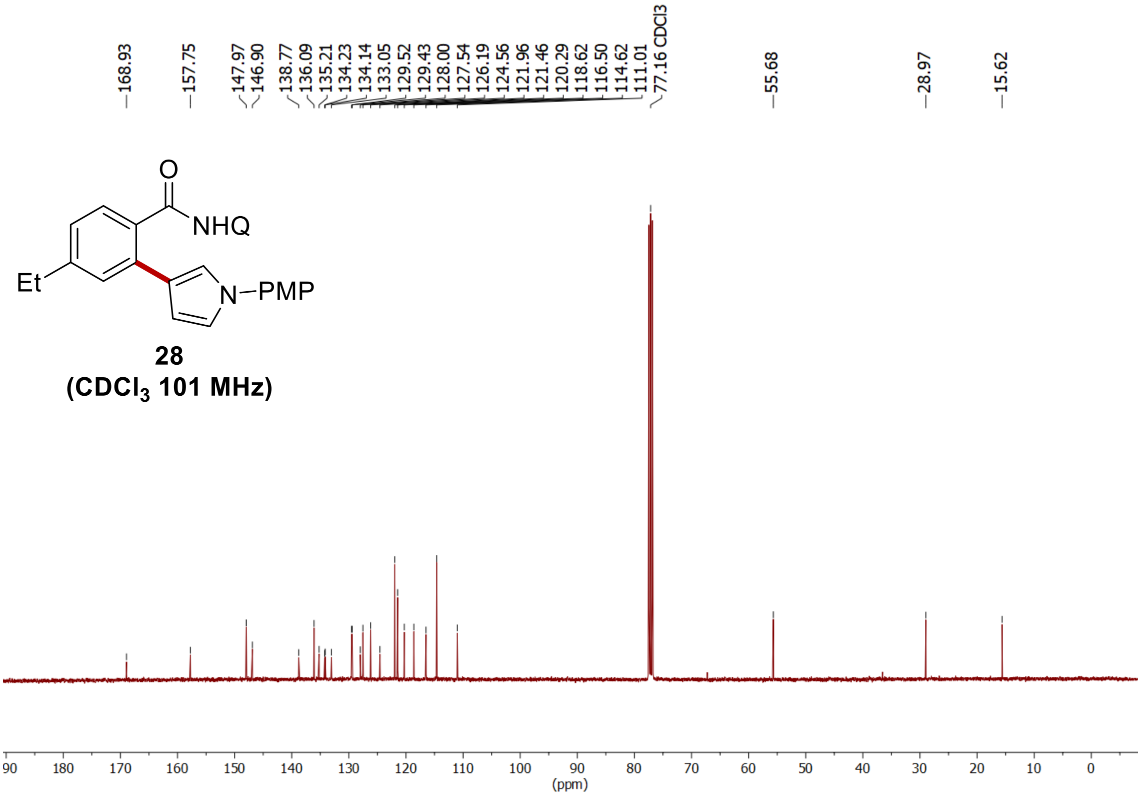

28
($CDCl_3$ 101 MHz)

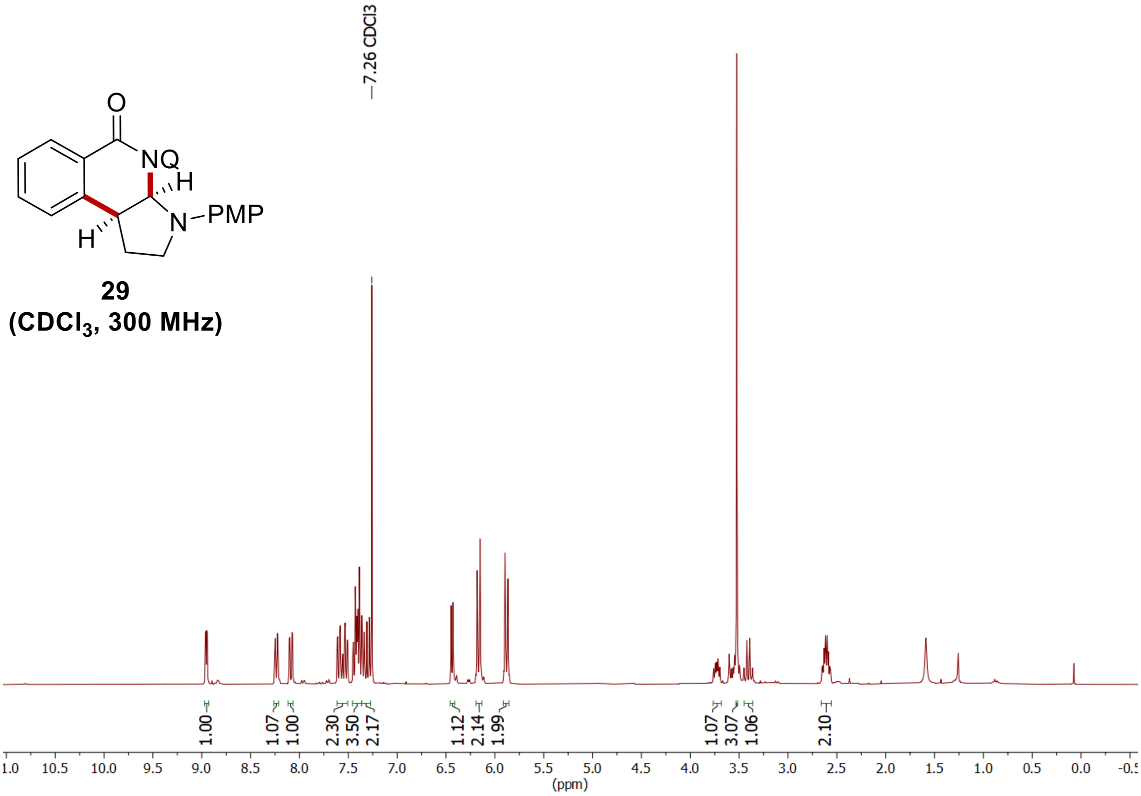

29
($CDCl_3$, 300 MHz)

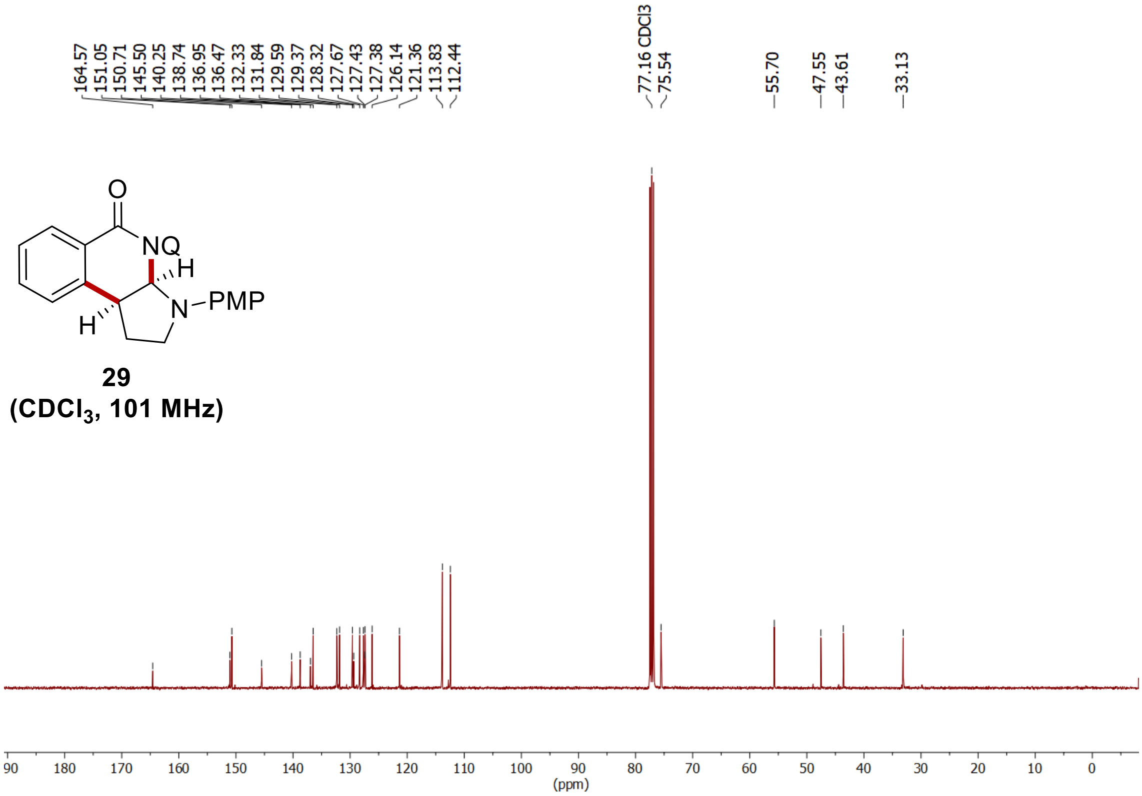

29
($CDCl_3$, 101 MHz)

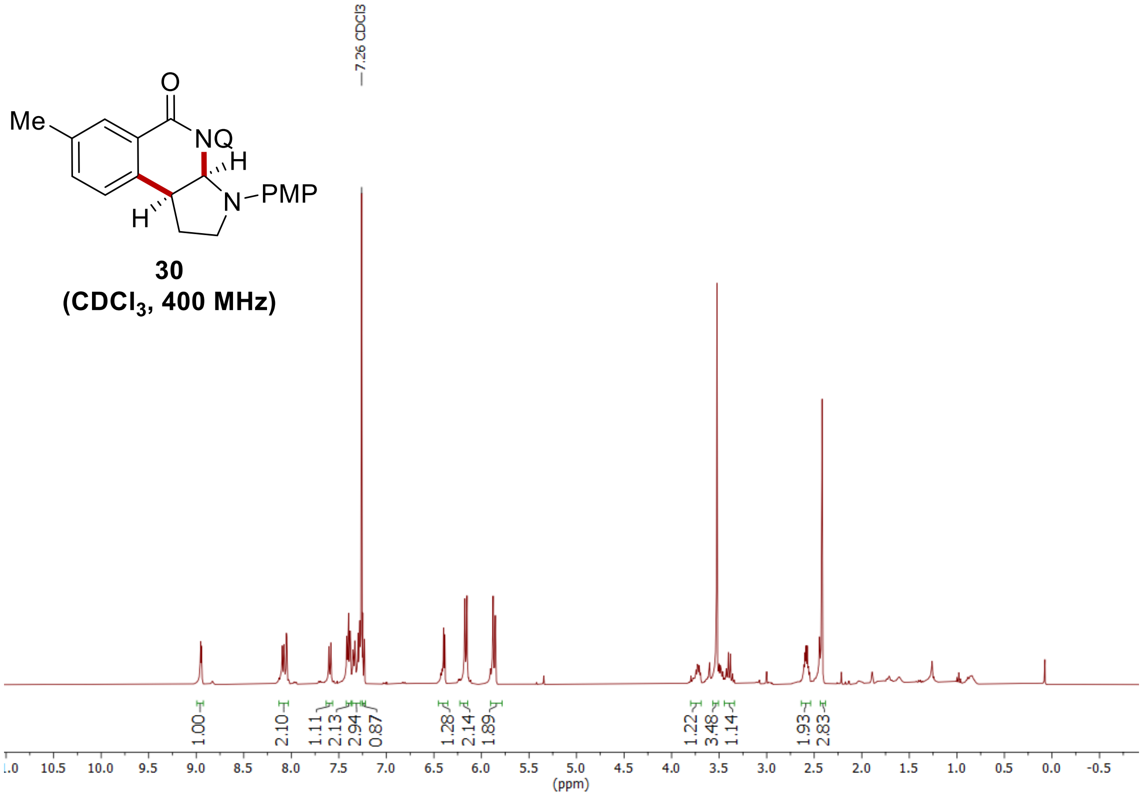

30
($CDCl_3$, 400 MHz)

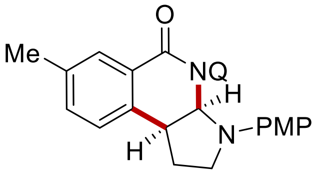

30
(CDCl$_3$, 101MHz)

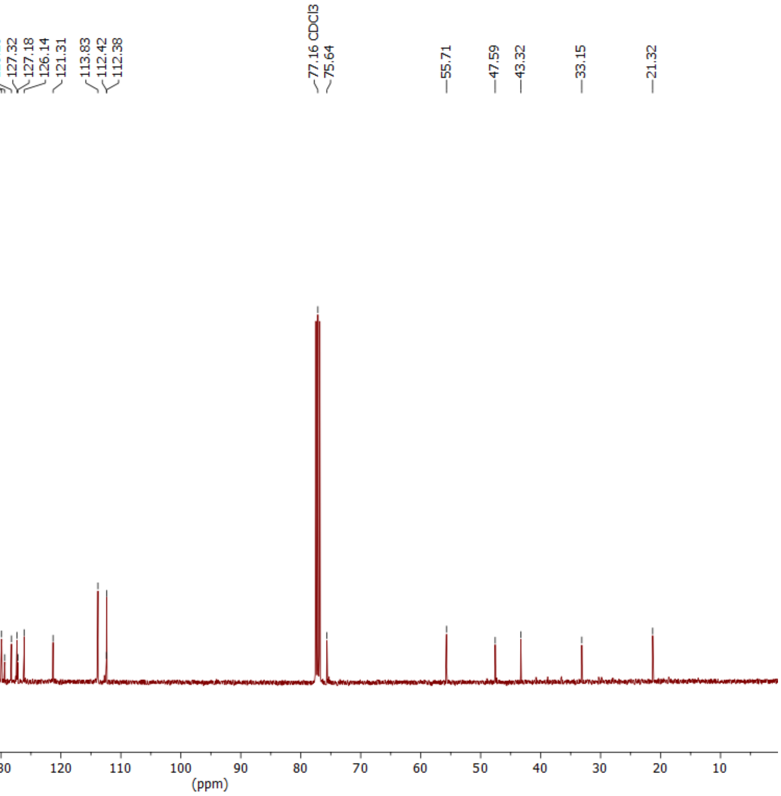

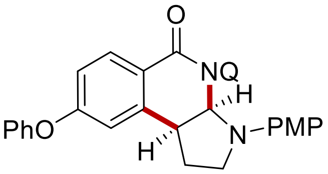

31
(CDCl$_3$, 400 MHz)

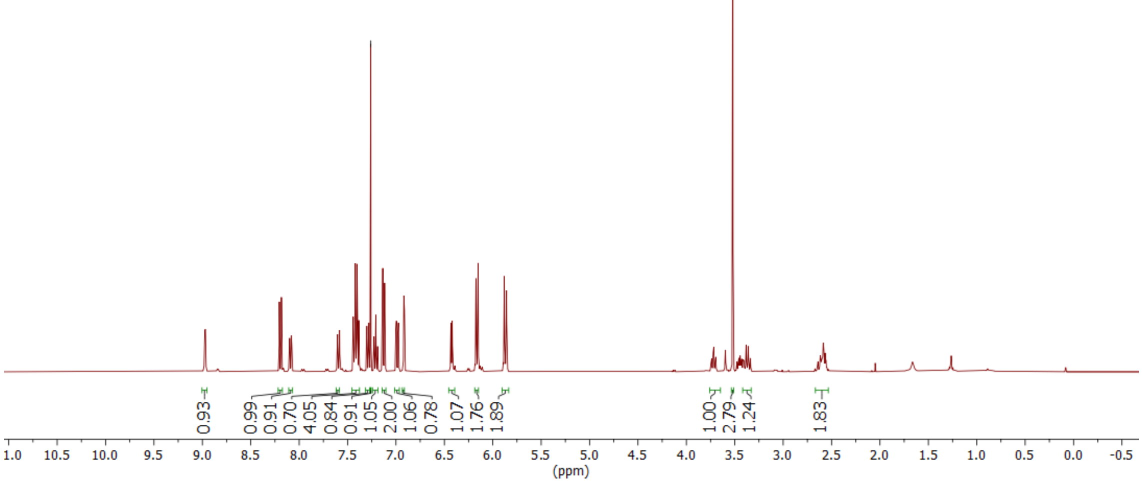

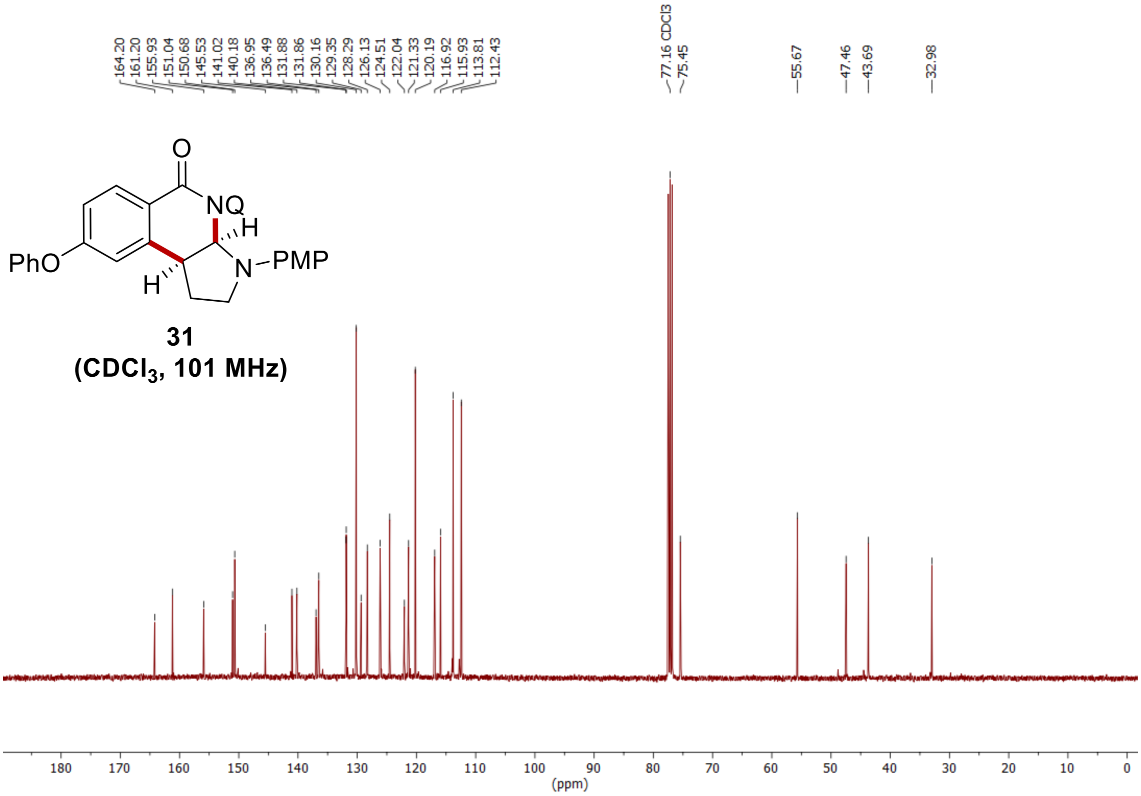
164.20
161.20
155.93
151.04
150.68
145.53
141.02
140.18
136.95
136.49
131.88
131.86
130.16
129.35
128.29
126.13
124.51
122.04
121.33
120.19
116.92
115.93
113.81
112.43
77.16 CDCl3
75.45
55.67
47.46
43.69
32.98
O
NQ
H
PhO
N−PMP
H
31
(CDCl3, 101 MHz)
(ppm)

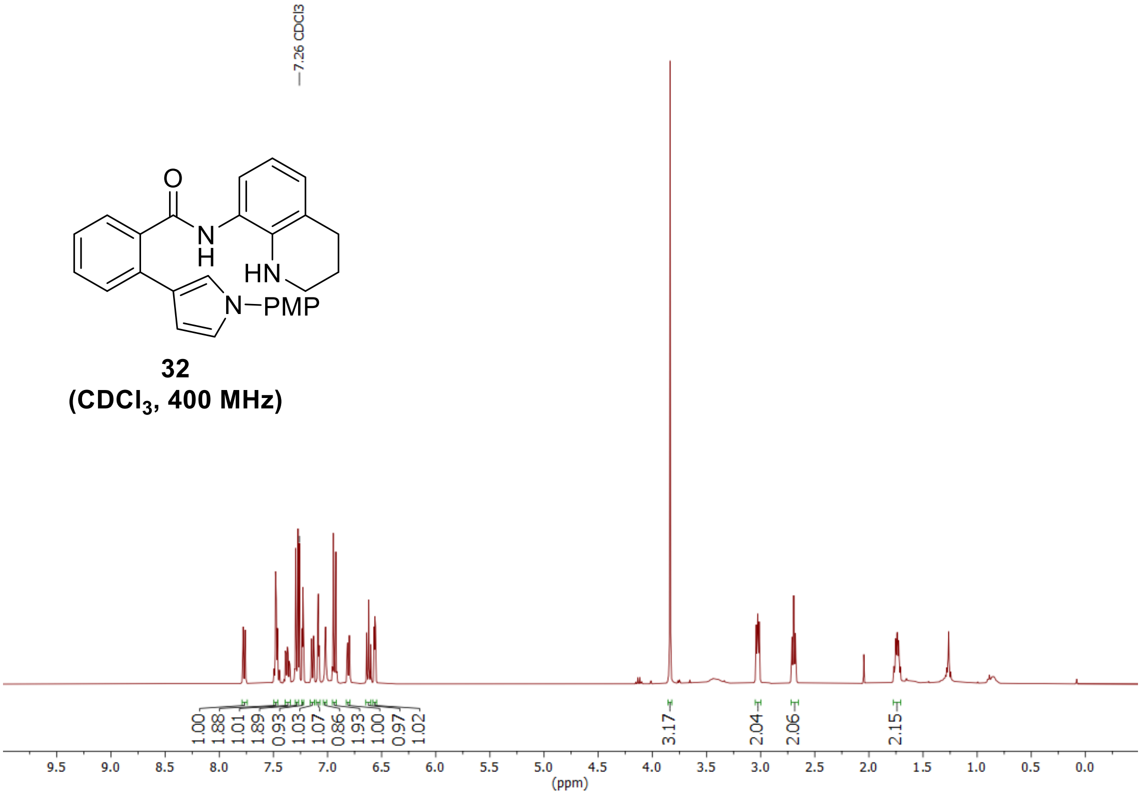
7.26 CDCl3
O
N
H
HN
N−PMP
32
(CDCl3, 400 MHz)
1.00
1.88
1.01
1.89
0.93
1.03
1.07
0.86
1.93
1.00
0.97
1.02
3.17
2.04
2.06
2.15
(ppm)

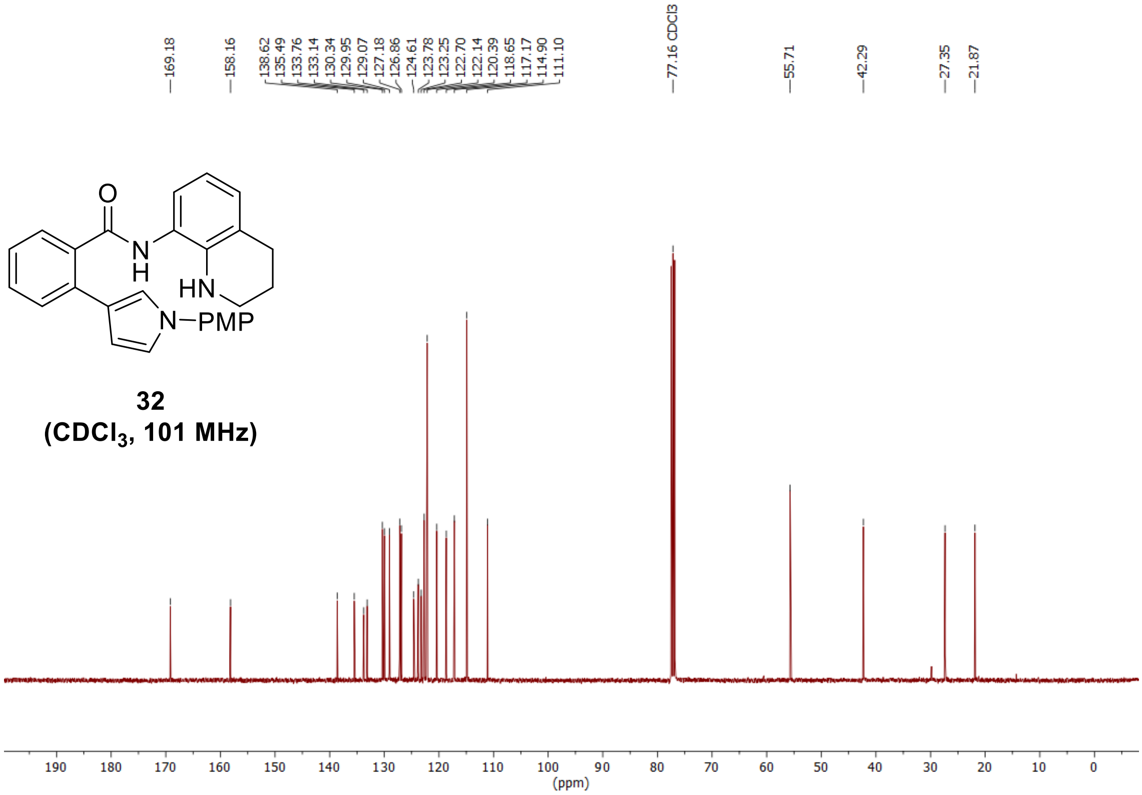
169.18
158.16
138.62
135.49
133.76
133.14
130.34
129.95
129.07
127.18
126.86
124.61
123.78
123.25
122.70
122.14
120.39
118.65
117.17
114.90
111.10
77.16 CDCl3
55.71
42.29
27.35
21.87
O
N
H
HN
N−PMP
32
(CDCl3, 101 MHz)
(ppm)

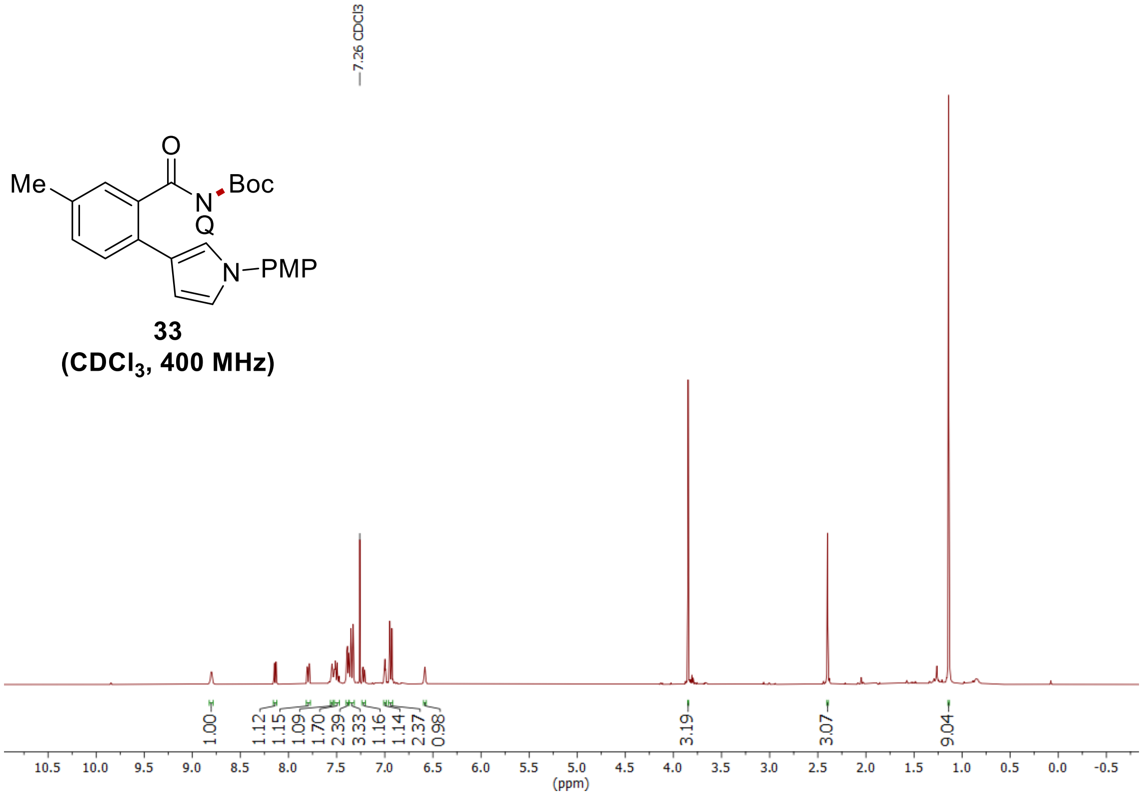
7.26 CDCl3
Me
O
N
Boc
Q
N−PMP
33
(CDCl3, 400 MHz)
1.00
1.12
1.15
1.09
1.70
2.39
3.33
1.16
1.14
2.37
0.98
3.19
3.07
9.04
(ppm)

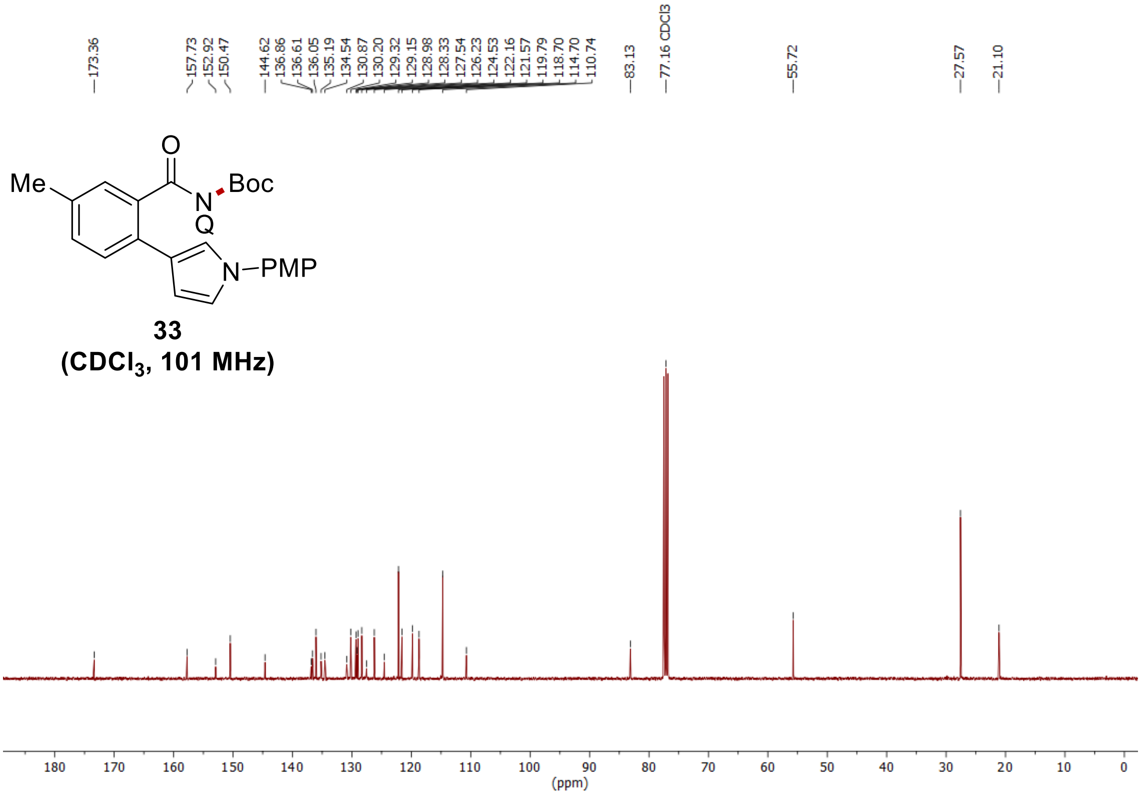

33
($CDCl_3$, 101 MHz)

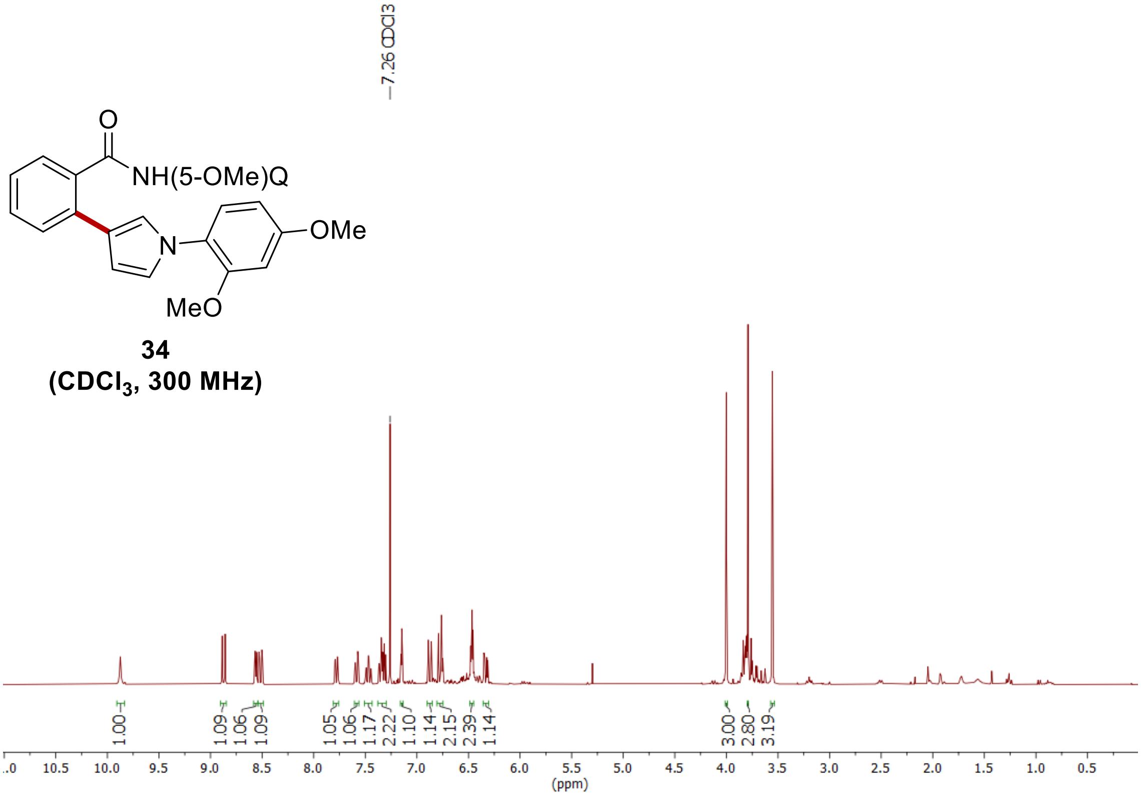

34
($CDCl_3$, 300 MHz)

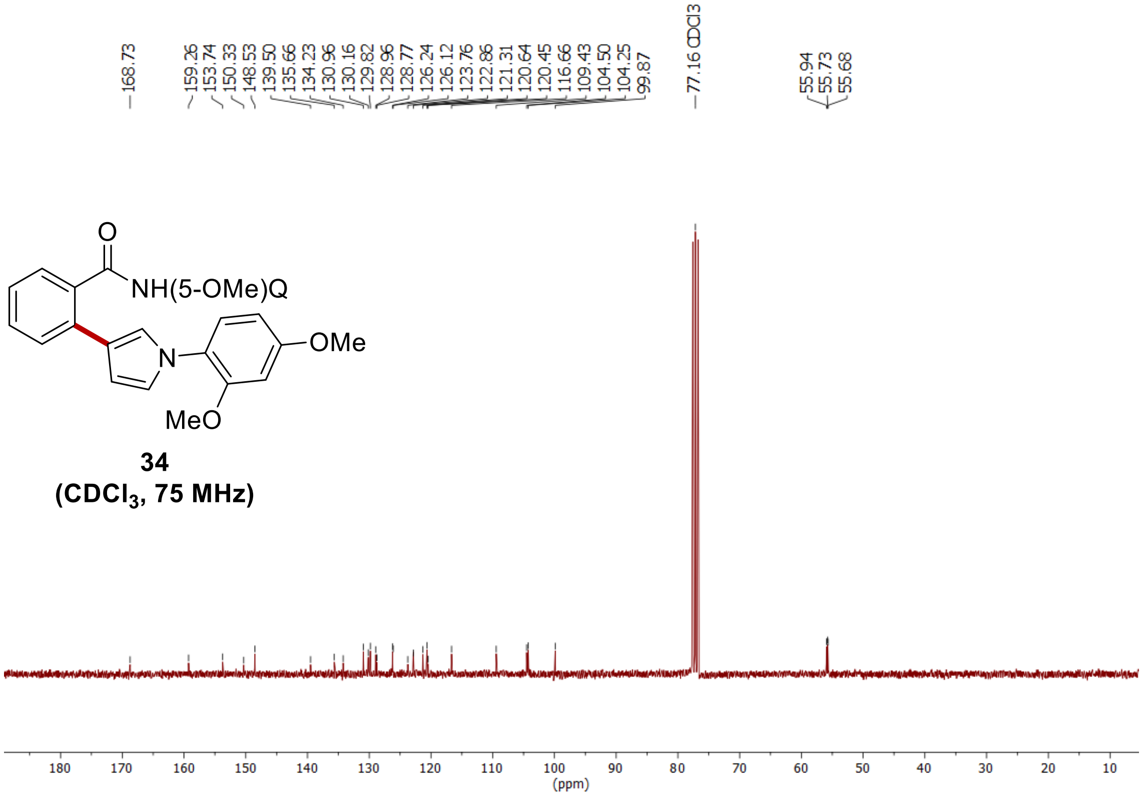
168.73
159.26
153.74
150.33
148.53
139.50
135.66
134.23
130.96
130.16
129.82
128.96
128.77
126.24
126.12
123.76
122.86
121.31
120.64
120.45
116.66
109.43
104.50
104.25
99.87
77.16 CDCl3
55.94
55.73
55.68
O
NH(5-OMe)Q
N
OMe
MeO
34
(CDCl3, 75 MHz)
180 170 160 150 140 130 120 110 100 90 80 70 60 50 40 30 20 10
(ppm)

XII. References

[1] J. Liu, S. Zhuang, Q. Gui, X. Chen, Z. Yang, Z. Tan, *Adv. Synth. Catal.* **2015**, *357*, 732–738.

[2] B. M. Monks, E. R. Fruchey, S. P. Cook, *Angew. Chem. Int. Ed.* **2014**, *53*, 11065–11069.

[3] C. J. Whiteoak, O. Planas, A. Company, X. Ribas, *Adv. Synth. Catal.* **2016**, *358*, 1679–1688.

[4] S. Kumar, S. Pradhan, S. Roy, P. B. De, T. Punniyamurthy, *J. Org. Chem.* **2019**, *84*, 10481–10489.

[5] L. Ruyet, T. Poisson, T. Besset, *Eur. J. Org. Chem.* **2021**, *2021*, 3407–3410.

[6] S. Liu, B. He, H. Li, X. Zhang, Y. Shang, W. Su, *Chem. Eur. J.* **2021**, *27*, 15628–15633.

[7] L. D. Tran, J. Roane, O. Daugulis, *Angew. Chem. Int. Ed.* **2013**, *52*, 6043–6046.

[8] L. Zhou, Y.-B. Shen, X.-D. An, X.-J. Li, S.-S. Li, Q. Liu, J. Xiao, *Org. Lett.* **2019**, *21*, 8543–8547.

[9] K. Matsumoto, S. Takeda, T. Hirokane, M. Yoshida, *Org. Lett.* **2019**, *21*, 7279–7283.

[10] L.-B. Zhang, S.-K. Zhang, D. Wei, X. Zhu, X.-Q. Hao, J.-H. Su, J.-L. Niu, M.-P. Song, *Org. Lett.* **2016**, *18*, 1318–1321.

[11] Bruker, *SAINT, V8.40B*, Bruker AXS Inc., Madison, Wisconsin, USA.

[12] L. Krause, R. Herbst-Irmer, G. M. Sheldrick, D. Stalke, *J. Appl. Crystallogr.* **2015**, *48*, 3–10.

[13] O. V. Dolomanov, L. J. Bourhis, R. J. Gildea, J. A. K. Howard, H. Puschmann, *J. Appl. Crystallogr.* **2009**, *42*, 339–341.

[14] G. Sheldrick, *Acta Crystallogr. C* **2015**, *71*, 3–8.

[15] G. Sheldrick, *Acta Crystallogr. A* **2015**, *71*, 3–8.
